# Calprotectin Increases the Activity of the SaeRS Two Component System and Murine Mortality during *Staphylococcus aureus* Infections

**DOI:** 10.1371/journal.ppat.1005026

**Published:** 2015-07-06

**Authors:** Hoonsik Cho, Do-Won Jeong, Qian Liu, Won-Sik Yeo, Thomas Vogl, Eric P. Skaar, Walter J. Chazin, Taeok Bae

**Affiliations:** 1 Indiana University School of Medicine-Northwest, Gary, Indiana, United States of America; 2 Institute of Immunology, University of Muenster, Muenster, Germany; 3 Department of Pathology, Microbiology, and Immunology, Vanderbilt University School of Medicine, Nashville, Tennessee, United States of America; 4 Department of Biochemistry and Chemistry, and Center for Structural Biology, Vanderbilt University, Nashville, Tennessee, United States of America; National Institutes of Health, UNITED STATES

## Abstract

Calprotectin, the most abundant cytoplasmic protein in neutrophils, suppresses the growth of *Staphylococcus aureus* by sequestering the nutrient metal ions Zn and Mn. Here we show that calprotectin can also enhance the activity of the SaeRS two component system (TCS), a signaling system essential for production of over 20 virulence factors in *S*. *aureus*. The activity of the SaeRS TCS is repressed by certain divalent ions found in blood or neutrophil granules; however, the Zn bound-form of calprotectin relieves this repression. During staphylococcal encounter with murine neutrophils or staphylococcal infection of the murine peritoneal cavity, calprotectin increases the activity of the SaeRS TCS as well as the production of proinflammatory cytokines such as IL-1β and TNF-α, resulting in higher murine mortality. These results suggest that, under certain conditions, calprotectin can be exploited by *S*. *aureus* to increase bacterial virulence and host mortality.

## Author Summary

*Staphylococcus aureus* is an important human pathogen causing skin infections and a variety of life-threatening diseases such as pneumonia, sepsis, and toxic shock syndrome. Previous study showed that the growth of *S*. *aureus* in abscesses is suppressed by the host antimicrobial protein calprotectin, which sequesters Zn and Mn from bacterial usage. During bacterial infection, calprotectin also plays an important role in the production of proinflammatory cytokines. Although the antimicrobial activity of calprotectin has been well defined, it is not known how the proinflammatory property of calprotectin affects staphylococcal infection. In this study, we found that the Zn-binding property of calprotectin increases the pathogenic potential of *S*. *aureus* by enhancing the activity of the SaeRS two component system in *S*. *aureus*. We also found that, under certain infection conditions, the proinflammatory property of calprotectin is rather detrimental to host survival. Our study illustrates that the important antimicrobial protein can be exploited by *S*. *aureus* to render the bacterium a more effective pathogen, and provides an example of the intricate tug-of-war between host and a bacterial pathogen.

## Introduction

*S*. *aureus* is an important Gram-positive human pathogen colonizing the skin, anterior nares and other mucosal surfaces in approximately 30% of the human populations, causing a wide variety of diseases [1]. The pathogenesis of *S*. *aureus* requires multiple virulence factors, and the expression of those virulence factors is controlled by multiple regulatory systems such as SarA family transcription factors, the *agr* quorum sensing system, and the SaeRS two component system (TCS) [2,3].

The SaeRS TCS is composed of the sensor kinase SaeS and the response regulator SaeR along with two auxiliary proteins SaeP and SaeQ [4,5,6]. Conserved in all clinical isolates of *S*. *aureus*, the SaeRS TCS controls the production of more than 20 virulence factors (e.g., hemolysins, leukocidins, coagulases and immune evasion molecules) and plays an essential role in staphylococcal survival and pathogenesis [7,8,9]. *S*. *aureus* appears to use the SaeRS TCS to adapt to hostile host environments such as innate immune responses. The sensor kinase SaeS is activated by human neutrophil peptides (HNPs), small peptides with antimicrobial activity found in the primary granules of human neutrophils [5,10]. In addition, several *sae*-regulated gene products show anti-neutrophil properties [11,12,13,14].

Neutrophils are the most abundant white blood cells in human blood, consisting of 40–70% of the total white blood cell count. As the first line of defense at the site of bacterial infection, neutrophils phagocytose invading bacteria and kill them using reactive oxygen species, granule proteins (including HNPs), enzymatic intracellular degradation, or via neutrophil extracellular traps (NETs) [15,16]. In addition, the neutrophil cytoplasmic protein, calprotectin (CP), has antimicrobial activity toward various infectious fungi and bacteria including *S*. *aureus* [17,18,19]. CP is a heterodimeric S100 class EF-hand Ca-binding protein composed of S100A8 and S100A9 subunits (also called Mrp8/14). In addition to its four Ca binding sites, CP contains two transition metal binding sites S1 and S2 at the subunit interface. S1 can bind to both Zn and Mn whereas S2 binds only Zn [20]. CP is produced primarily by neutrophils and monocytes and released at the sites of inflammation [21,22]. As a main component of NETs and tissue abscesses, its concentration can reach over 1 mg/ml [19]. In tissue abscesses, the sequestration of Zn and Mn by CP suppresses the growth of *S*. *aureus* [19] and impairs the activity of Mn-dependent superoxide dismutases, rendering *S*. *aureus* more susceptible to oxidative stresses [23]. As a ligand for Toll-like receptor 4 (TLR4), CP can amplify inflammatory responses [21,24,25] and increase the migration of neutrophils to inflammation sites without affecting their effector functions [26,27,28].

During our study on the impact of nutrients on the SaeRS TCS activity, we determined that the divalent ions Zn, Fe, and Cu repress the SaeRS TCS. As a result of its ability to bind Zn, we predicted that CP would affect the SaeRS TCS. Therefore, using a clinical isolate of USA300, the predominant PFGE (pulsed-field gel electrophoresis) type of community-associated methicillin-resistant *S*. *aureus* (CA-MRSA) in the United States [29], we examined the role of CP in the activation of the SaeRS TCS. In addition, we also investigated how the proinflammatory property of CP affects host survival during staphylococcal infection. Our results suggest that, during murine peritoneum infection, the antimicrobial protein CP enhances the activity of the SaeRS TCS and, by inducing the production of proinflammatory cytokines, increases host mortality.

## Results

### The SaeRS TCS can be repressed by Cu, Fe, and Zn

**Fig 1:**
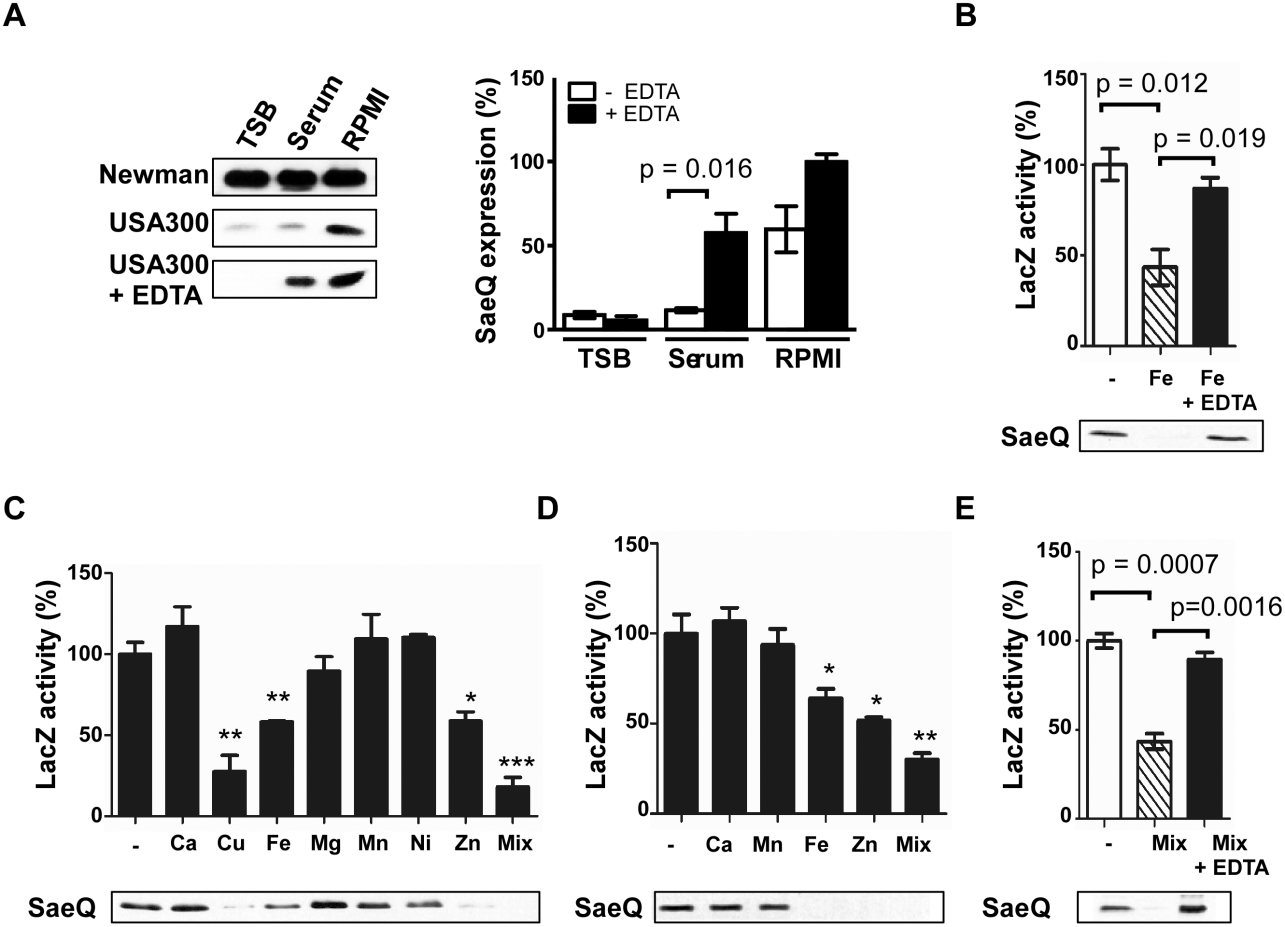
The SaeRS TCS can be repressed by Cu, Fe, and Zn. **(A)** The effect of culture medium on the expression of SaeQ. *S*. *aureus* cells were grown to 0.5 OD_600_, and SaeQ protein was detected by Western blot analysis. Newman, *S*. *aureus* strain Newman; USA300, *S*. *aureus* strain USA300; + EDTA, addition of 1 mM EDTA. The quantification result of the Western blot is shown to the right. **(B)** The effect of iron on the P1 promoter activity (top) and SaeQ expression (bottom). P1 promoter activity was measured by LacZ assay for P1-*lacZ* fusion construct, while SaeQ was measured by Western blot analysis. -, no metal addition. **(C)** The effect of metal ions present in human blood on the P1 promoter activity (top) and SaeQ expression (bottom). *S*. *aureus* cells were grown in RPMI supplemented with 2 mM CaCl_2_, 20 μM CuSO_4_, 20 μM FeSO_4_, 500 μM MgSO_4_, 0.1 μM MnSO_4_, 0.04 μM NiSO_4_, or 20 μM ZnSO_4_ at 37°C for 16 h. Then the P1 promoter activity and SaeQ expression were measured by LacZ assay. Statistical comparison was made against the no metal (-) condition. **(D)** The effect of metal ions present in human neutrophil granules on the P1 promoter activity and SaeQ expression. Cells were grown in RPMI supplemented with 400 μM CaCl_2_, 130 μM FeSO_4_, 130 μM MnSO_4_, or 400 μM ZnSO_4_. All other conditions are the same as in (C). **(E)** The effect of metal chelation on the recovery of the P1 promoter activity (top) and SaeQ expression (bottom). *S*. *aureus* cells were grown for 16 h in RPMI supplemented with all metal ions (Mix) used in (D). The presented data represent three independent experiments. Error bars indicate standard error of the mean. Statistical significance was measured by unpaired, two-tailed *t*-test. *, *p* < 0.05; **, *p* < 0.01; ***, p < 0.001

The activity of the P1 promoter of the *sae* operon and the expression of SaeQ are indicators for the activity of the SaeRS TCS [4,5]. When the expression of SaeQ was analyzed in three different growth conditions, the strain USA300 showed a much higher expression of SaeQ in RPMI (Roswell Park Memorial Institute medium) than in either TSB (tryptic soy broth) or human serum (Fig 1A). The distinct SaeQ expression pattern was not observed with the strain Newman, which carries SaeS L18P, a mutant SaeS with constitutive kinase activity [4,30] (Fig 1A), suggesting that SaeS is responsible for the distinct expression of SaeQ in USA300.

As compared with TSB or human serum, RPMI contains a low level of divalent metal ions [31]. To examine whether the higher SaeQ expression in RPMI is due to the lower content of divalent metals, we added EDTA to the culture media and examined the SaeQ expression. Although no significant change was observed with TSB and RPMI, the SaeQ expression was increased in human serum (Fig 1A), suggesting that, in human serum, divalent metal ions repress the SaeRS TCS. No change in TSB indicates that the SaeRS TCS is suppressed in the growth medium by hitherto unidentified factor(s) different from divalent ions. Addition of Fe to RPMI reduced both the P1 promoter activity and the SaeQ expression, whereas the repression was abolished by EDTA treatment (Fig 1B). To find additional biologically relevant metal ions that repress the SaeRS TCS, various metal ions present in either human blood or neutrophil granules were added to RPMI medium at their physiological concentrations [32,33]. As shown, P1 promoter activity and SaeQ expression were repressed by Cu, Fe, and Zn, and the metal-mediated repression was relieved by EDTA (Fig 1C–1E). This result also confirms the previous report that Cu can repress the SaeRS TCS [34].

### Copper and Zinc inhibit the autokinase activity of SaeS

**Fig 2:**
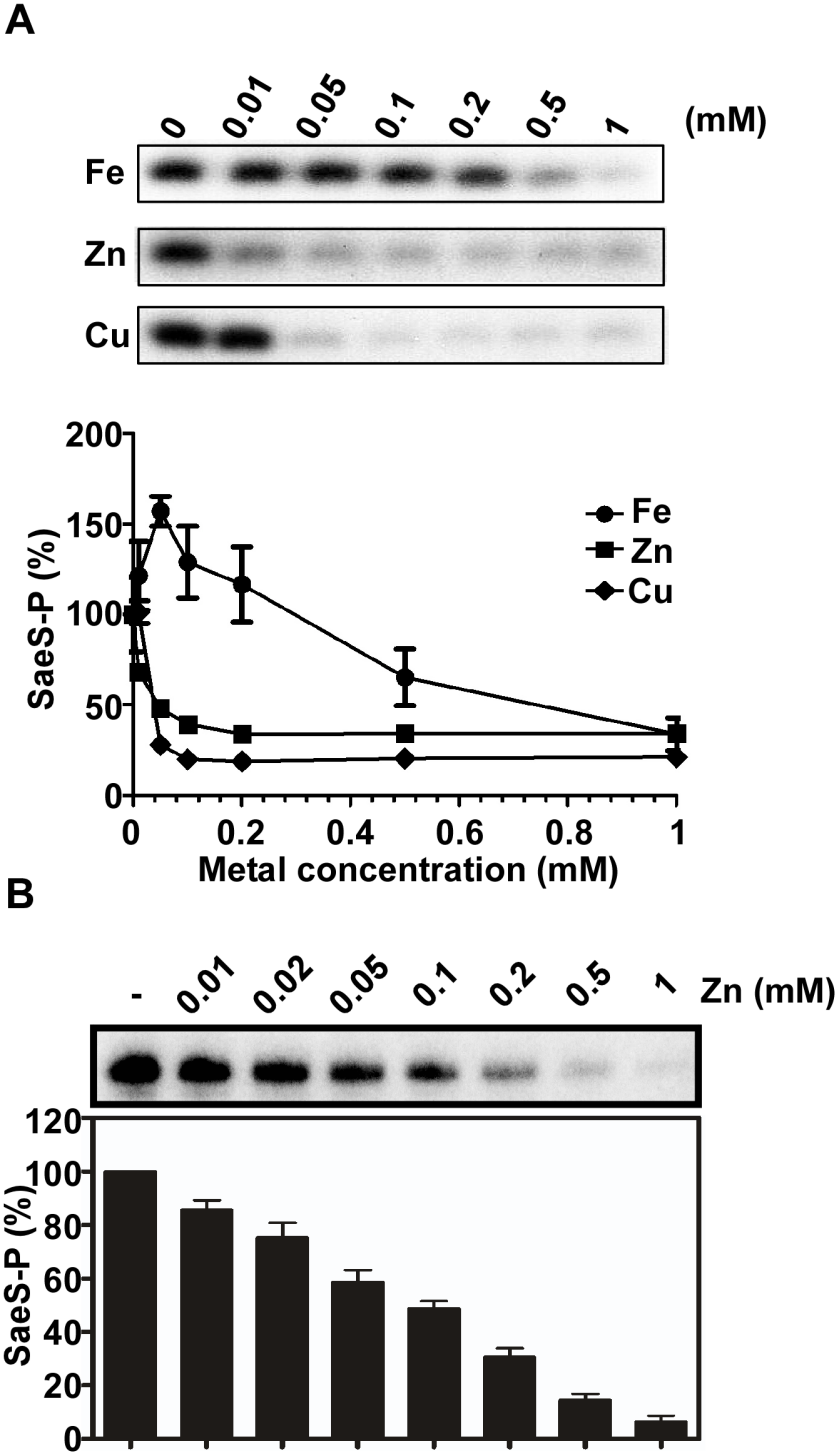
Cu and Zn inhibit the autokinase activity of SaeS. **(A)** The effect of Fe, Zn and Cu on the autokinase activity of SaeS. MBP-SaeS (3 μM) was autophosphorylated with [γ-^32^P]-ATP for 15 min in the presence of various concentrations of FeSO_4_, ZnSO_4_, and CuSO_4_. Then the phosphorylation of SaeS was measured by SDS-PAGE and autoradiograph (top) and quantified (bottom). **(B)** The effect of Zn on the autokinase activity of SaeS in purified cell membranes. Cell membranes (25 μg) containing overexpressed SaeS were used as a source of SaeS. The experiment was carried out as described above. The autoradiograph (top) and quantification of the results (bottom) are shown.

As a sensor kinase, SaeS possesses autokinase, phosphotransferase, and phosphatase activities. To further understand the metal-mediated repression of the SaeRS TCS, we purified MBP (maltose-binding protein)-SaeS and examined the response of the enzymatic activities of SaeS to the metal ions. The autophosphorylation of SaeS was significantly inhibited by 10 μM Zn or 50 μM Cu (Fig 2A), suggesting that the divalent Zn and Cu ions represses the autokinase activity of SaeS. Fe did not inhibit the SaeS autokinase activity until the concentration reaches 500 μM, indicating that, at its physiological concentration, Fe does not inhibit SaeS autokinase activity. Neither the transfer of phosphoryl group from SaeS to SaeR (i.e., phosphotransferase activity) nor the level of phosphorylated SaeR (i.e., phosphatase activity) was affected by the metal ions (S1 Fig), suggesting that Cu and Zn specifically inhibit the autokinase activity of SaeS. Since SaeS is embedded in the cell membrane, we overexpressed SaeS in the strain USA300, purified the cell membranes and repeated the autokinase assay for Zn with the purified cell membranes. As shown in Fig 2B, Zn inhibited the phosphorylation of SaeS in the cell membrane in a concentration-dependent manner.

### CP protects the SaeRS TCS from metal-mediated repression

**Fig 3:**
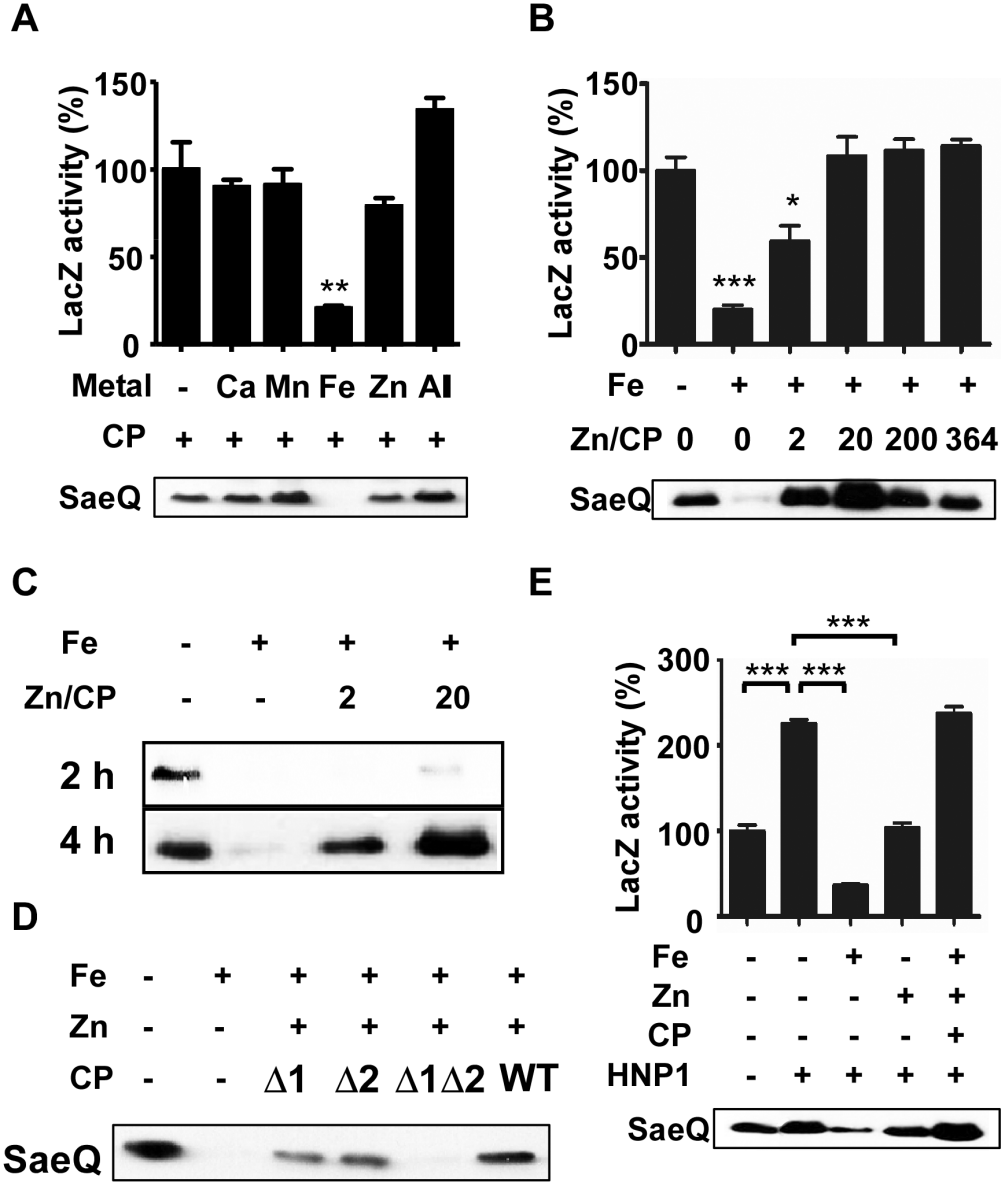
Calprotectin protects the SaeRS TCS from the metal-mediated repression. **(A)** Effect of calprotectin (CP) on the P1 promoter activity and SaeQ expression in the presence of metals found in neutrophil granules. *S*. *aureus* was grown for 16 h in RPMI supplemented with CP (1.1 μM) and the following metal: 400 μM CaCl_2_, 130 μM FeSO_4_, 130 μM MnSO_4_, or 400 μM ZnSO4. -, no metal addition; +, addition. **(B)** Effect of Zn-CP on Fe-mediated repression of the SaeRS TCS. Cells were grown in RPMI to exponential growth phase; then FeSO_4_ (130 μM), CP (1.1 μM), and various concentrations of ZnSO_4_ were added. The cells were further incubated for 16 h. Zn/CP, molar ratio of Zn and CP. **(C)** Effect of incubation time on the restoration of SaeQ expression. Cells were prepared as described above (B) and collected at 2 h and 4 h post incubation. The level of SaeQ was measured by Western blot analyses. **(D)** The effect of Zn-binding on the CP’s protective effect on the SaeRS TCS. Cells were grown to exponential growth phase; then FeSO_4_ (130 μM), ZnSO_4_ (22 μM) and wild type or Zn-binding mutants of CP (1.1 μM) were added. The cells were further incubated for 16 h. Δ1, a mutant CP where the Zn/Mn binding site S1 was abolished; Δ2, a mutant CP where the Zn binding site S2 was abolished; Δ1Δ2, a mutant CP where both S1 and S2 binding sites were abolished. **(E)** The effect of Zn-CP complex on the Fe-mediated repression of the SaeRS TCS in the presence of HNP1. Cells were grown in RPMI to exponential growth phase; then HNP1 (1.5 μM), FeSO_4_ (130 μM), CP (1.1 μM) and ZnSO_4_ (22 μM) were added in the various combinations indicated. The P1 promoter activity (top) and the SaeQ expression (bottom) were measured at 16 h post incubation. Statistical significance was measured by unpaired, two-tailed *t*-test. * *p* < 0.05; ** *p* < 0.01; *** *p* < 0.001.

Since the SaeRS TCS is activated by neutrophils [35], in the following studies, we focused our investigation on Ca, Mn, Fe, and Zn, the major divalent ions found in neutrophil granules [32]. We reasoned that, since CP binds Zn with high affinity, it might be able to reduce the Zn-mediated repression of the SaeRS TCS. To test this possibility, we grew *S*. *aureus* cells in RPMI supplemented with CP and one or all the four metal ions and measured P1promoter activity and SaeQ expression. When the growth medium was supplemented with Fe, as expected, CP failed to restore the activity of the SaeRS TCS (Fig 3A). However, when the medium was supplemented with Zn, CP restored the activity of the SaeRS TCS (Fig 3A), demonstrating that indeed CP can protect the SaeRS TCS activity from the Zn-mediated repression. Intriguingly, when the growth medium was supplemented with all four metal ions, CP restored the SaeRS TCS activity, despite the presence of Fe (Fig 3A). We noted that, in the experiment above, we added 364 times more Zn (400 μM) than CP (1.1 μM) to the growth medium. Therefore, most of Zn ions (> 99%) are expected not to be bound to CP, and simple sequestration of Zn by CP cannot explain the restoration of the SaeRS TCS activity. In addition, despite the fact that CP does not bind to Fe, when CP was added to the growth medium supplemented with all the four metal ions, it restored SaeRS TCS activity (Fig 3A). Based on these results, we hypothesized that, by binding to Zn, CP gains the ability to protect the SaeRS TCS not only from Zn but also from Fe. To test this hypothesis, we grew the strain USA300 in RPMI, added Fe and Zn-CP mixture of various ratios (0–364), and then measured the SaeRS TCS activity. Indeed, CP restored the SaeQ expression only when Zn was also present (Fig 3B), and the restoration of SaeQ expression required more than 2 h incubation (Fig 3C). Moreover, a mutant CP incapable of binding to Zn failed to protect the activity of the SaeRS TCS, while CP mutants retaining one binding site still protected [20] (Fig 3D). These results suggest that binding of Zn confers CP with the ability to protect the SaeRS TCS from repression by Zn and Fe.

Since the SaeRS TCS is activated by human neutrophil peptides (HNPs) [5], we examined the effect of Zn-CP on the HNP1-mediated activation of the SaeRS TCS. As shown in Fig 3E, Fe inhibited the HNP1-mediated activation of the SaeRS TCS; however, the addition of Zn-CP abolished the inhibition by Fe, and the P1 activity and SaeQ expression were rather further increased. Based on these observations, we concluded that CP and HNP1 can cooperatively increase SaeRS TCS activity.

### CP induces global changes in staphylococcal transcriptome

**Fig 4:**
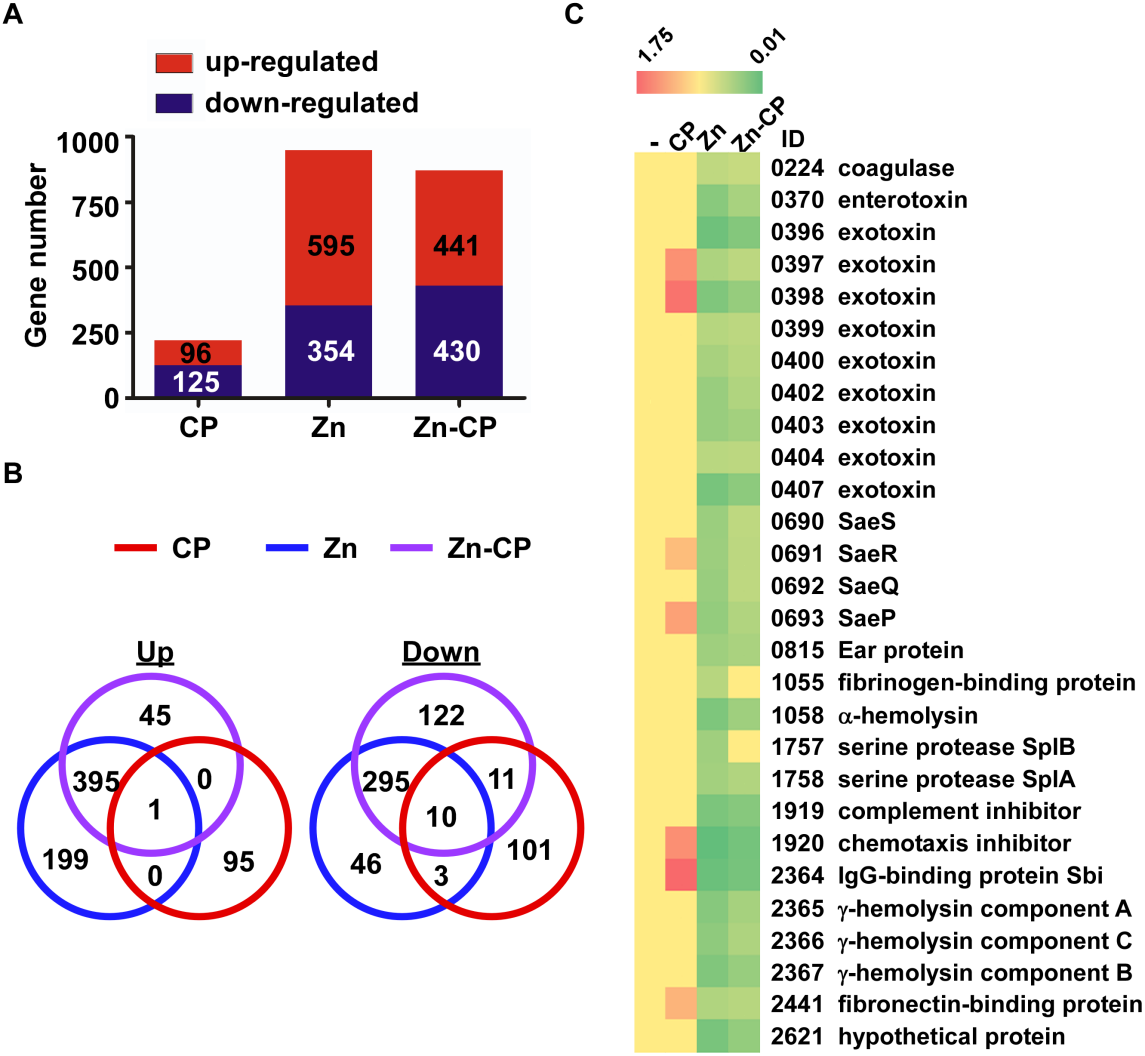
RNA-seq analysis of the effect of CP, Zn and Zn-CP on staphylococcal transcriptome. *S*. *aureus* USA300 cells at exponential growth phase were treated by CP (1.1 μM), Zn (20 μM) or Zn (20 μM)-CP (1.1 μM) at 37°C for 4 h; then the transcript levels were analyzed by RNA-seq. **(A)** The number of genes affected by CP, Zn, and Zn-CP. **(B)** Venn diagram analysis of the genes affected by CP, Zn, and Zn-CP. Up, up-regulated genes; Down, down-regulated genes. **(C)** Effect of CP, Zn, and Zn-CP on the transcript levels of the *sae* regulon. -, no treatment. ID, Gene ID in the genome of the strain USA300_FPR3757.

To understand the effect of CP and Zn on staphylococcal gene expression, we treated the strain USA300 at its exponential growth phase with CP, Zn, or Zn-CP and assessed the transcriptome changes at 4 h post treatment by RNA-seq analysis. The CP treatment altered the transcript level of 221 genes (Fig 4A, S1 and S2 Tables), indicating that the effect of CP is global and not limited to the SaeRS TCS. The Zn and Zn-CP treatments induced profound changes in the staphylococcal gene expression affecting 949 and 871 genes, respectively (Fig 4A, S3–S6 Tables). The Zn and Zn-CP treatments shared 396 (up-regulated) and 305 (down-regulated) genes (Fig 2B), indicating that, when Zn and CP were added together, Zn exerts a dominant effect on the staphylococcal transcription. The dominant effect of Zn over CP is also corroborated by the principal component analysis (S2 Fig). Nonetheless, the addition of CP to Zn also elicited up-regulation of 45 genes and down-regulation of 133 genes, which was not observed in the Zn-treated cells (Fig 4B). When only the *sae* regulon was analyzed, CP up-regulated 7 genes, while Zn down-regulated 28 genes (Fig 4C), confirming the positive and negative effects of CP and Zn on the SaeRS TCS. When treated with Zn-CP, overall, the *sae* regulon remained repressed, as compared with control medium condition (Fig 4C). However, as compared with Zn treatment, all genes showed a higher level of transcripts to disparate extents. In particular, two genes, SAUSA300_1055 and 1757, showed transcript levels similar to that in the control medium (Fig 4C). When compared with Zn treatment, the Zn-CP treatment significantly up-regulated 14 genes and down-regulated 43 genes (S7 and S8 Tables). Of the 14 up-regulated genes, 10 contain the SaeR binding sequence in their promoter region (S7 Table), indicating that, although the CP induces global changes in staphylococcal gene expression, the protective effect in the presence of Zn is rather specific to the SaeRS TCS.

### CP enhances the SaeRS TCS activity and alters cytokine production in a murine neutrophil infection model

**Fig 5:**
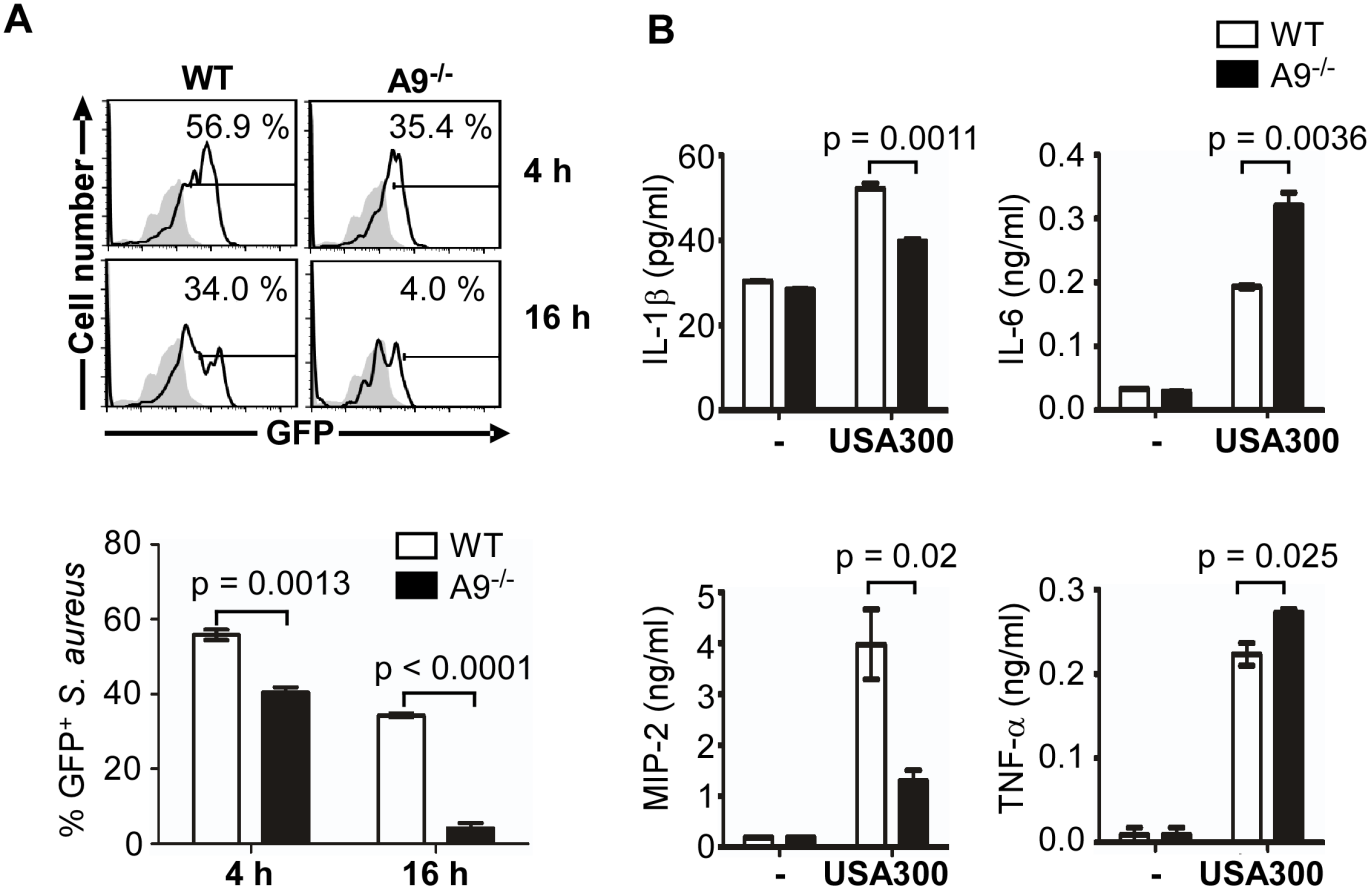
CP is required for full activation of the SaeRS TCS and cytokine production by murine neutrophils. **(A)** Activation of the SaeRS TCS by neutrophils from C57BL/6 (WT) and C57BL/6 S100A9 ^-/-^ (A9^-/-^) mice. Neutrophils purified from bone marrow of WT or A9^-/-^ mice were mixed with *S*. *aureus* strain USA300 containing P1-*gfp* reporter plasmid (MOI = 10). At the indicated time points, neutrophils were lysed, and the P1 promoter activity was measured by flow cytometry (top panel), and the results were also presented in a bar graph (bottom panel). In the flow cytometry analysis, gray color represents the results from the control plasmid (pCL-*gfp*). In the bar graph, error bars depict standard error of the mean. Results are from three pooled mice per genotype and represent three independent experiments. **(B)** The effect of CP in the cytokine production of neutrophil. Neutrophils purified from bone marrow of WT and S100A9^-/-^ mice were infected with *S*. *aureus* USA300 (MOI = 10) for 2 h. After addition of gentamicin, neutrophils were further incubated for 16 h, and the concentration of the cytokines indicated was determined by ELISA. The data are from three pooled animals per genotype and representative of three independent experiments. Error bars indicate standard error of the mean. Statistical significance was determined by unpaired, two tailed *t*-test.

To investigate the role of CP in the activation of the SaeRS TCS by neutrophils, we generated a GFP reporter system for the P1 promoter and integrated it in the chromosome of the strain USA300 (S1A Fig). The resulting reporter strain showed significantly higher GFP signal, as compared with no-promoter control (S3B Fig), and responded to the repression by Fe and Zn (S3C Fig). When the reporter strain was mixed with murine neutrophils purified from either wild type or CP-deficient mice, a higher GFP signal was observed in the presence of wild type neutrophils at 4 h post incubation, and it was more pronounced at 16 h (Fig 5A), suggesting that CP indeed contributes to the activation of the SaeRS TCS during encounter with murine neutrophils.

As an endogenous ligand for TLR4, CP amplifies the endotoxin-induced secretion of TNF-α and other proinflammatory cytokines [24,36]. In addition, the SaeRS TCS is reported to induce the production of proinflammatory cytokines including interferon-gamma (IFN-γ) from murine neutrophils [37,38]. To examine the role of CP in cytokine production during staphylococcal infection, we measured proinflammatory cytokines released by murine neutrophils at 16 h post infection. In the CP-deficient mice, the production of IL-1β and MIP-2 was decreased while that of IL-6 and TNF-α was increased (Fig 5B), indicating that CP can affect the production of proinflammatory cytokines by murine neutrophils.

### CP does not affect the migration or bacterial killing of murine neutrophils

Since CP was required for full activation of the SaeRS TCS, we further asked the question whether or not CP also affects the function of neutrophils, notably migration and bacterial killing. Flow cytometry analysis showed no significant difference in neutrophil recruitment at the infection site (S4A Fig). The number of recruited neutrophils was also comparable between the two mice strains (S4A Fig). In addition, upon contact with *S*. *aureus*, both wild type and the CP-deficient neutrophils formed neutrophil extracellular traps (NETs) [15] at a similar efficiency (S4B Fig), resulting in equivalent secretion of DNA (S4C Fig). We also found that both wild type and the CP-deficient neutrophils killed the bacteria with a similar efficiency (S4D Fig). Taken together, these results demonstrate that CP does not affect either migration or the bactericidal activities of neutrophils.

### CP affects not only the activation of the SaeRS TCS but also the cytokine production during murine infection by *S*. *aureus*

**Fig 6:**
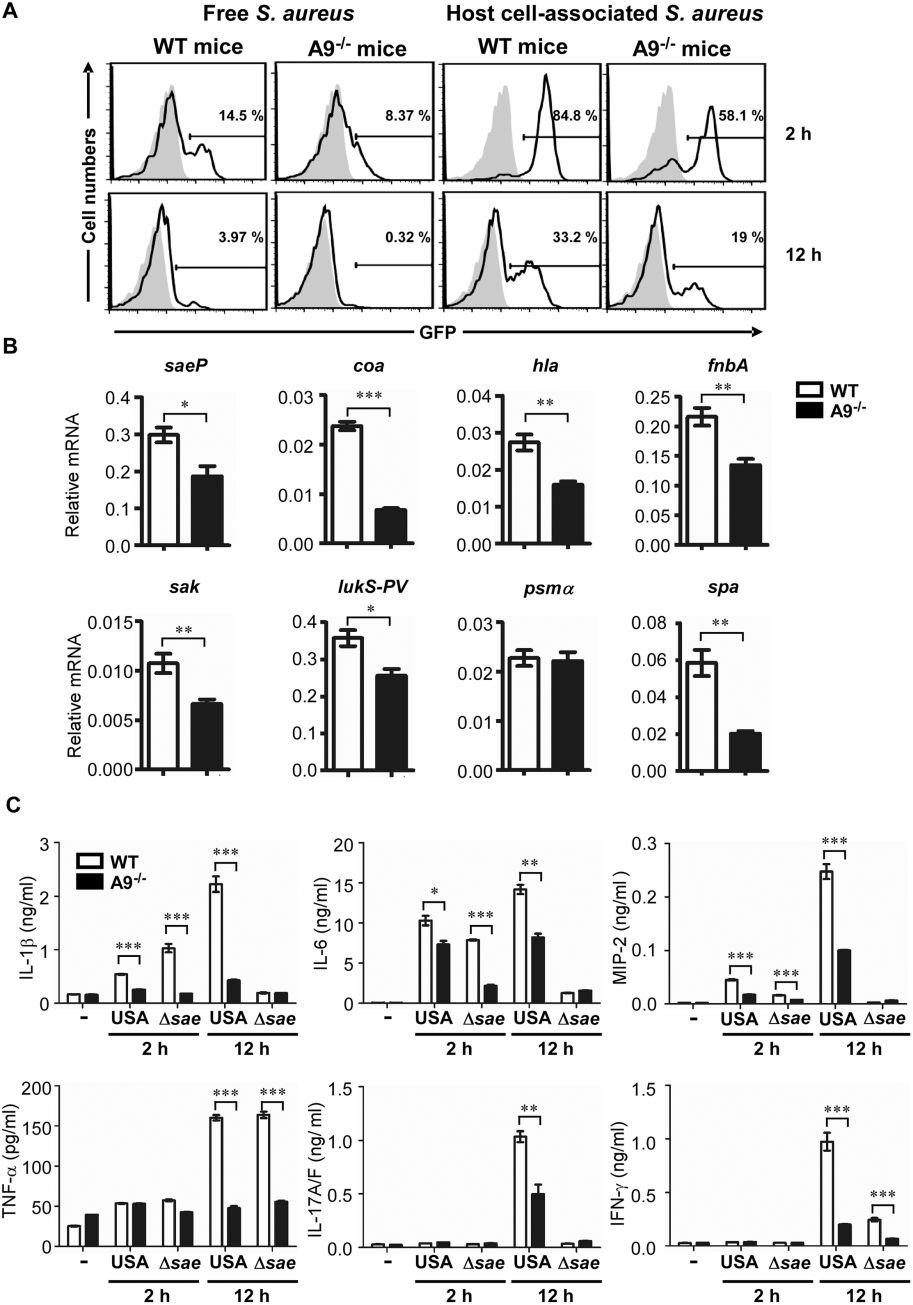
CP enhances the activity of the SaeRS TCS and cytokine production in mice. **(A)** The activity of the SaeRS TCS in C57BL/6 (WT) and C57BL/6 S100A9^-/-^ (A9^-/-^) mice. Mice were infected with *S*. *aureus* (2 × 10^8^ cfu) by peritoneal injection, and peritoneal fluid was acquired at the indicated time points. After being separated by centrifugation (200 ×*g*), the GFP expression of *S*. *aureus* in supernatant (host cell-free) or in pellet (host cell-associated) was measured by flow cytometry (top panel), where the gray color represents GFP expression from pCL-*gfp*. The quantification results are shown in S5 Fig. **(B)** The effect of CP on the expression of select genes. **(C)** The production of proinflammatory cytokines in wild type (WT) and S100A9^-/-^ (A9^-/-^) mice upon infection with *S*. *aureus* USA300 (USA) or the *sae* deletion mutant (Δ*sae*). At the time points indicated, mice were sacrificed and the concentrations of proinflammatory cytokines in peritoneal fluids were determined by ELISA. The data are from three pooled mice per genotype and represent three independent experiments. Error bars indicate standard error of the mean. -, no bacteria. Statistical significance was assessed by *t*-test. * *p* < 0.05; ** *p* < 0.01; *** *p* < 0.001

To further investigate the role of CP in the activation of the SaeRS TCS *in vivo*, we infected wild type and the CP-deficient mice with the P1-*gfp* reporter strain via intraperitoneal injection; then the GFP signal was measured in the host cell-free or host cell-associated fraction of peritoneal fluid by flow cytometry. Wild type mice showed a higher GFP signal than did CP-deficient mice in both host cell-free and host cell-associated fractions at both 2 h and 12 h post infection (Fig 6A and S5 Fig), confirming that CP contributes to the activation of the SaeRS TCS during staphylococcal infection. Host cell-associated fraction showed 6–20 times higher GFP signal than host cell-free fraction, indicating that the SaeRS TCS is activated mainly upon contact with host cells. In addition, the GFP signal at 2 h was 2–4 times higher than that at 12 h, suggesting decreased SaeRS activity or bleaching of GFP-signals [39,40]. To confirm the higher activity of the SaeRS TCS in the wild type mice, we collected *S*. *aureus* from peritoneal fluid at 12 h post infection; then the transcription of the known *sae* target genes (*saeP*, *coa*, *hla*, *fnbA*, *sak*, and *lukS-PV*) and two non-*sae* target genes (*psmα* and *spa*) were analyzed. Indeed, all *sae* target genes showed lower transcription in CP-deficient mice (Fig 6B). Of the two non-*sae* targets, the transcription of *psmα* was not affected by CP; however, the transcription of *spa* was significantly decreased in the CP-deficient mice, indicating that the effect of CP is not limited to the SaeRS TCS.

CP has two important qualities: metal chelation and proinflammatory properties. Whereas it is known that CP restricts staphylococcal growth in the abscess and confines spread of the bacterium through nutrient metal chelation, it is not known how the proinflammatory properties affect staphylococcal pathogenesis. Therefore, we first measured the production of six proinflammatory cytokines during staphylococcal peritoneal infection. Indeed, as compared with CP-deficient mice, the wild type mice produced significantly higher levels of proinflammatory cytokines: IL-1β, IL-6, and MIP-2 at 2 h, and all 6 cytokines at 12 h post infection (Fig 6C). When infected by the Δ*sae* mutant strain, both mouse strains showed much lower production of proinflammatory cytokines, except for TNF-α, at 12 h post infection (compare USA and Δ*sae* at 12 h in Fig 6C), confirming that the products of the *sae* regulon also contribute to the production of proinflammatory cytokines[37].

### CP increases murine mortality during staphylococcal infections

**Fig 7:**
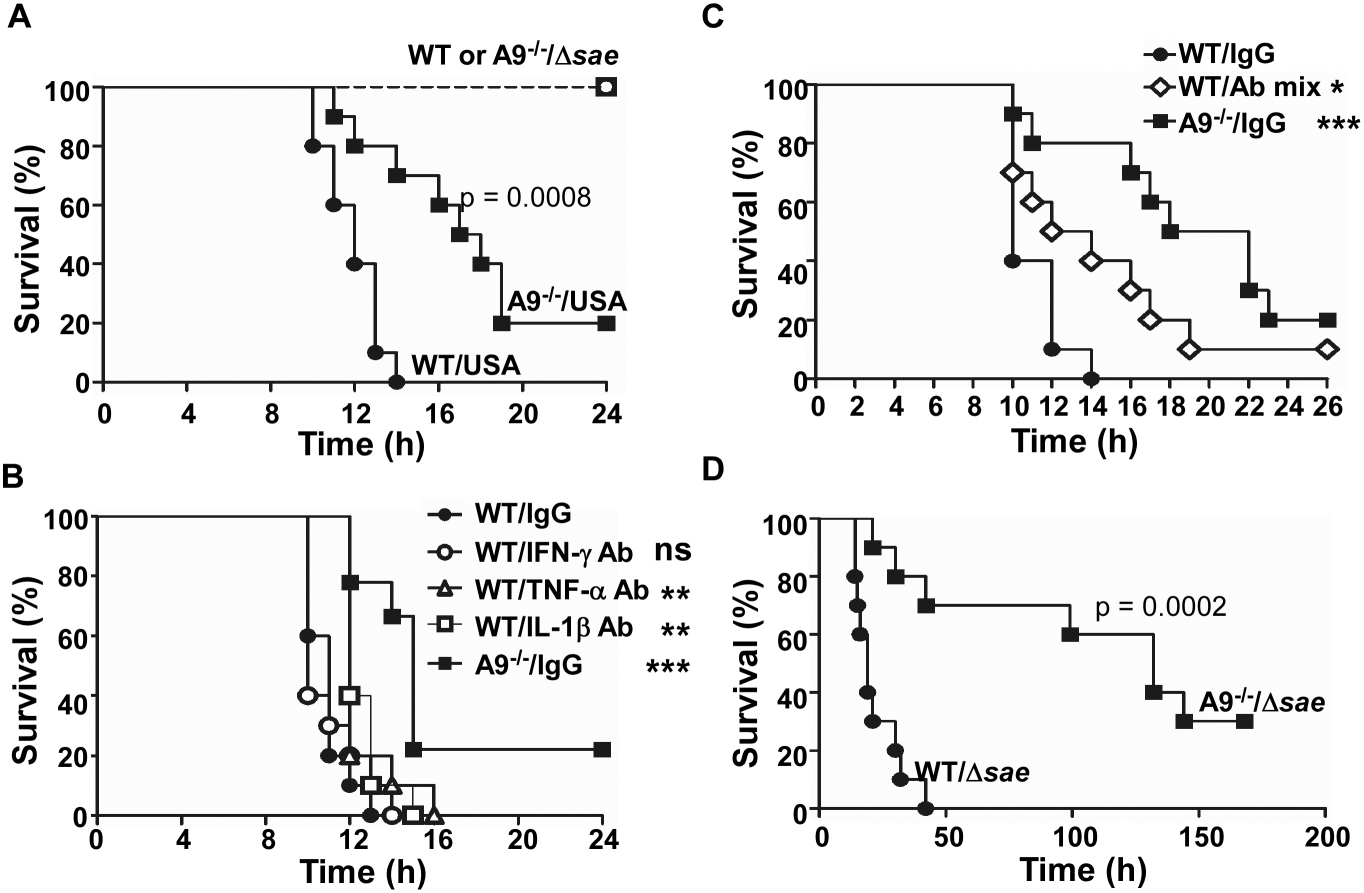
The proinflammatory property of CP increases murine mortality. **(A)** Effect of CP on the survival of mice infected by *S*. *aureus*. C57BL/6 (WT) or C57BL/6 S100A9^-/-^ (A9^-/-^) mice were infected with USA300 (USA) or *sae*-deletion mutant (Δ*sae*) by intraperitoneal injection (2×10^8^ cfu). Ten mice were used for each test group. Statistical significance was assessed by Log-rank (Mantel-Cox) test. **(B)** Effect of individual proinflammatory cytokine antibodies on the murine survival. WT or A9^-/-^ mice were infected with the wild type USA300 (2 × 10^8^ cfu). At 2 h post infection, 100 μg of the indicated antibody was injected via i.p. route. **(C)** Effect of proinflammatory cytokine antibody mixture on the murine survival. WT or A9^-/-^ mice were infected with the wild type USA300 (2 × 10^8^ cfu). At 2 h post infection, 150 μg of proinflammatory cytokine antibody (50 μg of each IFN-γ Ab, TNF-α Ab, and IL-1β Ab) was injected via i.p. route. **(D)** CP can increase murine mortality in the absence of the SaeRS TCS. WT or A9^-/-^ mice were infected with the *sae*-deletion mutant (Δ*sae*, 1 × 10^9^ cfu). Statistical significance was assessed by Log-rank (Mantel-Cox) test. ns, not significant; **, p < 0.01; ***, p < 0.001

Since CP enhanced the activity of the SaeRS TCS and the production of several proinflammatory cytokines, both of which can be detrimental to the survival of the host, next we examined the effect of CP on the mortality of the infected mice. When infected with wild type USA300, all wild type mice died by 14 h post infection, whereas 70% of CP-deficient mice were alive (Fig 7A). At 24 h post infection, 20% of CP-deficient mice were still alive. These results suggest that indeed CP is detrimental for murine survival during staphylococcal peritoneal infection. On the other hand, when infected with the Δ*sae* mutant, no mice died, regardless of the genetic background (Fig 7A), demonstrating the importance of the SaeRS TCS in the bacterial virulence. To test whether the detrimental effect of CP on host survival depends on infection route, we administered *S*. *aureus* cells into mice via retro-orbital injection. When infected with wild type USA300, only 10% of wild type mice survived by day 14, whereas yet 60% of CP-deficient mice were still alive (S6 Fig). When infected by Δ*sae* mutant, all mice survived again (S6 Fig). This observation demonstrates that the increased mortality of wild type mice infected with *S*. *aureus* USA300 is dependent on CP but independent of the infection routes.

### Increased production of proinflammatory cytokines contributes to the higher mortality of the wild type mice

To examine the role of the proinflammatory property of CP in the increased murine mortality, we administered antibodies against IFN-γ, IL-1β, or TNF-α at 2 h post infection and compared the murine mortality. No significant effect was observed with anti- IFN-γ antibody (Fig 7B). However, the administration of either anti-IL-1β or anti-TNF-α antibody caused a small but statistically significant delay in the death of the infected mice (Fig 7B). When the mixture of all three antibodies was administered, the delay of the death was much more pronounced, and infected mice survived significantly longer (Fig 7C). These results demonstrate that the proinflammatory property of CP is, at least in part, responsible for the higher mortality of the wild type mice.

*S*. *aureus* contains multiple proinflammatory PAMP (pathogen-associated molecular pattern) molecules such as lipoproteins, lipoteichoic acid (LTA), and peptidoglycan [41,42,43,44]. To examine whether CP can increase murine mortality in the absence of the SaeRS TCS, we infected the wild type and CP-deficient mice with the *sae*-deletion mutant at 5 times increased dosage. As shown in Fig 7D, the CP-deficient mice still showed a lower mortality than wild type mice, demonstrating that, in the presence of excess PAMP molecules, the CP can increase murine mortality in a *sae*-independent fashion.

## Discussion

CP is a multi-functional protein with a broad range of antimicrobial activities and proinflammatory properties. In particular, its antimicrobial activities against *S*. *aureus* are well documented. In this study, however, we show that, in certain infection conditions, the antimicrobial activity of CP can lead to the activation of the SaeRS TCS and the proinflammatory property of CP can increase murine mortality.

Among the metal ions present in either serum or neutrophil granules, Cu, Fe, and Zn were able to repress the SaeRS TCS. How then do the metal ions repress the SaeRS TCS? Like other sensor histidine kinase, SaeS requires Mg as a cofactor for enzymatic activities. Enzyme assays show that Cu and Zn can inhibit the autokinase function of SaeS, while Fe does not (Fig 2A). The radius of divalent Fe (0.78 Ǻ) is larger than that of Mg (0.72 Ǻ), whereas the radii of divalent Zn (0.74 Ǻ) and Cu (0.73 Ǻ) are more similar to that of Mg [45]. Hence Zn and Cu may inhibit the SaeS autokinase activity by displacing Mg in the catalytic center, although conformational changes or allosteric effects are also equally possible. Since Fe did not inhibit either autokinase or phosphotransferase activity of SaeS, it is likely that the Fe inhibits the SaeRS TCS indirectly. This indirect effect of Fe on the SaeRS TCS is supported by the fact that the SaeRS TCS is also repressed by haemin [46]. Johnson et al reported that Fur, a transcription regulator responding to iron availability, is required for expression of multiple *sae* regulon in low-iron growth condition [47] raising the possibility that Fur mediates the Fe-mediated repression of the SaeRS TCS. However, we observed that Fe can repress the expression of SaeQ in a *fur* mutant of USA300 (S7 Fig), ruling out the involvement of Fur in the Fe-mediated repression of the SaeRS TCS. Therefore, it still remains to be determined how Fe suppresses the SaeRS TCS.

CP shows its protective effect only when Zn is present (Fig 3A–3C). In addition, the mutant CP lacking Zn binding sites failed to restore the SaeRS TCS activity (Fig 3D), suggesting that the Zn-bound CP, not the Zn-free CP, protects the SaeRS TCS from the metal-mediated repressions. Since it takes more than 2 h to show its protective effect (Fig 3C), it is likely that the Zn-bound CP protects the SaeRS TCS indirectly. SaeS has a linker peptide of 9 amino acids between its two transmembrane helices [4] and is thought to respond to physicochemical changes in the cell membrane. Due to the small size of the linker peptide, the entire sensor domain (i.e., two membrane helices and the linker peptide) is expected to be buried in the membrane and interact intimately with membrane lipids and surface molecules. For instance, Omae et al recently reported that the apolipoprotein from silkworm represses the SaeRS TCS by binding LTA [48], indicating that LTA can affect the SaeR TCS activity via direct interaction with SaeS. Indeed, mutational changes in the extracellular linker peptide altered the SaeS response to LTA [48], suggesting that the linker peptide is involved in the interaction with LTA. By analogy, it is possible that the Zn-bound CP renders SaeS resistant to the metal-mediated repression by binding to surface molecules in the cell envelope. However, since it takes more than 2 h for the protective effect by Zn-CP to occur (Figs 3C and 4C), we suspect that the surface molecule, if there is any, does not directly interact with SaeS and, instead, it might cause alteration of the cell membrane environment of SaeS (e.g., the compositions of membrane proteins and lipids), in which SaeS might be protected from the metal-mediated repression. This hypothesis is clearly speculative and requires experimental verification.

During peritoneal infection by *S*. *aureus*, proinflammatory cytokines play an important role in the CP-mediated increase of murine mortality (Fig 7B and 7C). Since CP enhanced not only the SaeRS TCS activity but also the production of proinflammatory cytokines (Fig 6), and the SaeRS TCS also played a key role in the production of five out of six proinflammatory cytokines tested in this study (Fig 6C), it is likely that the reduced activity of the SaeRS TCS in CP-deficient mice contributed to the higher survival of the mice (Fig 7A). In fact, when wild type mice were infected with a mutant *S*. *aureus* strain where the expression of the *sae* target genes was reduced by 70%, the survival rate of the infected mice was improved by 40% -70% [49]. Therefore, it is expected that the 30%- 40% reduction of the *sae* activity in the CP-deficient mice (Fig 6A) also contributed to the survival of the mice (Fig 7A).

Recently Watkins et al showed that, during peritoneal infection by *S*. *aureus*, the SaeRS TCS induces the production of IFN-γ from murine neutrophils [38]. Although we confirmed the critical role of the SaeRS TCS in the production of IFN-γ in mice (Fig 6C), we did not find any evidence that purified murine neutrophils can produce IFN-γ. Instead, we found that the SaeRS TCS plays a significant role in the production of not only IFN-γ but also IL-1β, IL-6, MIP-2, and IL-17A/F during murine infection (Fig 6C). It is known that the *sae*-regulated cytolytic toxin Hla (alpha-hemolysin or alpha-toxin) activates the NLRP3 (nucleotide binding domain and leucine rich repeat containing protein)-inflammasome pathway in human and murine monocytic cells [50] and induces the production of IL-1β, IL-6, and IL-8 [50,51,52,53]. Since the *sae*-regulon also includes many lipoproteins [7], an important TLR2 ligand, the SaeRS TCS is expected to induce proinflammatory cytokine production via TLR2. In addition, some staphylococcal super antigen-like (SSL) proteins are expected to be regulated by the SaeRS TCS [7,54]. Therefore, we believe all of these *sae*-target gene products contribute to the cytokine production during staphylococcal infection. In addition, it is possible that the cellular damages induced by the *sae* regulons also contributed to the proinflammatory host response.

*S*. *aureus* is well known for its exploitation of host factors to promote bacterial survival. During host tissue invasion, *S*. *aureus* activates prothrombin with two coagulases, Coa and vWbp, and surrounds its cell mass with fibrin-deposit called pseudocapsule, blocking the access of neutrophils [11,55]. *S*. *aureus* uses the nuclease Nuc to degrade DNA in NETs, and induces apoptosis of macrophages by converting the released nucleotides into deoxyadenosine [56]. The SaeRS TCS is essential for these host exploitations because all of those bacterial factors (i.e., Coa, vWbp, and Nuc) are the products of the *sae* regulon [7,8,57]. In this study, we add the neutrophil cytoplasmic protein CP to the list of the host factors exploited by *S*. *aureus*. CP is important to restrict growth through metal chelation in abscesses; however, our data also suggest that, during high dose systemic infections, its antimicrobial activity increases the activity of the SaeRS TCS and its proinflammatory properties can lead to higher mortality of the host, shedding new light on the evolutionary tug-of-war between microbial pathogens and host.

## Materials and Methods

### Ethics statement

The animal experiment was performed by following the Guide for the Care and Use of Laboratory Animals of the National Institutes of Health. The animal protocol was approved by the Committee on the Ethics of Animal Experiments of the Indiana University School of Medicine-Northwest (Protocol Number: NW-34). Every effort was made to minimize suffering of the animals.

### Bacteria strains and culture conditions

The bacterial strains and plasmids used in this study are listed in S9 Table. *Escherichia coli* was grown in Luria-Bertani broth (LB) medium while *S*. *aureus* was grown in tryptic soy broth (TSB), human serum (Sigma-Aldrich), or RPMI 1640(Corning) with shaking (250 rpm). To measure bacterial growth in human serum, cells were washed twice and suspended in phosphate buffered saline (PBS) before OD_600_ measurement. When necessary, antibiotics were added to the growth media at the following concentrations: ampicillin, 100 μg/ml; erythromycin, 10 μg/ml; and chloramphenicol, 5 μg/ml.

### Construction of plasmids

To generate the P1 promoter-*gfp* reporter plasmid, the *gfp* gene was PCR-amplified from pSW4-GFP_opt_ [58]by Phusion DNA polymerase (NEB) with the primer pairs P974 (5’- GATGGTACCAAA AGGAGAACGCATAATGTCAAA AG-3) and P975 (5’- CGGGCT CCGCGGGCAGCCGAAT TCTTACCCCCCG-3’). The amplified fragments were digested with *Kpn*I. The resulting PCR product was ligated with the integration plasmid pCL55 digested with *Kpn*I and *Sma*I, resulting in pCL-*gfp*. The P1 promoter sequence was PCR-amplified by Phusion DNA polymerase (NEB) with the primer pairs P1096 (5’-AACGGTACCTTGGTACTTGTATTTAATCGTCTATC-3’) and P785 (5’- AAAGGTACCGTTGTGATAACAG CACCAGCTGC-3’). The PCR product was digested with *Kpn*I and inserted into pCL-*gfp* digested with *Kpn*I. The plasmid with correctly oriented P1 sequence was identified by PCR analysis and named pCL-P1*gfp*. pCL-*gfp* and pCL-P1*gfp* were electroporated into *S*. *aureus* strain RN4220 and then transduced into USA300-P23 with ϕ85.

To generate the SaeS overexpression plasmid pYJ-*saeRS*, the *saeRS* region was PCR-amplified with the primers P2856 (5’- GAGTATAATTAAAATAAGCTTGAT AGAGGTGAAAAAATAGATGACCCACTTACT-3’) and P2857 (5’-AACGACGGCCAGTGAATTCGAGCTCGGTACCCG CGGTTATGACGTAATGTCT-3’) using pCL-*saeRS* as a template [59]. The PCR product was digested with *EcoR*V and *Kpn*I, and cloned into pYJ335 digested with the same enzymes[60].

### β-galactosidase assay

Cells carrying the plasmid pCL-P1-*lacZ* [7] were grown and collected at a desired time point by centrifugation. The collected cells were washed with AB buffer (60 mM K_2_HPO_4_, 40 mM KHPO_4_, 100 mM NaCl, pH 7.0), suspended in 100 μl of AB buffer, and mixed with 5 μl of lysostaphin (2 mg/ml). After 15 min incubation at 37°C, the samples were mixed with 900 μl of AB buffer containing 0.1% (v/v) Triton X-100, and the β-galactosidase assay was performed at room temperature. As a substrate, 4-methyl umbelliferyl β-D galactopyranoside (MUG, Sigma) was used in the hydrolysis reaction, which was read at 366 nm excitation and 445 nm emission wavelengths.

### Western blot analysis of SaeQ

*S*. *aureus* cells were collected by centrifugation, and normalized to 1 ml of 1.0 OD_600_. Western blot analysis of SaeQ was carried out as described previously [61]. Briefly, cells were collected by centrifugation and suspended in 50 μl of Tris HCl (pH 8.0); then 2 μl lysostaphin (2 mg/ml) was added. After incubation at 37°C for 30 min, 50 μl of 2× SDS-PAGE sample buffer was added. The samples were separated by SDS-PAGE and the proteins were transferred onto a nitrocellulose membrane (0.45 μm, Whatman). The membrane was blocked with 10% skim milk and incubated with SaeQ antibody for 1 h at room temperature; then the blot was incubated with horseradish peroxidase (HRP)-conjugated secondary antibody. Signals were detected by a luminal enhancer solution detection kit (Thermo).

### Metal/calprotectin effects on the SaeRS TCS

Unless indicated otherwise, strains were grown in RPMI for 16 h in the presence or absence of the metal ions present in human blood or in neutrophil granules. When necessary, CP (1.1 μM) or HNP1 (5 μg/ml, Bachem) was added to the growth medium. Recombinant CP was expressed, purified, and tested for activity as described previously [20].

### SaeS autophosphorylation

Maltose-binding protein fused SaeS (MBP-SaeS) was expressed in *E*. *coli* BL21star(DE3) and purified with MBPTrap HP column (GE Healthcare) by following the column manufacturer’s recommendations. The purified MBP-SaeS (3 μM) was suspended in the reaction buffer (10 mM Tris-HCl, pH 7.4, 50 mM KCl, 10 μM MgCl_2_, 10% glycerol) containing various concentrations (0–1 mM) of metal ions (FeSO_4_, ZnSO_4_, CuSO_4_,). After addition of [γ-^32^P]-ATP (2 μCi), the samples were incubated at room temperature for 15 min and subjected to SDS-PAGE (10%) and autoradiography.

### Phosphoryl transfer reaction

First, MBP-SaeS was phosphorylated as described above in the reaction buffer. After elimination of free [γ-^32^P]-ATP with a Micro Bio-Spin Chromatography Column (Bio-Rad), SaeR (9 μM) and various concentrations (0–0.5 mM) of metal ions were added. The resulting samples were incubated at room temperature for 15 min and subjected to SDS-PAGE (13%) and autoradiography.

### SaeS autophosphorylation assay with purified cell membranes

*S*. *aureus* strains harboring pYJ-*saeRS* were grown to exponential growth phase at 37°C and the SaeS protein was induced by the addition of anhydrotetracycline (Clontech, 0.5 μg/ml) at 37°C for an additional 4 h. Cell membranes were prepared as described previously [49]. The SaeS phosphorylation assay was carried out as described above except that the purified cell membranes (25 μg) were used as a source of SaeS and the incubation time was 10 min.

### RNA-seq analysis

Cells were grown in RPMI to exponential growth phase; then ZnSO_4_ (20 μM), CP (1.1 μM), or the mix of ZnSO_4_ (20 μM) and CP (1.1 μM) was added to the culture. After 4 h incubation of the culture at 37°C, total bacterial RNA was isolated using the RNeasy minikit (Qiagen) with optional on-column DNA digestion according to the manufacturer’s instructions. After purification, contaminating DNA was removed with RNase-free DNase I. RNA was then purified again using RNeasy Mini columns. The purified RNA was sent to the Center for Genomics and Bioinformatics at Indiana University. Sequencing libraries were constructed using the ScriptSeq Complete Kit for Bacteria (Epicentre).

### Isolation of bone marrow-derived mouse neutrophils

Bone marrow-derived neutrophils were isolated as described previously [62]. Briefly, mice were euthanized by CO_2_ asphyxiation; then tibias and femurs were flushed with Hank's balanced salt solution without Ca^2+^ and Mg^2+^ (HBSS). After lysing red blood cells with hypotonic solution (eBioscience), the remaining cells were separated by centrifugation at 500 ×*g* at room temperature for 30 min over discontinuous Percoll (GE) gradients (55% [v/v], 65% [v/v], and 75% [v/v] in PBS). Neutrophils at the 75%-65% interface were removed and washed once with HBSS. The purity (> 90%) of the purified neutrophils was confirmed by flow cytometry with Gr-1 and CD11b antibodies (eBioscience).

### NET DNA Quantification

Neutrophils (1 ×10^5^) were seeded into 96 well plates in RPMI and stimulated with 200 nM phorbol 12-myristate 13-acetate (PMA), bacteria (MOI = 10), or left un-stimulated for 4 h. Then micrococcal nuclease (500 mU ml^-1^, Worthington) was added, and the samples were incubated for 10 min at 37°C. After addition of 5 mM EDTA, the released DNA was collected by centrifugation and measured with Picogreen double-stranded DNA kit (Invitrogen) according to the manufacturer’s recommendations.

### Bacteria killing assay

*S*. *aureus* cells were opsonized with 10% autologous serum at 37°C for 30 min, washed with of PBS, and suspended in RPMI. Neutrophils (2 ×10^5^) purified from C57BL/6 wild type or S100A9^-/-^ mice were added into 24-well tissue culture plate and allowed to adhere at 37°C for 1 h. *S*. *aureus* were added to neutrophils (bacteria: neutrophil = 10:1), and the plates were centrifuged at 300 ×*g* for 8 min at 4°C. Samples were incubated at 37°C for the indicated time period. To determine the cfu of surviving *S*. *aureus*, after lysis of neutrophils by treatment of 0.1% saponin on ice for 15 min, the resulting samples were diluted, spread on tryptic soy agar, and incubated at 37°C overnight. The percentages of killing were calculated by the following formula: [1-(CFU_neutrophils+_/CFU_neutrophils-_)] × 100.

### NET formation and immunofluorescence microscopy

Neutrophils (2 ×10^5^) from wild type or S100A9^-/-^ mice were seeded on 13 mm glass cover slips treated with 0.001% polylysine and allowed to settle and stimulated with 200 nM PMA for 4 h. Cells were fixed with 4% paraformaldehyde, treated with 0.1% Triton X-100, and blocked overnight in PBS containing 10% goat serum, 5% cold water fish gelatin, 1% BSA, and 0.05% Tween 20. CP was detected by treatment with S100A9 antibody (Novus Biological) and Cy3-conjugated secondary antibody (Invitrogen), while DNA was detected by DRAQ5 (Cell Signaling). Specimens were analyzed with Fluoview confocal microscope (Olympus).

### Animal experiment

C57BL/6 was purchased from the Jackson Laboratory (Bar Harbor, ME). CP deficient (C57BL/6 S100A9^-/-^) mice were acquired from the Skaar laboratory under the permission from University of Muenster. The CP-deficient mice were maintained in house as previously described [24]. *S*. *aureus* strains were grown in 3 ml of TSB at 37°C overnight with shaking (250 rpm). The next day the overnight culture was inoculated into fresh TSB (1:100 dilution) and incubated at 37°C for 2 h. *S*. *aureus* were collected by centrifugation, washed with of PBS, and suspended in sterile PBS to 4 OD_600_ (1 ×10^9^ cfu ml^-1^, for intraperitoneal injection) or 0.4 OD_600_ (1 ×10^8^ cfu ml^-1^ for retro-orbital injection). Sex matched eight-week-old C57BL/6 mice or S100A9^-/-^ mice were infected via intraperitoneal injection (2 ×10^8^ cfu or 1 ×10^9^ cfu) or retro-orbital injection (1 ×10^7^ cfu). To test the effects of proinflammatory cytokine-neutralizing antibodies on murine mortality, mice were injected once with the following antibodies at 2 h post infection: 100 μg of anti-TNFα (MP6-XT22), anti-IFNγ (R4-6A2), or anti-IL-1β (B122) Ab (Biolegend) or 150 μg of the antibody mixture (50 μg each). A rat IgG (whole molecule, Jackson ImmunoResearch) was used as an isotype control. The survival of the infected mice was monitored every hour for 24 h and then every 4 h for an additional 48 h (peritoneal infection model) or every 12 h for two weeks (retro-orbital infection model).

### Flow cytometry analysis

To determine the P1 promoter activity during peritoneal infection, mice were infected with USA300 (pCL-P1*gfp*). At desired time points, the peritoneum was washed with 2 ml HBSS using an 18 gauge needle and 5 ml syringe. The collected peritoneal fluid was subjected to centrifugation at 200 ×*g* for 5 min to separate the host cell-free fraction (supernatant) and the host cell-associate fraction (pellet). The supernatant was directly subjected to flow cytometry analysis. To release bacterial cells from the host cells in the pellet, the pellet was suspended in sterile water (pH 10.5) for 10 min at room temperature and subjected to vigorous vortex for 1 min. The GFP fluorescence from the P1 was detected in the FL-1 channel.

### RNA extraction and quantitative RT-PCR

The bacterial cells were harvested from peritoneal fluid as described above at 12 h post infection. Total RNA was isolated from harvested bacterial cells with a FastRNA pro blue kit (MP bio), and contaminating genomic DNA was eliminated with RNase-free DNase set (Qiagen). cDNA was generated from the purified RNA with SuperScript II RT (Invitrogen) and random primers (Applied Biosytems). PCR was performed on ABI PRISM 7000 sequence detection system (Applied Biosystems) using SYBR Green Master Mix (Applied Biosystems) and the primers listed in S10 Table. The transcript levels were calculated relative to that of 16S rRNA using the ΔCT method [63]: [relative expression = 2^-ΔCT^, where ΔCT = C_T_ (staphylococcal gene)-C_T_ (16S rRNA)]. The experiments were performed on RNA pooled from 3 mice per group and repeated 3 times.

### Cytokine ELISA

To measure cytokines produced from murine neutrophils, neutrophils (2 ×10^5^) from wild type or S100A9^-/-^ mice were infected with *S*. *aureus* for 2 h (MOI = 10) or left uninfected. Then cells were cultured in RPMI containing gentamicin (50 μg ml^-1^) for an additional 16 h, and the supernatants were collected. To measure cytokines produced in murine peritoneum, the peritoneal fluids were separated by centrifugation at 200 ×*g* for 10 min, and the supernatants were collected. Cytokines in the resulting supernatants were quantified with corresponding sandwich ELISA kits by following the manufacturer’s recommendations. The kits for TNF-α, IL-6, IL-1b, IL-17A/F and IFN-γ were purchased from eBioscience, while the MIP-2 (CXCL-2) ELISA kit was purchased from Sigma.

### Statistical analysis

Statistical analyses were performed by using the software Prism 5 (GraphPad). For the analyses of SaeQ expression, P1 promoter activity, and cytokine productions, two groups were compared by unpaired, two-tailed Student’s *t*-test. For the analyses of animal survival, however, Log-rank (Mantel-Cox) test was used. Differences were considered significant when p is smaller than 0.05.

## Supporting Information

S1 Fig Fe, Zn, and Cu do not affect the phosphotransferase and phosphatase activities of SaeS. MBP-SaeS was autophosphorylated with [γ-^32^P]-ATP; then the response regulator SaeR and various concentrations of FeSO_4_, ZnSO_4_, and CuSO_4_ were added. After 15 min incubation, the phosphorylated protein levels were monitored by SDS-PAGE and autoradiography. N, no SaeR.
(TIF)

S2 Fig The principal component analysis of the RNA-seq in Fig 4. Three biological replicates for each treatment (i.e., CP, Zn, and Zn-CP) were indicated with the same color. The close proximity of Zn-CP treated samples to the samples treated by Zn indicates a dominant Zn effect over CP.
(TIF)

S3 Fig GFP reporter plasmids for the analysis of P1 promoter activity. **(A)** Schematic diagrams of the reporter plasmids. The arrow indicates the transcription start site. P1, P1 promoter. **(B)** GFP expression from the negative control (pCL-*gfp*, gray) and the P1 reporter plasmid (pCL-P1*gfp*, white). *S*. *aureus* USA300 strains containing the plasmids were grown in RPMI medium for 16 h; then the expression of GFP was analyzed by flow cytometry. **(C)** Confirmation of the SaeRS repression by Fe and Zn. Cells were grown for 16 h in RPMI containing 400 μM CaCl_2_, 130 μM FeSO_4_, 130 μM MnSO_4_, or 400 μM ZnSO_4._ Then GFP expression was measured by flow cytometry.
(TIF)

S4 Fig CP does not affect the migration or bacterial killing of murine neutrophils. **(A)** Effect of CP on the migration of murine neutrophils. Mice were infected with *S*. *aureus* USA300 (2×10^8^ cfu) by intraperitoneal injection. At 2 h and 12 h post infection, peritoneal lavage was carried out, and the proportion of neutrophil (B220^-^Gr-1^+^CD11b^+^) was measured by flow cytometry (left panel). The absolute numbers of neutrophil, counted by hemocytometer, was also presented (right panel). Error bars indicate standard error of the mean. **(B**) The effect of CP on neutrophil extracellular traps (NET) formation. Neutrophils isolated from bone marrow of C57BL/6 (WT) or C57BL/6 S100A9^-/-^ (A9^-/-^) mice were stimulated with PMA (200 nM) for 4 h and stained with S100A9 antibody (green) and the DNA staining dye DRAQ5 (red). **(C)** NET-formation was stimulated by either PMA or *S*. *aureus* strain USA300 (MOI = 10) for 4 h; then the released DNA was quantified by Picogreen-dsDNA assay. **(D)** The effect of CP on bactericidal activity of murine neutrophils. Neutrophils were mixed with *S*. *aureus* strain USA300 (MOI = 10). At the time points indicated, neutrophils were lysed and spread on a tryptic soy agar. The data are from three pooled mice per genotype and represent three independent experiments.
(TIF)

S5 Fig The quantification results of Fig 6A. Statistical analysis was carried out by unpaired, two-tailed Student’s *t*-test.
(TIF)

S6 Fig CP increases murine mortality in the blood infection model. C57BL/6 (WT) or C57BL/6 S100A9^-/-^ (A9^-/-^) mice were infected with 1 × 10^7^ cfu of *S*. *aureus* USA300 (USA) or the *sae* deletion mutant (Δ*sae*) via retro-orbital injection. The infected mice were observed for 14 days. The significance of murine survival was assessed by Log-rank (Mantel-Cox) test.
(TIF)

S7 Fig Fur is not involved in the Fe-mediated repression of the SaeRS TCS. USA300 and USA300*fur*, a *fur* transposon mutant, carrying pCL-P1*gfp* reporter plasmid were grown in RPMI until 0.5 OD_600_. The cultures were divided into two, and 50 μM FeSO_4_ was added to one of the cultures. After 4.5 h incubation at 37°C, the resulting cultures (100 μl) were used to measure GFP expression with a microplate reader (Perkin-Elmer Envison 2103, 485 nm excitation, 538 nm emission). The fluorescence was normalized by OD_600_. WT, USA300; *fur*, USA300*fur*. AU, arbitrary unit.
(TIF)

S1 Table Genes up-regulated by 1.1 μM CP treatment (DOCX)

S2 Table Genes down-regulated by 1.1 μM CP treatment (DOCX)

S3 Table Genes up-regulated by 20 μM Zn treatment (DOCX)

S4 Table Genes down-regulated by 20 μM Zn treatment (DOCX)

S5 Table Genes up-regulated by 20 μM Zn/1.1 μM CP treatment (DOCX)

S6 Table Genes down-regulated by 20 μM Zn/1.1 μM CP treatment (DOCX)

S7 Table Genes up-regulated by CP in the presence of Zn (DOCX)

S8 Table Genes down-regulated by CP in the presence of Zn (DOCX)

S9 Table Bacterial strains and plasmids (DOCX)

S10 Table Primers used for quantitative RT-PCR (DOCX)
