# Supplementary figures and images for: Calprotectin Increases the Activity of the SaeRS Two Component System and Murine Mortality during Staphylococcus aureus Infections
Source: PLoS Pathog. 2015 Jul 6;11(7):e1005026. doi: 10.1371/journal.ppat.1005026 (PMC4492782; doi:10.1371/journal.ppat.1005026)

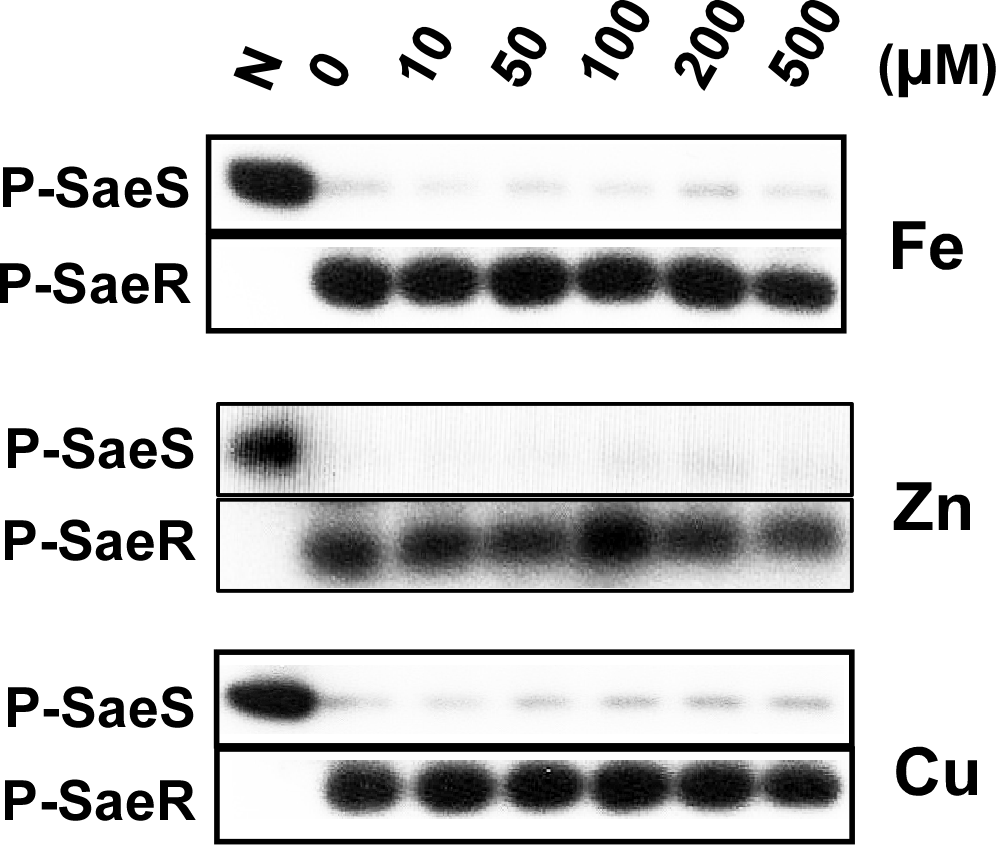

Supplement: S1 Fig — MBP-SaeS was autophosphorylated with [γ-32P]-ATP; then the response regulator SaeR and various concentrations of FeSO4, ZnSO4, and CuSO4 were added. After 15 min incubation, the phosphorylated protein levels were monitored by SDS-PAGE and autoradiography. N, no SaeR. (TIF) [file ppat.1005026.s001.tif]

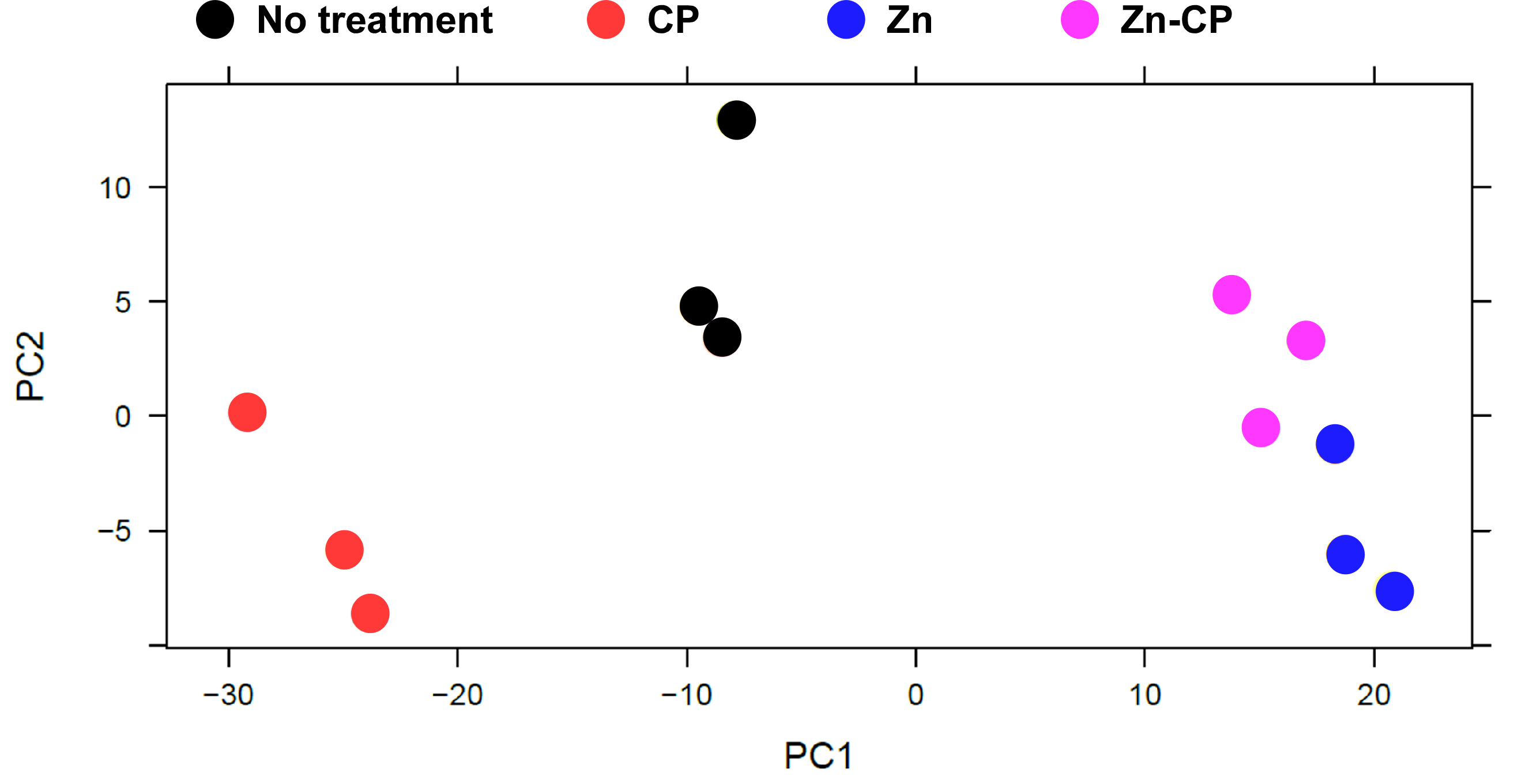

Supplement: S2 Fig — Three biological replicates for each treatment (i.e., CP, Zn, and Zn-CP) were indicated with the same color. The close proximity of Zn-CP treated samples to the samples treated by Zn indicates a dominant Zn effect over CP. (TIF) [file ppat.1005026.s002.tif]

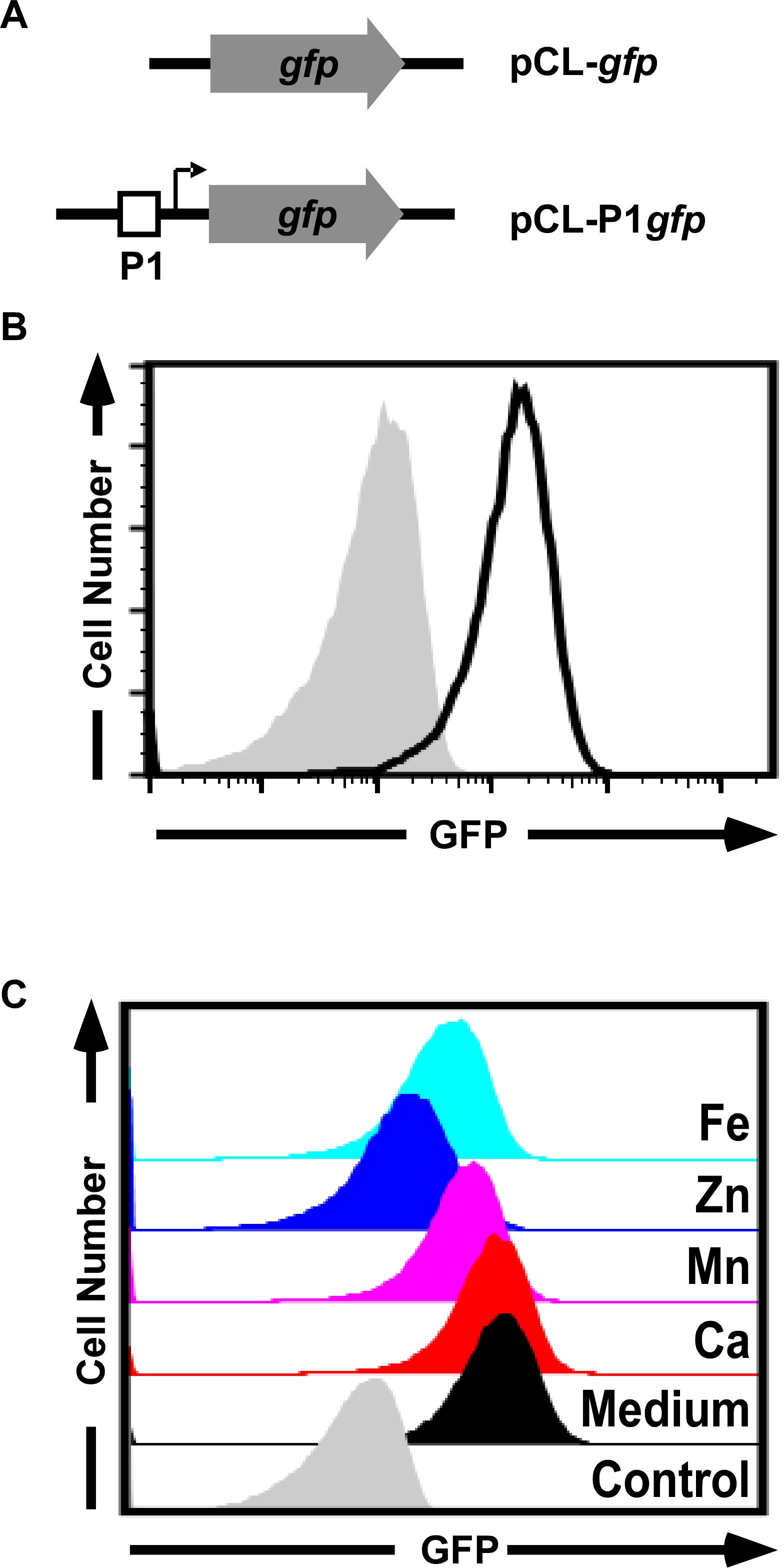

Supplement: S3 Fig — (A) Schematic diagrams of the reporter plasmids. The arrow indicates the transcription start site. P1, P1 promoter. (B) GFP expression from the negative control (pCL-gfp, gray) and the P1 reporter plasmid (pCL-P1gfp, white). S. aureus USA300 strains containing the plasmids were grown in RPMI medium for 16 h; then the expression of GFP was analyzed by flow cytometry. (C) Confirmation of the SaeRS repression by Fe and Zn. Cells were grown for 16 h in RPMI containing 400 μM CaCl2, 130 μM FeSO4, 130 μM MnSO4, or 400 μM ZnSO4. Then GFP expression was measured by flow cytometry. (TIF) [file ppat.1005026.s003.tif]

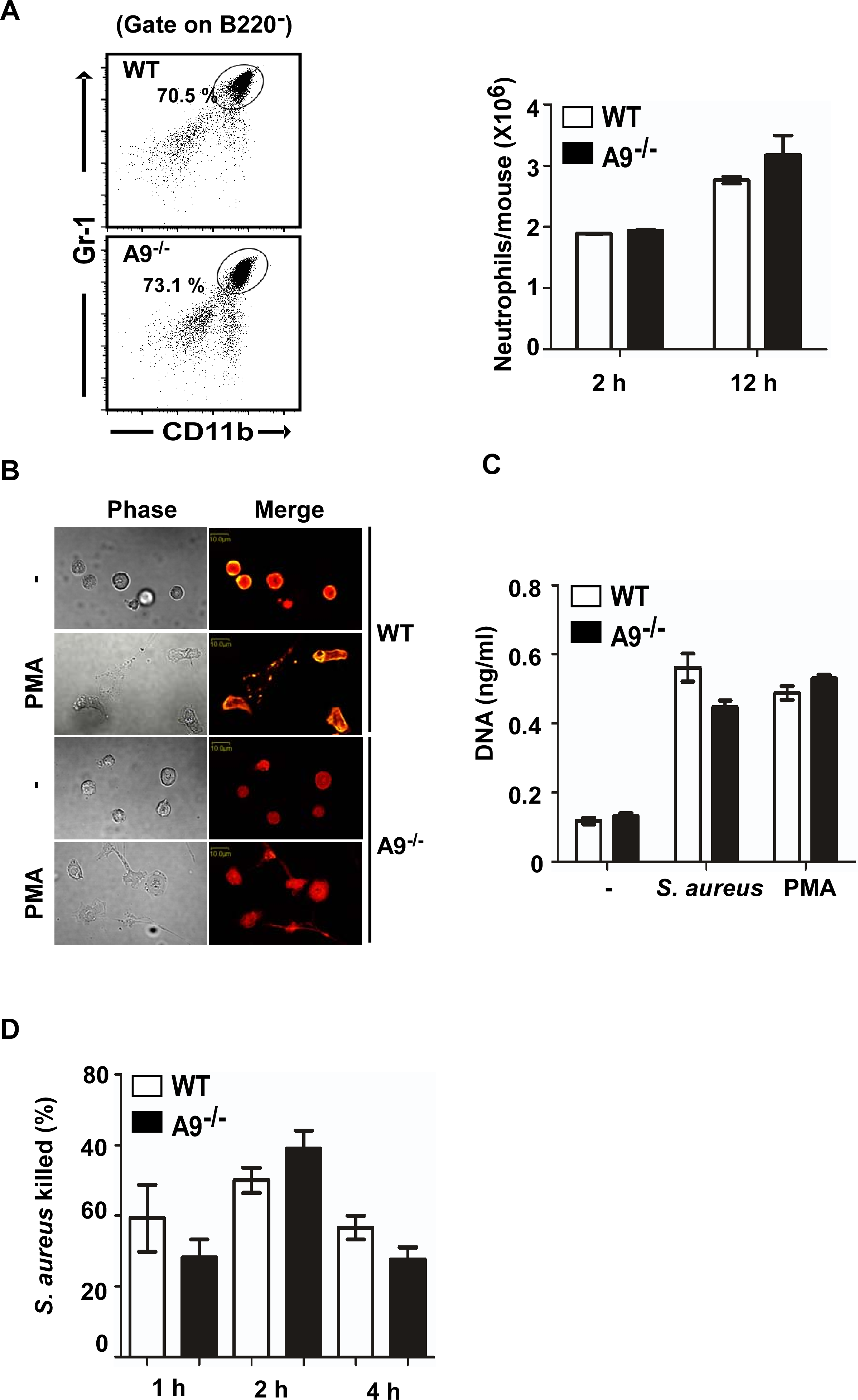

Supplement: S4 Fig — (A) Effect of CP on the migration of murine neutrophils. Mice were infected with S. aureus USA300 (2×10^8^ cfu) by intraperitoneal injection. At 2 h and 12 h post infection, peritoneal lavage was carried out, and the proportion of neutrophil (B220-Gr-1+CD11b+) was measured by flow cytometry (left panel). The absolute numbers of neutrophil, counted by hemocytometer, was also presented (right panel). Error bars indicate standard error of the mean. (B) The effect of CP on neutrophil extracellular traps (NET) formation. Neutrophils isolated from bone marrow of C57BL/6 (WT) or C57BL/6 S100A9-/- (A9-/-) mice were stimulated with PMA (200 nM) for 4 h and stained with S100A9 antibody (green) and the DNA staining dye DRAQ5 (red). (C) NET-formation was stimulated by either PMA or S. aureus strain USA300 (MOI = 10) for 4 h; then the released DNA was quantified by Picogreen-dsDNA assay. (D) The effect of CP on bactericidal activity of murine neutrophils. Neutrophils were mixed with S. aureus strain USA300 (MOI = 10). At the time points indicated, neutrophils were lysed and spread on a tryptic soy agar. The data are from three pooled mice per genotype and represent three independent experiments. (TIF) [file ppat.1005026.s004.tif]

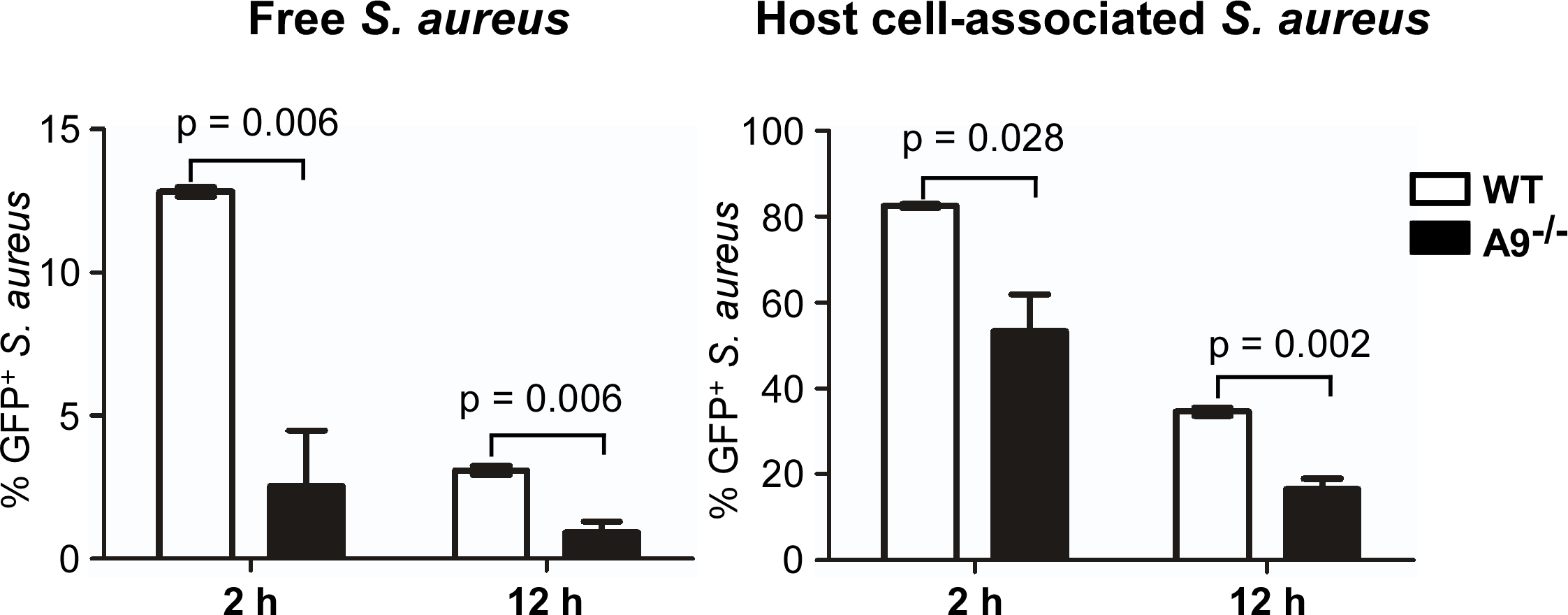

Supplement: S5 Fig — Statistical analysis was carried out by unpaired, two-tailed Student’s t-test. (TIF) [file ppat.1005026.s005.tif]

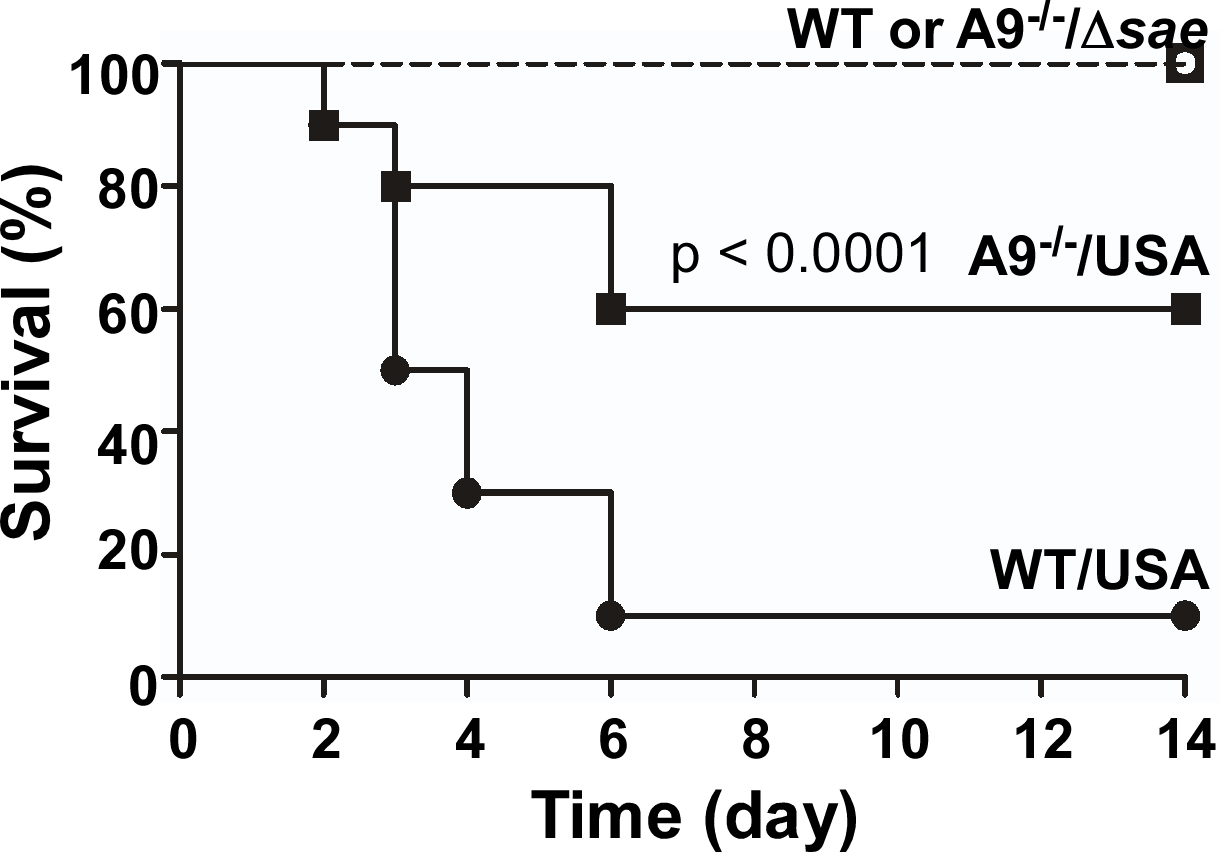

Supplement: S6 Fig — C57BL/6 (WT) or C57BL/6 S100A9-/- (A9-/-) mice were infected with 1 × 10^7^ cfu of S. aureus USA300 (USA) or the sae deletion mutant (Δsae) via retro-orbital injection. The infected mice were observed for 14 days. The significance of murine survival was assessed by Log-rank (Mantel-Cox) test. (TIF) [file ppat.1005026.s006.tif]

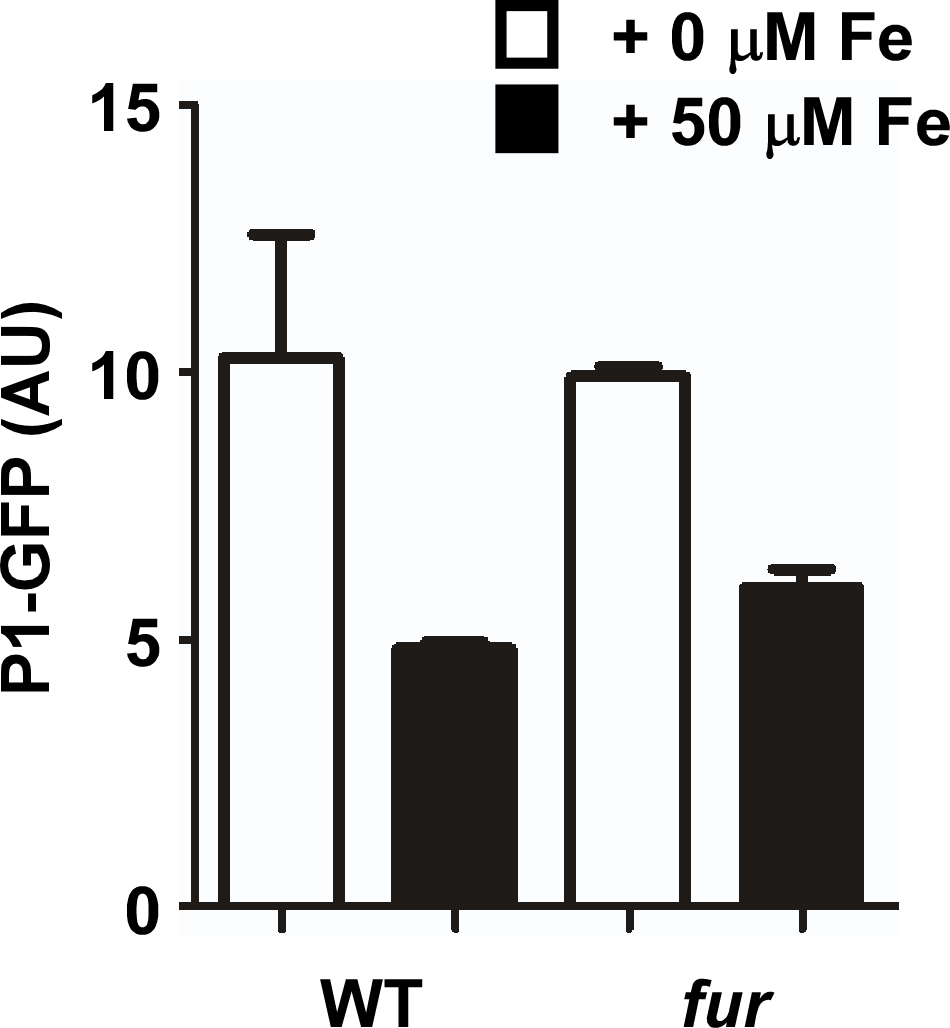

Supplement: S7 Fig — USA300 and USA300fur, a fur transposon mutant, carrying pCL-P1gfp reporter plasmid were grown in RPMI until 0.5 OD600. The cultures were divided into two, and 50 μM FeSO4 was added to one of the cultures. After 4.5 h incubation at 37°C, the resulting cultures (100 μl) were used to measure GFP expression with a microplate reader (Perkin-Elmer Envison 2103, 485 nm excitation, 538 nm emission). The fluorescence was normalized by OD600. WT, USA300; fur, USA300fur. AU, arbitrary unit. (TIF) [file ppat.1005026.s007.tif]
