# Supplementary material for: Calprotectin Increases the Activity of the SaeRS Two Component System and Murine Mortality during Staphylococcus aureus Infections
Source: PLoS Pathog. 2015 Jul 6;11(7):e1005026. doi: 10.1371/journal.ppat.1005026 (PMC4492782; doi:10.1371/journal.ppat.1005026)
Supplement: S1 Table — (DOCX) [file ppat.1005026.s008.docx]

**S1 Table. Genes up-regulated by 1.1 µM CP treatment**

| **ID** | **Name** | **Fold Change** | **p value** | **Gene product** |
| --- | --- | --- | --- | --- |
| SAUSA300_0072 |  | 2.84 | 0.0000 | hypothetical protein |
| SAUSA300_0073 |  | 2.74 | 0.0000 | peptide ABC transporter peptide-binding protein |
| SAUSA300_0074 | *opp-3B* | 2.65 | 0.0000 | oligopeptide permease channel-forming protein |
| SAUSA300_0075 | *opp-3C* | 2.69 | 0.0000 | oligopeptide permease channel-forming protein |
| SAUSA300_0076 |  | 2.75 | 0.0000 | ABC transporter ATP-binding protein |
| SAUSA300_0077 |  | 2.46 | 0.0003 | ABC transporter ATP-binding protein |
| SAUSA300_0113 |  | 1.87 | 0.0002 | immunoglobulin G binding protein A precursor |
| SAUSA300_0116 | *sirB* | 1.54 | 0.0236 | iron compound ABC transporter permease protein SirB |
| SAUSA300_0117 | *sirA* | 1.41 | 0.0024 | iron compound ABC transporter iron compound-binding protein SirA |
| SAUSA300_0118 |  | 2.60 | 0.0001 | pyridoxal-phosphate dependent enzyme superfamily |
| SAUSA300_0119 |  | 2.08 | 0.0111 | ornithine cyclodeaminase |
| SAUSA300_0120 | *sbnC* | 2.29 | 0.0000 | siderophore biosynthesis protein IucC family |
| SAUSA300_0121 |  | 1.99 | 0.0216 | putative drug transporter |
| SAUSA300_0122 |  | 1.95 | 0.0009 | siderophore biosynthesis protein IucA/IucC family |
| SAUSA300_0123 |  | 1.89 | 0.0051 | siderophore biosynthesis protein IucC family |
| SAUSA300_0124 |  | 1.91 | 0.0011 | HPCH/HPAI aldolase family protein |
| SAUSA300_0125 |  | 1.78 | 0.0012 | pyridoxal-dependent decarboxylase |
| SAUSA300_0126 |  | 1.66 | 0.0163 | conserved hypothetical protein |
| SAUSA300_0207 |  | 1.81 | 0.0061 | conserved hypothetical protein |
| SAUSA300_0270 | *lytM* | 1.42 | 0.0079 | peptidoglycan hydrolase |
| SAUSA300_0320 |  | 1.40 | 0.0055 | triacylglycerol lipase precursor |
| SAUSA300_0397 |  | 1.51 | 0.0141 | exotoxin |
| SAUSA300_0398 |  | 1.67 | 0.0029 | exotoxin |
| SAUSA300_0424 |  | 15.34 | 0.0000 | putative cobalamin synthesis protein |
| SAUSA300_0438 |  | 5.16 | 0.0000 | CHAP domain family |
| SAUSA300_0471 |  | 1.23 | 0.0415 | veg protein |
| SAUSA300_0472 | *ispE* | 1.40 | 0.0111 | 4-diphosphocytidyl-2C-methyl-D-erythritol kinase |
| SAUSA300_0634 | *fhuB* | 1.22 | 0.0466 | ferrichrome transport permease protein fhuB |
| SAUSA300_0651 |  | 2.71 | 0.0000 | CHAP domain family |
| SAUSA300_0681 |  | 1.84 | 0.0088 | conserved hypothetical protein |
| SAUSA300_0691 | *saeR* | 1.26 | 0.0447 | DNA-binding response regulator SaeR |
| SAUSA300_0693 | *saeP* | 1.41 | 0.0107 | SaeS regulatory protein SaeP |
| SAUSA300_0712 |  | 1.27 | 0.0484 | amino acid/peptide transporter |
| SAUSA300_0754 |  | 1.31 | 0.0329 | conserved hypothetical protein |
| SAUSA300_0776 | *nuc* | 1.66 | 0.0027 | thermonuclease precursor |
| SAUSA300_0796 |  | 1.55 | 0.0001 | ABC transporter ATP-binding protein |
| SAUSA300_0797 |  | 1.65 | 0.0001 | ABC transporter permease protein |
| SAUSA300_0798 |  | 1.86 | 0.0000 | ABC transporter substrate-binding protein |
| SAUSA300_0847 |  | 1.99 | 0.0005 | conserved hypothetical protein |
| SAUSA300_0933 |  | 1.64 | 0.0146 | conserved hypothetical protein |
| SAUSA300_0955 | *atl* | 2.58 | 0.0000 | autolysin |
| SAUSA300_1012 |  | 1.30 | 0.0131 | conserved hypothetical protein |
| SAUSA300_1028 |  | 2.09 | 0.0000 | iron transport associated domain protein |
| SAUSA300_1029 |  | 1.85 | 0.0000 | iron transport associated domain protein |
| SAUSA300_1030 |  | 1.65 | 0.0039 | iron transport associated domain protein |
| SAUSA300_1031 |  | 1.77 | 0.0005 | conserved hypothetical protein |
| SAUSA300_1032 |  | 1.95 | 0.0000 | putative iron compound ABC transporter iron compound-binding protein |
| SAUSA300_1033 |  | 1.62 | 0.0009 | iron heme permease |
| SAUSA300_1034 | *srtB* | 1.72 | 0.0004 | sortase B |
| SAUSA300_1035 |  | 1.73 | 0.0004 | conserved hypothetical protein |
| SAUSA300_1053 |  | 1.62 | 0.0113 | conserved hypothetical protein |
| SAUSA300_1061 |  | 1.79 | 0.0350 | putative exotoxin 3 |
| SAUSA300_1182 |  | 1.35 | 0.0418 | pyruvate ferredoxin oxidoreductase alpha subunit |
| SAUSA300_1233 | *rpmG* | 2.27 | 0.0000 | 50S ribosomal protein L33 |
| SAUSA300_1234 | *rpmN* | 12.46 | 0.0000 | 30S ribosomal protein S14-2 |
| SAUSA300_1286 |  | 1.59 | 0.0019 | aspartate kinase |
| SAUSA300_1287 | *asd* | 1.45 | 0.0034 | aspartate semialdehyde dehydrogenase |
| SAUSA300_1288 | *dapA* | 1.34 | 0.0231 | dihydrodipicolinate synthase |
| SAUSA300_1321 |  | 1.49 | 0.0011 | conserved hypothetical protein |
| SAUSA300_1322 |  | 1.60 | 0.0000 | conserved hypothetical protein |
| SAUSA300_1323 |  | 1.89 | 0.0000 | conserved hypothetical protein |
| SAUSA300_1535 | *rpsU* | 1.51 | 0.0001 | 30S ribosomal protein S21 |
| SAUSA300_1574 |  | 1.30 | 0.0240 | conserved hypothetical protein |
| SAUSA300_1712 | *ribH* | 1.61 | 0.0113 | riboflavin synthase beta subunit |
| SAUSA300_1713 | *ribBA* | 1.49 | 0.0159 | riboflavin biosynthesis protein |
| SAUSA300_1714 | *ribE* | 1.55 | 0.0067 | riboflavin synthase alpha subunit |
| SAUSA300_1715 | *ribD* | 1.68 | 0.0002 | riboflavin biosynthesis protein |
| SAUSA300_1730 | *metK* | 1.34 | 0.0192 | S-adenosylmethionine synthetase |
| SAUSA300_1920 | *chs* | 1.51 | 0.0032 | chemotaxis-inhibiting protein CHIPS |
| SAUSA300_2136 |  | 1.48 | 0.0112 | iron compound ABC transporter iron compound-binding protein |
| SAUSA300_2249 | *ssaA* | 1.96 | 0.0014 | secretory antigen precursor SsaA |
| SAUSA300_2253 | *ssaA* | 5.53 | 0.0000 | secretory antigen precursor SsaA |
| SAUSA300_2282 |  | 1.55 | 0.0051 | putative membrane protein |
| SAUSA300_2326 |  | 1.48 | 0.0010 | transcription regulatory protein |
| SAUSA300_2351 |  | 7.79 | 0.0000 | Zn-binding lipoprotein adcA-like protein |
| SAUSA300_2354 |  | 1.35 | 0.0089 | putative lipoprotein |
| SAUSA300_2364 | *sbi* | 1.72 | 0.0001 | IgG-binding protein SBI |
| SAUSA300_2406 |  | 3.36 | 0.0000 | putative transporter |
| SAUSA300_2407 |  | 3.11 | 0.0001 | oligopeptide ABC transporter ATP-binding protein |
| SAUSA300_2408 |  | 2.92 | 0.0005 | oligopeptide ABC transporter ATP-binding protein |
| SAUSA300_2409 |  | 2.83 | 0.0015 | oligopeptide ABC transporter permease protein |
| SAUSA300_2410 |  | 4.02 | 0.0000 | oligopeptide ABC transporter permease protein |
| SAUSA300_2411 | *opp* | 3.81 | 0.0000 | oligopeptide permease peptide-binding protein |
| SAUSA300_2412 |  | 3.78 | 0.0003 | conserved hypothetical protein |
| SAUSA300_2413 |  | 3.61 | 0.0004 | conserved hypothetical protein |
| SAUSA300_2414 |  | 3.52 | 0.0003 | conserved hypothetical protein |
| SAUSA300_2441 | *fnbA* | 1.31 | 0.0244 | fibronectin binding protein A |
| SAUSA300_2461 |  | 1.42 | 0.0317 | glyoxalase family protein |
| SAUSA300_2506 | *isaA* | 3.01 | 0.0000 | immunodominant staphylococcal antigen A precursor |
| SAUSA300_2519 |  | 7.21 | 0.0000 | putative cobalamin synthesis protein |
| SAUSA300_2520 |  | 10.04 | 0.0000 | transporter gate domain protein |
| SAUSA300_2521 |  | 15.46 | 0.0000 | conserved hypothetical protein |
| SAUSA300_2565 | *clfB* | 1.49 | 0.0391 | clumping factor B |
| SAUSA300_2572 | *aur* | 1.59 | 0.0115 | zinc metalloproteinase aureolysin |
| SAUSA300_2576 |  | 1.44 | 0.0045 | phosphotransferase system fructose-specific IIABC component |
| SAUSA300_2577 | *manA* | 1.43 | 0.0194 | mannose-6-phosphate isomerase class I |
