# Supplementary material for: Calprotectin Increases the Activity of the SaeRS Two Component System and Murine Mortality during Staphylococcus aureus Infections
Source: PLoS Pathog. 2015 Jul 6;11(7):e1005026. doi: 10.1371/journal.ppat.1005026 (PMC4492782; doi:10.1371/journal.ppat.1005026)
Supplement: S2 Table — (DOCX) [file ppat.1005026.s009.docx]

**S2 Table. Genes down-regulated by 1.1 µM CP treatment**

| **ID** | **Name** | **Fold Change** | **p value** | **Gene product** |
| --- | --- | --- | --- | --- |
| SAUSA300_0136 |  | 0.70 | 0.0034 | cell wall surface anchor family protein |
| SAUSA300_0138 | *deoD* | 0.55 | 0.0033 | purine nucleoside phosphorylase |
| SAUSA300_0139 |  | 0.60 | 0.0009 | putative tetracycline resistance protein |
| SAUSA300_0201 |  | 0.42 | 0.0005 | peptide ABC transporter permease protein |
| SAUSA300_0202 |  | 0.50 | 0.0113 | peptide ABC transporter permease protein |
| SAUSA300_0203 |  | 0.64 | 0.0406 | putative lipoprotein |
| SAUSA300_0229 |  | 0.60 | 0.0157 | putative acyl-CoA transferase FadX |
| SAUSA300_0319 |  | 0.30 | 0.0000 | putative membrane protein |
| SAUSA300_0355 |  | 0.64 | 0.0007 | acetyl-CoA acetyltransferase |
| SAUSA300_0367 | *ssb* | 0.76 | 0.0130 | single-strand binding protein |
| SAUSA300_0435 |  | 0.45 | 0.0040 | ABC transporter ATP-binding protein |
| SAUSA300_0436 |  | 0.47 | 0.0175 | ABC transporter permease protein |
| SAUSA300_0437 |  | 0.58 | 0.0063 | NLPA lipoprotein |
| SAUSA300_0479 |  | 0.78 | 0.0350 | ribosomal protein L25 Ctc-form |
| SAUSA300_0507 | *ctsR* | 0.66 | 0.0001 | transcriptional regulator CtsR |
| SAUSA300_0508 |  | 0.73 | 0.0011 | conserved hypothetical protein |
| SAUSA300_0509 |  | 0.74 | 0.0012 | ATP guanido phosphotransferase |
| SAUSA300_0510 | *clpC* | 0.74 | 0.0049 | endopeptidase |
| SAUSA300_0524 | *rplJ* | 0.77 | 0.0442 | ribosomal protein L10 |
| SAUSA300_0529 |  | 0.71 | 0.0088 | conserved hypothetical protein |
| SAUSA300_0530 | *rpsL* | 0.77 | 0.0320 | ribosomal protein S12 |
| SAUSA300_0531 |  | 0.75 | 0.0060 | 30S ribosomal protein S7 |
| SAUSA300_0532 | *fusA* | 0.73 | 0.0029 | translation elongation factor G |
| SAUSA300_0533 | *tuf* | 0.77 | 0.0466 | translation elongation factor Tu |
| SAUSA300_0536 |  | 0.67 | 0.0350 | Chaperone protein HchA |
| SAUSA300_0558 |  | 0.72 | 0.0264 | putative proline betaine transporter |
| SAUSA300_0573 | *mvaD* | 0.65 | 0.0007 | diphosphomevalonate decarboxylase |
| SAUSA300_0574 |  | 0.54 | 0.0000 | phosphomevalonate kinase |
| SAUSA300_0590 |  | 0.70 | 0.0070 | conserved hypothetical protein |
| SAUSA300_0636 |  | 0.68 | 0.0132 | dihydroxyacetone kinase DhaK subunit |
| SAUSA300_0638 |  | 0.69 | 0.0460 | dihydroxyacetone kinase phosphotransfer subunit |
| SAUSA300_0649 |  | 0.78 | 0.0480 | conserved hypothetical protein |
| SAUSA300_0742 | *uvrA* | 0.77 | 0.0141 | excinuclease ABC A subunit |
| SAUSA300_0764 | *rnr* | 0.76 | 0.0079 | ribonuclease R |
| SAUSA300_0765 | *smpB* | 0.76 | 0.0198 | SsrA-binding protein |
| SAUSA300_0770 |  | 0.61 | 0.0201 | conserved hypothetical protein |
| SAUSA300_0771 |  | 0.64 | 0.0131 | acetyltransferase GNAT family |
| SAUSA300_0782 |  | 0.58 | 0.0451 | conserved hypothetical protein |
| SAUSA300_0783 |  | 0.55 | 0.0018 | phosphoglycerate mutase family protein |
| SAUSA300_0844 |  | 0.72 | 0.0004 | conserved hypothetical protein |
| SAUSA300_0914 |  | 0.63 | 0.0054 | sodium:alanine symporter family protein |
| SAUSA300_0934 |  | 0.61 | 0.0208 | membrane protein |
| SAUSA300_0935 |  | 0.48 | 0.0156 | conserved hypothetical protein |
| SAUSA300_0944 | *menA* | 0.74 | 0.0246 | 1,4-dihydroxy-2-naphthoate octaprenyltransferase |
| SAUSA300_0976 | *purD* | 0.78 | 0.0105 | phosphoribosylamine--glycine ligase |
| SAUSA300_0994 | *pdhB* | 0.77 | 0.0344 | pyruvate dehydrogenase E1 component beta subunit |
| SAUSA300_0996 | *lpdA* | 0.78 | 0.0220 | dihydrolipoamide dehydrogenase |
| SAUSA300_1000 | *potB* | 0.67 | 0.0049 | spermidine/putrescine ABC transporter permease protein |
| SAUSA300_1001 | *potC* | 0.73 | 0.0300 | spermidine/putrescine ABC transporter permease protein |
| SAUSA300_1002 | *potD* | 0.72 | 0.0092 | spermidine/putrescine ABC transporter spermidine/putrescine-binding protein |
| SAUSA300_1077 | *murD* | 0.72 | 0.0031 | UDP-N-acetylmuramoylalanine--D-glutamate ligase |
| SAUSA300_1078 | *divIB* | 0.76 | 0.0178 | cell division protein |
| SAUSA300_1079 | *ftsA* | 0.80 | 0.0163 | cell division protein ftsA |
| SAUSA300_1125 | *acpP* | 0.77 | 0.0098 | acyl carrier protein |
| SAUSA300_1134 | *rplS* | 0.68 | 0.0057 | 50S ribosomal protein L19 |
| SAUSA300_1145 | *xerC* | 0.75 | 0.0108 | tyrosine recombinase xerC |
| SAUSA300_1146 | *hslV* | 0.75 | 0.0201 | ATP-dependent protease hslV |
| SAUSA300_1147 | *hslU* | 0.77 | 0.0113 | heat shock protein HslVU ATPase subunit HslU |
| SAUSA300_1241 |  | 0.71 | 0.0350 | conserved hypothetical protein |
| SAUSA300_1251 | *parC* | 0.76 | 0.0408 | DNA topoisomerase IV subunit A |
| SAUSA300_1252 |  | 0.78 | 0.0491 | amino acid carrier protein |
| SAUSA300_1257 | *msrR* | 0.76 | 0.0146 | peptide methionine sulfoxide reductase regulator MsrR |
| SAUSA300_1538 | *prmA* | 0.73 | 0.0048 | ribosomal protein L11 methyltransferase |
| SAUSA300_1539 | *dnaJ* | 0.69 | 0.0015 | chaperone protein DnaJ |
| SAUSA300_1540 | *dnaK* | 0.61 | 0.0000 | chaperone protein DnaK |
| SAUSA300_1541 | *grpE* | 0.39 | 0.0000 | co-chaperone GrpE |
| SAUSA300_1542 | *hrcA* | 0.33 | 0.0000 | heat-inducible transcription repressor HrcA |
| SAUSA300_1545 | *rpsT* | 0.67 | 0.0197 | 30S ribosomal protein S20 |
| SAUSA300_1595 | *tgt* | 0.77 | 0.0089 | queuine tRNA-ribosyltransferase |
| SAUSA300_1602 |  | 0.74 | 0.0197 | conserved hypothetical protein |
| SAUSA300_1603 | *rplU* | 0.76 | 0.0211 | 50S ribosomal protein L21 |
| SAUSA300_1610 | *folC* | 0.77 | 0.0142 | folylpolyglutamate synthase |
| SAUSA300_1627 | *infC* | 0.73 | 0.0213 | translation initiation factor IF-3 |
| SAUSA300_1666 | *rpsD* | 0.74 | 0.0353 | 30S ribosomal protein S4 |
| SAUSA300_1686 | *murC* | 0.73 | 0.0006 | UDP-N-acetylmuramate--alanine ligase |
| SAUSA300_1847 |  | 0.76 | 0.0201 | conserved hypothetical protein |
| SAUSA300_1865 | *vraR* | 0.74 | 0.0145 | DNA-binding response regulator |
| SAUSA300_1874 |  | 0.58 | 0.0003 | ferritins family protein |
| SAUSA300_1878 | *rumA* | 0.71 | 0.0025 | RNA methyltransferase TrmA family |
| SAUSA300_1910 |  | 0.64 | 0.0178 | putative membrane protein |
| SAUSA300_1988 |  | 0.73 | 0.0414 | delta-hemolysin precursor |
| SAUSA300_1996 | *amt* | 0.51 | 0.0015 | ammonium transporter |
| SAUSA300_2046 | *oxaA* | 0.76 | 0.0119 | membrane protein oxaA precursor |
| SAUSA300_2057 | *atpC* | 0.73 | 0.0142 | ATP synthase F1 epsilon subunit |
| SAUSA300_2058 | *atpD* | 0.75 | 0.0111 | ATP synthase F1 beta subunit |
| SAUSA300_2059 | *atpG* | 0.77 | 0.0235 | ATP synthase F1 gamma subunit |
| SAUSA300_2060 | *atpA* | 0.75 | 0.0173 | ATP synthase F1 alpha subunit |
| SAUSA300_2061 | *atpH* | 0.76 | 0.0161 | ATP synthase F1 delta subunit |
| SAUSA300_2062 | *atpF* | 0.76 | 0.0299 | ATP synthase F0 B subunit |
| SAUSA300_2063 | *atpE* | 0.75 | 0.0350 | ATP synthase F0 C subunit |
| SAUSA300_2064 | *atpB* | 0.77 | 0.0471 | ATP synthase F0 A subunit |
| SAUSA300_2078 | *murA* | 0.71 | 0.0057 | UDP-N-acetylglucosamine 1-carboxyvinyltransferase |
| SAUSA300_2081 | *pyrG* | 0.44 | 0.0000 | CTP synthase |
| SAUSA300_2084 | *coaA* | 0.72 | 0.0108 | pantothenate kinase |
| SAUSA300_2088 | *luxS* | 0.75 | 0.0317 | S-ribosylhomocysteinase |
| SAUSA300_2098 | *arsR* | 0.38 | 0.0036 | transcriptional repressor ArsR family |
| SAUSA300_2099 |  | 0.34 | 0.0000 | cation efflux family protein |
| SAUSA300_2104 | *glmS* | 0.43 | 0.0000 | glucosamine--fructose-6-phosphate aminotransferase isomerizing |
| SAUSA300_2145 |  | 0.57 | 0.0008 | glycine betaine transporter |
| SAUSA300_2236 |  | 0.57 | 0.0003 | conserved hypothetical protein |
| SAUSA300_2277 | *hutI* | 0.17 | 0.0000 | imidazolonepropionase |
| SAUSA300_2278 | *hutU* | 0.16 | 0.0000 | urocanate hydratase |
| SAUSA300_2317 |  | 0.72 | 0.0347 | putative zinc-binding dehydrogenase |
| SAUSA300_2468 |  | 0.52 | 0.0011 | acetyltransferase GNAT family |
| SAUSA300_2469 | *sdaAA* | 0.71 | 0.0092 | L-serine dehydratase iron-sulfur-dependent alpha subunit |
| SAUSA300_2470 | *sdaAB* | 0.65 | 0.0073 | L-serine dehydratase iron-sulfur-dependent beta subunit |
| SAUSA300_2471 |  | 0.69 | 0.0119 | perfringolysin O regulator protein |
| SAUSA300_2498 | *crtN* | 0.71 | 0.0329 | squalene synthase |
| SAUSA300_2500 |  | 0.67 | 0.0092 | glycosyl transferase |
| SAUSA300_2539 |  | 0.67 | 0.0113 | aminotransferase |
| SAUSA300_2545 | *betA* | 0.32 | 0.0000 | choline dehydrogenase |
| SAUSA300_2546 | *betB* | 0.24 | 0.0000 | glycine betaine aldehyde dehydrogenase |
| SAUSA300_2547 |  | 0.65 | 0.0013 | conserved hypothetical protein |
| SAUSA300_2549 | *bccT* | 0.48 | 0.0000 | choline/carnitine/betaine transporter BCCT family |
| SAUSA300_2558 |  | 0.72 | 0.0357 | sensor histidine kinase |
| SAUSA300_2559 |  | 0.61 | 0.0010 | DNA-binding response regulator |
| SAUSA300_2590 |  | 0.66 | 0.0116 | conserved hypothetical protein |
| SAUSA300_2614 |  | 0.67 | 0.0142 | putative lipoprotein |
| SAUSA300_2615 |  | 0.63 | 0.0460 | conserved hypothetical protein |
| SAUSA300_2616 |  | 0.57 | 0.0027 | cobalt transport family protein |
| SAUSA300_2617 |  | 0.53 | 0.0000 | putative cobalt ABC transporter ATP-binding protein |
| SAUSA300_2618 |  | 0.48 | 0.0000 | conserved hypothetical protein |
| SAUSA300_2619 |  | 0.49 | 0.0000 | conserved hypothetical protein |
| SAUSA300_2626 |  | 0.59 | 0.0132 | conserved hypothetical protein |
| SAUSA300_2644 | *gidB* | 0.75 | 0.0329 | glucose-inhibited division protein B |
