# Supplementary material for: Calprotectin Increases the Activity of the SaeRS Two Component System and Murine Mortality during Staphylococcus aureus Infections
Source: PLoS Pathog. 2015 Jul 6;11(7):e1005026. doi: 10.1371/journal.ppat.1005026 (PMC4492782; doi:10.1371/journal.ppat.1005026)
Supplement: S5 Table — (DOCX) [file ppat.1005026.s012.docx]

**S5 Table. Genes up-regulated by 20 µM Zn/1.1 µM CP treatment**

| **ID** | **Name** | **Fold Change** | **p value** | **Gene product** |
| --- | --- | --- | --- | --- |
| SAUSA300_0055 |  | 1.94 | 0.0000 | alcohol dehydrogenase zinc-containing |
| SAUSA300_0067 |  | 1.59 | 0.0018 | universal stress protein family |
| SAUSA300_0078 | *copA* | 2.26 | 0.0000 | ATPase copper transport |
| SAUSA300_0079 |  | 1.96 | 0.0000 | putative lipoprotein |
| SAUSA300_0105 |  | 1.44 | 0.0487 | peptidase M20/M25/M40 family |
| SAUSA300_0107 |  | 1.47 | 0.0252 | Na/Pi cotransporter family protein |
| SAUSA300_0108 |  | 2.07 | 0.0000 | antigen 67 kDa |
| SAUSA300_0127 |  | 1.81 | 0.0024 | conserved hypothetical protein |
| SAUSA300_0128 |  | 1.49 | 0.0412 | conserved hypothetical protein |
| SAUSA300_0183 |  | 1.59 | 0.0018 | conserved hypothetical protein |
| SAUSA300_0218 |  | 1.35 | 0.0113 | sensor histidine kinase family protein |
| SAUSA300_0228 | *fadE* | 2.02 | 0.0006 | acyl-CoA synthetase FadE |
| SAUSA300_0229 |  | 1.94 | 0.0002 | putative acyl-CoA transferase FadX |
| SAUSA300_0231 |  | 2.25 | 0.0000 | ABC transporter substrate-binding protein |
| SAUSA300_0232 |  | 5.49 | 0.0000 | conserved hypothetical protein |
| SAUSA300_0233 |  | 4.08 | 0.0001 | conserved hypothetical protein |
| SAUSA300_0234 |  | 4.88 | 0.0000 | putative flavohemoprotein |
| SAUSA300_0245 |  | 1.44 | 0.0004 | 2-C-methyl-D-erythritol 4-phosphate cytidylyltransferase |
| SAUSA300_0253 | *scdA* | 3.41 | 0.0000 | ScdA protein |
| SAUSA300_0271 |  | 1.98 | 0.0005 | ABC transporter ATP-binding protein |
| SAUSA300_0272 |  | 1.88 | 0.0051 | conserved hypothetical protein |
| SAUSA300_0273 |  | 2.80 | 0.0008 | putative membrane protein |
| SAUSA300_0274 |  | 2.53 | 0.0000 | conserved hypothetical protein |
| SAUSA300_0277 |  | 1.83 | 0.0000 | putative staphyloxanthin biosynthesis protein |
| SAUSA300_0278 |  | 1.76 | 0.0000 | conserved hypothetical protein |
| SAUSA300_0279 |  | 1.71 | 0.0002 | putative membrane protein |
| SAUSA300_0282 |  | 1.84 | 0.0041 | conserved hypothetical protein |
| SAUSA300_0283 |  | 1.39 | 0.0111 | essC protein |
| SAUSA300_0300 |  | 1.70 | 0.0494 | conserved hypothetical protein |
| SAUSA300_0304 |  | 1.56 | 0.0249 | conserved hypothetical protein |
| SAUSA300_0307 |  | 1.49 | 0.0003 | 5'-nucleotidase lipoprotein e(P4) family |
| SAUSA300_0308 |  | 1.85 | 0.0000 | ABC transporter permease protein |
| SAUSA300_0309 |  | 1.83 | 0.0000 | ABC transporter ATP-binding protein |
| SAUSA300_0310 | *pfoR* | 1.34 | 0.0037 | perfringolysin O regulator protein |
| SAUSA300_0329 |  | 1.33 | 0.0463 | putative oxidoreductase |
| SAUSA300_0385 |  | 1.32 | 0.0308 | conserved hypothetical protein |
| SAUSA300_0394 |  | 1.55 | 0.0047 | FAD/NAD(P)-binding Rossmann fold Superfamily |
| SAUSA300_0413 |  | 2.27 | 0.0069 | staphylococcal tandem lipoprotein |
| SAUSA300_0419 |  | 1.88 | 0.0000 | staphylococcal tandem lipoprotein |
| SAUSA300_0420 |  | 1.91 | 0.0000 | conserved hypothetical protein |
| SAUSA300_0421 |  | 1.60 | 0.0002 | conserved hypothetical protein |
| SAUSA300_0422 |  | 1.43 | 0.0320 | conserved hypothetical protein |
| SAUSA300_0461 | *holB* | 1.37 | 0.0306 | DNA polymerase III delta subunit |
| SAUSA300_0466 |  | 1.39 | 0.0272 | conserved hypothetical protein |
| SAUSA300_0470 | *ksgA* | 1.37 | 0.0063 | dimethyladenosine transferase |
| SAUSA300_0506 | *nupC* | 1.49 | 0.0000 | pyrimidine nucleoside transport protein |
| SAUSA300_0507 | *ctsR* | 1.68 | 0.0000 | transcriptional regulator CtsR |
| SAUSA300_0508 |  | 1.62 | 0.0000 | conserved hypothetical protein |
| SAUSA300_0509 |  | 1.66 | 0.0000 | ATP guanido phosphotransferase |
| SAUSA300_0510 | *clpC* | 1.70 | 0.0000 | endopeptidase |
| SAUSA300_0513 | *gltX* | 1.40 | 0.0026 | glutamyl-tRNA synthetase |
| SAUSA300_0514 | *cysE* | 1.46 | 0.0014 | serine acetyltransferase |
| SAUSA300_0515 | *cysS* | 1.47 | 0.0001 | cysteinyl-tRNA synthetase |
| SAUSA300_0516 | *cysS* | 1.44 | 0.0022 | conserved hypothetical protein |
| SAUSA300_0517 |  | 1.41 | 0.0033 | RNA methyltransferase TrmH family group 3 |
| SAUSA300_0518 |  | 1.49 | 0.0009 | conserved hypothetical protein |
| SAUSA300_0519 |  | 1.54 | 0.0022 | conserved hypothetical protein |
| SAUSA300_0521 | *nusG* | 1.25 | 0.0079 | transcription termination/antitermination factor NusG |
| SAUSA300_0526 |  | 1.29 | 0.0471 | Methyltransferase small domain |
| SAUSA300_0530 | *rpsL* | 1.29 | 0.0144 | ribosomal protein S12 |
| SAUSA300_0531 |  | 1.27 | 0.0125 | 30S ribosomal protein S7 |
| SAUSA300_0532 | *fusA* | 1.23 | 0.0358 | translation elongation factor G |
| SAUSA300_0545 |  | 1.30 | 0.0349 | Flavodoxin-like fold |
| SAUSA300_0547 | *sdrD* | 1.41 | 0.0050 | sdrD protein |
| SAUSA300_0550 |  | 1.50 | 0.0000 | glycosyl transferase group 1 family protein |
| SAUSA300_0558 |  | 1.55 | 0.0002 | putative proline/betaine transporter |
| SAUSA300_0563 | *ung* | 1.30 | 0.0437 | uracil-DNA glycosylase |
| SAUSA300_0565 |  | 1.32 | 0.0271 | conserved hypothetical protein |
| SAUSA300_0573 | *mvaD* | 1.51 | 0.0003 | diphosphomevalonate decarboxylase |
| SAUSA300_0574 |  | 1.48 | 0.0003 | phosphomevalonate kinase |
| SAUSA300_0579 |  | 1.76 | 0.0000 | conserved hypothetical protein |
| SAUSA300_0580 |  | 1.66 | 0.0141 | conserved hypothetical protein |
| SAUSA300_0581 |  | 1.47 | 0.0191 | conserved hypothetical protein |
| SAUSA300_0589 |  | 1.74 | 0.0000 | aldo/keto reductase family protein |
| SAUSA300_0590 |  | 1.42 | 0.0017 | conserved hypothetical protein |
| SAUSA300_0594 | *adh* | 1.56 | 0.0028 | alcohol dehydrogenase |
| SAUSA300_0597 |  | 1.82 | 0.0066 | putative endonuclease III |
| SAUSA300_0604 |  | 1.41 | 0.0214 | hydrolase alpha/beta hydrolase fold family |
| SAUSA300_0610 |  | 1.45 | 0.0292 | putative Na+/H+ antiporter MnhA component |
| SAUSA300_0626 | *tagB* | 1.33 | 0.0119 | teichoic acid biosynthesis protein B |
| SAUSA300_0627 | *tagX* | 1.40 | 0.0055 | teichoic acid biosynthesis protein X |
| SAUSA300_0628 |  | 1.42 | 0.0058 | teichoic acid biosynthesis protein D |
| SAUSA300_0636 |  | 1.62 | 0.0002 | dihydroxyacetone kinase DhaK subunit |
| SAUSA300_0637 |  | 1.63 | 0.0007 | dihydroxyacetone kinase DhaL subunit |
| SAUSA300_0638 |  | 1.54 | 0.0017 | dihydroxyacetone kinase phosphotransfer subunit |
| SAUSA300_0639 |  | 1.26 | 0.0144 | conserved hypothetical protein |
| SAUSA300_0643 |  | 1.40 | 0.0197 | acetyltransferase GNAT family |
| SAUSA300_0647 |  | 1.56 | 0.0493 | ABC transporter ATP-binding protein |
| SAUSA300_0658 |  | 1.26 | 0.0413 | transcriptional regulator LysR family |
| SAUSA300_0664 |  | 1.32 | 0.0295 | conserved hypothetical protein |
| SAUSA300_0668 |  | 1.36 | 0.0223 | conserved hypothetical protein |
| SAUSA300_0670 |  | 1.29 | 0.0129 | ABC transporter ATP-binding protein MsbA family |
| SAUSA300_0671 |  | 1.25 | 0.0459 | ABC transporter ATP-binding protein MsbA family |
| SAUSA300_0676 |  | 1.53 | 0.0003 | anion transporter family protein |
| SAUSA300_0677 |  | 1.38 | 0.0133 | putative deoxyribodipyrimidine photolyase |
| SAUSA300_0680 | *norA* | 1.41 | 0.0053 | multi drug resistance protein |
| SAUSA300_0682 | *ybaK* | 1.52 | 0.0143 | ybaK/ebsC protein |
| SAUSA300_0683 |  | 1.56 | 0.0258 | transcriptional regulator DeoR family |
| SAUSA300_0684 | *fruB* | 1.48 | 0.0030 | fructose 1-phosphate kinase |
| SAUSA300_0686 | *nagA* | 1.29 | 0.0393 | N-acetylglucosamine-6-phosphate deacetylase |
| SAUSA300_0688 |  | 1.49 | 0.0057 | oxidoreductase aldo/keto reductase family |
| SAUSA300_0726 |  | 1.50 | 0.0000 | glycerate kinase family protein |
| SAUSA300_0747 | *trxB* | 1.37 | 0.0027 | thioredoxin-disulfide reductase |
| SAUSA300_0752 | *clpP* | 1.47 | 0.0001 | ATP-dependent Clp protease proteolytic subunit ClpP |
| SAUSA300_0755 |  | 1.47 | 0.0036 | glycolytic operon regulator |
| SAUSA300_0756 | *gap* | 1.69 | 0.0031 | glyceraldehyde-3-phosphate dehydrogenase type I |
| SAUSA300_0757 | *pgk* | 2.26 | 0.0040 | phosphoglycerate kinase |
| SAUSA300_0758 | *tpiA* | 1.96 | 0.0289 | triosephosphate isomerase |
| SAUSA300_0760 | *eno* | 1.42 | 0.0080 | phosphopyruvate hydratase |
| SAUSA300_0764 | *rnr* | 1.23 | 0.0277 | ribonuclease R |
| SAUSA300_0765 | *smpB* | 1.24 | 0.0430 | SsrA-binding protein |
| SAUSA300_0768 |  | 1.51 | 0.0068 | conserved hypothetical protein |
| SAUSA300_0769 |  | 2.29 | 0.0000 | putative lipoprotein |
| SAUSA300_0772 | *clfA* | 1.89 | 0.0000 | clumping factor A |
| SAUSA300_0786 |  | 1.47 | 0.0367 | OsmC/Ohr family protein |
| SAUSA300_0793 |  | 1.36 | 0.0292 | conserved hypothetical protein |
| SAUSA300_0817 |  | 1.54 | 0.0007 | putative membrane protein |
| SAUSA300_0818 | *sufC* | 1.28 | 0.0039 | FeS assembly ATPase SufC |
| SAUSA300_0819 | *sufD* | 1.30 | 0.0144 | FeS assembly protein SufD |
| SAUSA300_0820 | *sufS* | 1.24 | 0.0461 | cysteine desulfurases SufS subfamily subfamily |
| SAUSA300_0826 |  | 1.29 | 0.0162 | conserved hypothetical protein |
| SAUSA300_0832 |  | 1.30 | 0.0099 | conserved hypothetical protein |
| SAUSA300_0833 |  | 1.35 | 0.0063 | conserved hypothetical protein |
| SAUSA300_0834 |  | 1.22 | 0.0404 | D-isomer specific 2-hydroxyacid dehydrogenase |
| SAUSA300_0844 |  | 1.27 | 0.0078 | conserved hypothetical protein |
| SAUSA300_0859 |  | 1.39 | 0.0074 | NADH-dependent flavin oxidoreductase |
| SAUSA300_0860 | *rocD* | 1.51 | 0.0037 | Ornithine aminotransferase |
| SAUSA300_0869 | *rexB* | 1.40 | 0.0005 | exonuclease RexB |
| SAUSA300_0870 | *rexA* | 1.30 | 0.0089 | exonuclease RexA |
| SAUSA300_0877 | *clpB* | 2.11 | 0.0000 | Chaperone clpB |
| SAUSA300_0883 |  | 1.74 | 0.0000 | putative surface protein |
| SAUSA300_0885 | *fabH* | 1.40 | 0.0017 | 3-oxoacyl-(acyl-carrier-protein) synthase III |
| SAUSA300_0886 | *fabF* | 1.59 | 0.0000 | 3-oxoacyl-(acyl-carrier-protein) synthase II |
| SAUSA300_0887 | *oppB* | 1.59 | 0.0110 | oligopeptide ABC transporter permease protein |
| SAUSA300_0888 | *oppC* | 1.74 | 0.0027 | oligopeptide ABC transporter permease protein |
| SAUSA300_0889 | *oppD* | 1.75 | 0.0009 | oligopeptide ABC transporter ATP-binding protein |
| SAUSA300_0890 | *oppF* | 1.71 | 0.0019 | oligopeptide ABC transporter ATP-binding protein |
| SAUSA300_0891 | *oppA* | 1.58 | 0.0035 | oligopeptide ABC transporter substrate-binding protein |
| SAUSA300_0892 | *oppA* | 1.66 | 0.0138 | oligopeptide ABC transporter oligopeptide-binding protein |
| SAUSA300_0906 |  | 1.46 | 0.0005 | conserved hypothetical protein |
| SAUSA300_0907 |  | 1.33 | 0.0035 | GTP pyrophosphokinase |
| SAUSA300_0908 |  | 1.33 | 0.0003 | NAD(+)/NADH kinase |
| SAUSA300_0909 |  | 1.50 | 0.0004 | pseudouridine synthases RluA subfamily |
| SAUSA300_0910 | *mgtE* | 1.47 | 0.0001 | magnesium transporter |
| SAUSA300_0911 |  | 1.30 | 0.0039 | transporter monovalent cation:proton antiporter-2 (CPA2) family protein |
| SAUSA300_0912 |  | 1.24 | 0.0221 | trans-2-enoyl-ACP reductase |
| SAUSA300_0914 |  | 1.47 | 0.0094 | sodium:alanine symporter family protein |
| SAUSA300_0916 |  | 1.41 | 0.0026 | conserved hypothetical protein |
| SAUSA300_0923 | *htrA* | 1.55 | 0.0017 | serine protease |
| SAUSA300_0925 |  | 1.40 | 0.0009 | 5' nucleotidase family protein |
| SAUSA300_0930 |  | 1.43 | 0.0121 | lipoate-protein ligase A family protein |
| SAUSA300_0940 |  | 2.14 | 0.0000 | conserved hypothetical protein |
| SAUSA300_0947 | *menD* | 1.30 | 0.0110 | hydrolase alpha/beta hydrolase fold family |
| SAUSA300_0960 | *qoxD* | 1.49 | 0.0031 | quinol oxidase subunit IV |
| SAUSA300_0961 | *qoxC* | 1.71 | 0.0000 | quinol oxidase subunit III |
| SAUSA300_0962 | *qoxB* | 1.76 | 0.0000 | quinol oxidase subunit I |
| SAUSA300_0963 | *qoxA* | 1.77 | 0.0000 | quinol oxidase subunit II |
| SAUSA300_0966 | *purE* | 1.64 | 0.0184 | phosphoribosylaminoimidazole carboxylase catalytic subunit |
| SAUSA300_0967 | *purK* | 1.68 | 0.0069 | phosphoribosylaminoimidazole carboxylase ATPase subunit |
| SAUSA300_0968 | *purC* | 1.69 | 0.0043 | phosphoribosylaminoimidazole-succinocarboxamide synthase |
| SAUSA300_0969 | *purS* | 1.60 | 0.0071 | phosphoribosylformylglycinamidine synthase |
| SAUSA300_0970 | *purQ* | 1.75 | 0.0014 | phosphoribosylformylglycinamidine synthase I |
| SAUSA300_0971 | *purL* | 1.71 | 0.0003 | phosphoribosylformylglycinamidine synthase II |
| SAUSA300_0972 | *purF* | 1.61 | 0.0002 | amidophosphoribosyltransferase |
| SAUSA300_0973 | *purM* | 1.51 | 0.0017 | phosphoribosylformylglycinamidine cyclo-ligase |
| SAUSA300_0974 | *purN* | 1.44 | 0.0027 | phosphoribosylglycinamide formyltransferase |
| SAUSA300_0975 | *purH* | 1.36 | 0.0014 | bifunctional purine biosynthesis protein |
| SAUSA300_0976 | *purD* | 1.24 | 0.0138 | phosphoribosylamine--glycine ligase |
| SAUSA300_0981 |  | 1.25 | 0.0500 | conserved hypothetical protein |
| SAUSA300_0982 |  | 1.67 | 0.0000 | conserved hypothetical protein |
| SAUSA300_0983 | *ptsH* | 1.27 | 0.0065 | phosphocarrier protein HPr |
| SAUSA300_0984 | *ptsI* | 1.23 | 0.0203 | phosphoenolpyruvate-protein phosphotransferase |
| SAUSA300_0986 |  | 2.36 | 0.0000 | cytochrome D ubiquinol oxidase subunit I |
| SAUSA300_0987 |  | 2.46 | 0.0000 | cytochrome D ubiquinol oxidase subunit II |
| SAUSA300_0993 | *pdhA* | 1.47 | 0.0000 | pyruvate dehydrogenase E1 component alpha subunit |
| SAUSA300_0994 | *pdhB* | 1.41 | 0.0007 | pyruvate dehydrogenase E1 component beta subunit |
| SAUSA300_0995 |  | 1.38 | 0.0004 | dihydrolipoamide acetyltransferase |
| SAUSA300_0996 | *lpdA* | 1.29 | 0.0051 | dihydrolipoamide dehydrogenase |
| SAUSA300_1000 | *potB* | 1.53 | 0.0003 | spermidine/putrescine ABC transporter permease protein |
| SAUSA300_1001 | *potC* | 1.52 | 0.0003 | spermidine/putrescine ABC transporter permease protein |
| SAUSA300_1002 | *potD* | 1.53 | 0.0001 | spermidine/putrescine ABC transporter spermidine/putrescine-binding protein |
| SAUSA300_1007 |  | 1.21 | 0.0427 | inositol monophosphatase family protein |
| SAUSA300_1009 | *typA* | 1.48 | 0.0233 | GTP-binding protein |
| SAUSA300_1015 | *ctaA* | 1.74 | 0.0000 | cytochrome oxidase assembly protein |
| SAUSA300_1016 | *cyoE* | 2.25 | 0.0000 | protoheme IX farnesyltransferase |
| SAUSA300_1017 |  | 2.34 | 0.0000 | conserved hypothetical protein |
| SAUSA300_1043 | *mutS2* | 1.40 | 0.0001 | DNA mismatch repair MutS2 protein |
| SAUSA300_1045 | *uvrC* | 1.33 | 0.0252 | excinuclease ABC C subunit |
| SAUSA300_1065 |  | 1.49 | 0.0200 | exfoliative toxin A |
| SAUSA300_1072 | *mraZ* | 2.85 | 0.0000 | protein mraZ |
| SAUSA300_1073 | *mraW* | 2.69 | 0.0000 | S-adenosyl-methyltransferase MraW |
| SAUSA300_1074 | *ftsL* | 1.89 | 0.0000 | cell division protein |
| SAUSA300_1075 | *pbpA* | 2.32 | 0.0000 | penicillin-binding protein 1 |
| SAUSA300_1076 | *mraY* | 2.36 | 0.0000 | phospho-N-acetylmuramoyl-pentapeptide-transferase |
| SAUSA300_1077 | *murD* | 2.52 | 0.0000 | UDP-N-acetylmuramoylalanine--D-glutamate ligase |
| SAUSA300_1078 | *divIB* | 2.13 | 0.0000 | cell division protein |
| SAUSA300_1079 | *ftsA* | 1.45 | 0.0000 | cell division protein ftsA |
| SAUSA300_1080 | *ftsZ* | 1.41 | 0.0002 | cell division protein ftsZ |
| SAUSA300_1100 |  | 1.23 | 0.0349 | conserved hypothetical protein |
| SAUSA300_1104 | *coaBC* | 1.32 | 0.0092 | phosphopantothenoylcysteine decarboxylase/phosphopantothenate--cysteine ligase |
| SAUSA300_1106 |  | 1.86 | 0.0000 | putative lipoprotein |
| SAUSA300_1109 | *fmt* | 1.43 | 0.0009 | methionyl-tRNA formyltransferase |
| SAUSA300_1110 | *sun* | 1.40 | 0.0012 | ribosomal RNA small subunit methyltransferase B |
| SAUSA300_1111 |  | 1.42 | 0.0007 | conserved hypothetical protein |
| SAUSA300_1112 |  | 1.41 | 0.0009 | protein phosphatase 2C domain protein |
| SAUSA300_1113 | *pknB* | 1.33 | 0.0024 | protein kinase |
| SAUSA300_1119 |  | 1.42 | 0.0086 | conserved hypothetical protein |
| SAUSA300_1143 | *topA* | 1.37 | 0.0129 | DNA topoisomerase I |
| SAUSA300_1144 | *gid* | 1.40 | 0.0041 | glucose inhibited division protein |
| SAUSA300_1145 | *xerC* | 1.46 | 0.0000 | tyrosine recombinase xerC |
| SAUSA300_1146 | *hslV* | 1.43 | 0.0004 | ATP-dependent protease hslV |
| SAUSA300_1147 | *hslU* | 1.29 | 0.0047 | heat shock protein HslVU ATPase subunit HslU |
| SAUSA300_1148 | *codY* | 1.35 | 0.0006 | GTP-sensing transcriptional pleiotropic repressor CodY |
| SAUSA300_1160 |  | 1.33 | 0.0208 | conserved hypothetical protein |
| SAUSA300_1161 |  | 1.26 | 0.0215 | ribosomal protein L7Ae |
| SAUSA300_1162 | *infB* | 1.28 | 0.0085 | translation initiation factor IF-2 |
| SAUSA300_1182 |  | 1.57 | 0.0001 | pyruvate ferredoxin oxidoreductase alpha subunit |
| SAUSA300_1183 |  | 1.60 | 0.0001 | pyruvate ferredoxin oxidoreductase beta subunit |
| SAUSA300_1193 | *glpD* | 1.99 | 0.0000 | glycerol-3-phosphate dehydrogenase |
| SAUSA300_1199 |  | 1.44 | 0.0008 | putative aluminium resistance protein |
| SAUSA300_1216 |  | 1.42 | 0.0013 | cardiolipin synthetase |
| SAUSA300_1235 | *guaC* | 1.48 | 0.0101 | guanosine monophosphate reductase |
| SAUSA300_1250 | *parE* | 1.31 | 0.0291 | DNA topoisomerase IV subunit B |
| SAUSA300_1251 | *parC* | 1.42 | 0.0015 | DNA topoisomerase IV subunit A |
| SAUSA300_1254 |  | 1.34 | 0.0060 | putative membrane protein |
| SAUSA300_1283 | *pstS* | 2.05 | 0.0296 | phosphate ABC transporter phosphate-binding protein PstS |
| SAUSA300_1295 |  | 1.61 | 0.0006 | cold shock protein CSD family |
| SAUSA300_1296 |  | 1.57 | 0.0029 | conserved hypothetical protein |
| SAUSA300_1334 |  | 5.26 | 0.0000 | putative membrane protein |
| SAUSA300_1338 |  | 1.31 | 0.0083 | conserved hypothetical protein |
| SAUSA300_1339 |  | 1.40 | 0.0033 | conserved hypothetical protein |
| SAUSA300_1343 | *nth* | 1.38 | 0.0025 | endonuclease III |
| SAUSA300_1344 | *nth* | 1.25 | 0.0499 | putative DNA replication protein DnaD |
| SAUSA300_1346 |  | 1.32 | 0.0111 | putative DnaQ family exonuclease/DinG family helicase |
| SAUSA300_1347 | *birA* | 1.45 | 0.0017 | BirA bifunctional protein |
| SAUSA300_1348 | *birA* | 1.52 | 0.0004 | polyA polymerase |
| SAUSA300_1349 |  | 1.44 | 0.0078 | glycosyl transferase group 1 family protein |
| SAUSA300_1352 |  | 1.27 | 0.0283 | putative membrane protein |
| SAUSA300_1353 |  | 1.39 | 0.0243 | conserved hypothetical protein |
| SAUSA300_1354 |  | 1.44 | 0.0001 | conserved hypothetical protein |
| SAUSA300_1355 | *aroA* | 1.48 | 0.0002 | 3-phosphoshikimate 1-carboxyvinyltransferase |
| SAUSA300_1356 | *aroB* | 1.41 | 0.0050 | 3-dehydroquinate synthase |
| SAUSA300_1357 | *aroC* | 1.48 | 0.0015 | chorismate synthase |
| SAUSA300_1359 |  | 1.40 | 0.0102 | polyprenyl synthetase |
| SAUSA300_1363 | *gpsA* | 1.33 | 0.0161 | glycerol-3-phosphate dehydrogenase NAD-dependent |
| SAUSA300_1364 | *engA* | 1.39 | 0.0429 | GTP-binding protein EngA |
| SAUSA300_1365 | *rpsA* | 1.28 | 0.0170 | 30S ribosomal protein S1 |
| SAUSA300_1369 |  | 1.91 | 0.0000 | pyridine nucleotide-disulfide oxidoreductase |
| SAUSA300_1393 |  | 1.38 | 0.0134 | phiSLT ORF2067-like protein phage tail tape measure protein |
| SAUSA300_1423 | *polA* | 1.91 | 0.0143 | phage related DNA polymerase family A |
| SAUSA300_1440 |  | 2.42 | 0.0000 | conserved hypothetical protein |
| SAUSA300_1449 |  | 1.41 | 0.0110 | MutT/nudix family protein |
| SAUSA300_1456 |  | 1.34 | 0.0323 | alpha glucosidase |
| SAUSA300_1459 | *gnd* | 1.34 | 0.0002 | 6-phosphogluconate dehydrogenase decarboxylating |
| SAUSA300_1460 |  | 1.79 | 0.0000 | peptidase M20/M25/M40 family |
| SAUSA300_1462 |  | 1.71 | 0.0000 | conserved hypothetical protein |
| SAUSA300_1463 |  | 1.70 | 0.0000 | conserved hypothetical protein |
| SAUSA300_1464 |  | 1.39 | 0.0004 | 2-oxoisovalerate dehydrogenase E2 component dihydrolipoamide acetyltransferase |
| SAUSA300_1465 |  | 1.40 | 0.0001 | 2-oxoisovalerate dehydrogenase E1 component beta subunit |
| SAUSA300_1466 |  | 1.43 | 0.0000 | 2-oxoisovalerate dehydrogenase E1 component alpha subunit |
| SAUSA300_1467 | *lpdA* | 1.41 | 0.0000 | 2-oxoisovalerate dehydrogenase E3 component lipoamide dehydrogenase |
| SAUSA300_1468 | *recN* | 1.56 | 0.0000 | DNA repair protein RecN |
| SAUSA300_1469 | *argR* | 1.53 | 0.0001 | arginine repressor |
| SAUSA300_1515 | *fur* | 1.36 | 0.0108 | ABC transporter permease protein |
| SAUSA300_1517 |  | 1.35 | 0.0417 | endonuclease IV |
| SAUSA300_1523 |  | 1.41 | 0.0005 | conserved hypothetical protein |
| SAUSA300_1524 |  | 1.52 | 0.0000 | CBS domain pair protein |
| SAUSA300_1530 |  | 1.28 | 0.0174 | conserved hypothetical protein |
| SAUSA300_1531 | *phoH* | 1.45 | 0.0000 | phosphate starvation-induced protein PhoH family |
| SAUSA300_1538 | *prmA* | 1.69 | 0.0000 | ribosomal protein L11 methyltransferase |
| SAUSA300_1539 | *dnaJ* | 1.61 | 0.0000 | chaperone protein DnaJ |
| SAUSA300_1540 | *dnaK* | 1.30 | 0.0042 | chaperone protein DnaK |
| SAUSA300_1561 |  | 2.36 | 0.0000 | putative membrane protein |
| SAUSA300_1562 |  | 2.39 | 0.0000 | LamB/YcsF family protein |
| SAUSA300_1563 | *accC* | 2.03 | 0.0000 | acetyl-CoA carboxylase biotin carboxylase |
| SAUSA300_1564 | *accB* | 2.23 | 0.0000 | acetyl-CoA carboxylase biotin carboxyl carrier protein |
| SAUSA300_1565 |  | 2.21 | 0.0000 | putative urea amidolyase |
| SAUSA300_1566 |  | 1.73 | 0.0001 | conserved hypothetical protein |
| SAUSA300_1580 |  | 1.60 | 0.0018 | bacterial luciferase family protein |
| SAUSA300_1599 |  | 1.35 | 0.0019 | ACT domain protein PheB |
| SAUSA300_1600 |  | 1.24 | 0.0360 | GTP-binding protein Obg/CgtA |
| SAUSA300_1611 | *valS* | 1.25 | 0.0332 | valyl-tRNA synthetase |
| SAUSA300_1614 | *hemL* | 1.85 | 0.0000 | glutamate-1-semialdehyde-21-aminomutase |
| SAUSA300_1615 | *hemB* | 1.88 | 0.0000 | delta-aminolevulinic acid dehydratase |
| SAUSA300_1616 | *hemD* | 2.23 | 0.0000 | uroporphyrinogen-III synthase |
| SAUSA300_1617 | *hemC* | 2.05 | 0.0000 | porphobilinogen deaminase |
| SAUSA300_1618 | *hemX* | 2.09 | 0.0000 | hemA concentration negative effector hemX |
| SAUSA300_1619 | *hemA* | 2.10 | 0.0000 | glutamyl-tRNA reductase |
| SAUSA300_1620 |  | 1.52 | 0.0029 | probable GTP-binding protein engB |
| SAUSA300_1621 | *clpX* | 1.51 | 0.0005 | ATP-dependent Clp protease ATP-binding subunit ClpX |
| SAUSA300_1622 | *tig* | 1.26 | 0.0472 | trigger factor |
| SAUSA300_1623 |  | 1.28 | 0.0425 | conserved hypothetical protein |
| SAUSA300_1624 |  | 1.41 | 0.0217 | MutT/nudix family protein |
| SAUSA300_1629 | *thrS* | 1.36 | 0.0127 | threonyl-tRNA synthetase |
| SAUSA300_1634 | *coaE* | 1.32 | 0.0065 | dephospho-CoA kinase |
| SAUSA300_1635 | *mutM* | 1.36 | 0.0007 | formamidopyrimidine-DNA glycosylase |
| SAUSA300_1636 | *polA* | 1.48 | 0.0000 | DNA polymerase I superfamily |
| SAUSA300_1639 | *phoP* | 1.33 | 0.0027 | alkaline phosphatase synthesis transcriptional regulatory protein PhoP |
| SAUSA300_1644 | *pyk* | 1.42 | 0.0003 | pyruvate kinase |
| SAUSA300_1645 | *pfkA* | 1.58 | 0.0000 | 6-phosphofructokinase |
| SAUSA300_1646 | *accA* | 1.46 | 0.0009 | acetyl-CoA carboxylase carboxyl transferase alpha subunit |
| SAUSA300_1647 | *accD* | 1.40 | 0.0113 | acetyl-CoA carboxylase carboxyl transferase beta subunit |
| SAUSA300_1648 |  | 1.39 | 0.0112 | putative NADP-dependent malic enzyme |
| SAUSA300_1653 |  | 1.42 | 0.0069 | conserved hypothetical protein |
| SAUSA300_1656 |  | 1.45 | 0.0093 | universal stress protein family |
| SAUSA300_1658 |  | 1.47 | 0.0011 | conserved hypothetical protein |
| SAUSA300_1664 | *ezrA* | 1.20 | 0.0425 | septation ring formation regulator EzrA |
| SAUSA300_1676 | *sgtA* | 1.32 | 0.0496 | probable transglycosylase |
| SAUSA300_1679 | *acsA* | 2.34 | 0.0023 | acetyl-coenzyme A synthetase |
| SAUSA300_1682 | *ccpA* | 1.24 | 0.0236 | catabolite control protein A |
| SAUSA300_1686 | *murC* | 1.49 | 0.0000 | UDP-N-acetylmuramate--alanine ligase |
| SAUSA300_1687 |  | 1.43 | 0.0001 | FtsK/SpoIIIE family protein |
| SAUSA300_1688 |  | 1.30 | 0.0177 | phenylalanyl-tRNA synthetase (beta subunit) |
| SAUSA300_1691 |  | 1.37 | 0.0005 | glutamyl-aminopeptidase |
| SAUSA300_1700 |  | 1.27 | 0.0135 | polysaccharide biosynthesis protein |
| SAUSA300_1704 | *leuS* | 1.27 | 0.0155 | leucyl-tRNA synthetase |
| SAUSA300_1728 |  | 1.47 | 0.0028 | oxidoreductase aldo/keto reductase family |
| SAUSA300_1760 | *epiG* | 1.79 | 0.0001 | lantibiotic epidermin immunity protein F |
| SAUSA300_1761 | *epiE* | 2.08 | 0.0000 | lantibiotic epidermin immunity protein F |
| SAUSA300_1762 | *epiF* | 1.90 | 0.0000 | lantibiotic epidermin immunity protein F |
| SAUSA300_1763 | *epiP* | 1.83 | 0.0000 | lantibiotic epidermin leader peptide processing serine protease EpiP |
| SAUSA300_1781 | *hemG* | 1.38 | 0.0002 | protoporphyrinogen oxidase |
| SAUSA300_1782 | *hemH* | 1.32 | 0.0057 | ferrochelatase |
| SAUSA300_1783 | *hemE* | 1.29 | 0.0177 | uroporphyrinogen decarboxylase |
| SAUSA300_1791 | *cbf1* | 1.40 | 0.0004 | cmp-binding-factor 1 |
| SAUSA300_1792 |  | 1.51 | 0.0000 | conserved hypothetical protein |
| SAUSA300_1793 |  | 1.35 | 0.0080 | conserved hypothetical protein |
| SAUSA300_1801 | *fumC* | 1.43 | 0.0003 | fumarate hydratase class II |
| SAUSA300_1847 |  | 1.57 | 0.0000 | conserved hypothetical protein |
| SAUSA300_1862 |  | 1.28 | 0.0360 | conserved hypothetical protein |
| SAUSA300_1864 |  | 1.45 | 0.0029 | putative membrane protein |
| SAUSA300_1874 |  | 1.61 | 0.0006 | ferritins family protein |
| SAUSA300_1876 |  | 1.33 | 0.0323 | DNA polymerase IV |
| SAUSA300_1884 |  | 1.33 | 0.0069 | CamS sex pheromone cAM373 precursor |
| SAUSA300_1885 | *ligA* | 1.45 | 0.0003 | DNA ligase |
| SAUSA300_1886 | *pcrA* | 1.48 | 0.0001 | ATP-dependent DNA helicase PcrA |
| SAUSA300_1895 |  | 1.75 | 0.0000 | nitric oxide synthase oxygenase |
| SAUSA300_1896 | *pheA* | 1.57 | 0.0123 | prephenate dehydratase |
| SAUSA300_1909 |  | 1.40 | 0.0009 | conserved hypothetical protein |
| SAUSA300_1922 | *sak* | 1.79 | 0.0000 | staphylokinase precursor |
| SAUSA300_1976 |  | 1.39 | 0.0133 | probable succinyl-diaminopimelate desuccinylase |
| SAUSA300_1978 |  | 1.61 | 0.0138 | ferric hydroxamate receptor |
| SAUSA300_1981 |  | 1.56 | 0.0387 | phage terminase family protein |
| SAUSA300_1983 | *groES* | 1.27 | 0.0386 | 10 kDa chaperonin |
| SAUSA300_1996 | *amt* | 2.47 | 0.0000 | ammonium transporter |
| SAUSA300_2008 | *ilvN* | 1.46 | 0.0423 | acetolactate synthase small subunit |
| SAUSA300_2024 | *rsbV* | 1.31 | 0.0054 | anti-sigma-B factor antagonist |
| SAUSA300_2027 | *alr* | 1.29 | 0.0092 | alanine racemase |
| SAUSA300_2028 | *acpS* | 1.32 | 0.0175 | holo-(acyl-carrier-protein) synthase |
| SAUSA300_2030 |  | 1.32 | 0.0040 | putative membrane protein |
| SAUSA300_2042 |  | 1.45 | 0.0091 | conserved hypothetical protein |
| SAUSA300_2058 | *atpD* | 1.38 | 0.0008 | ATP synthase F1 beta subunit |
| SAUSA300_2059 | *atpG* | 1.34 | 0.0024 | ATP synthase F1 gamma subunit |
| SAUSA300_2060 | *atpA* | 1.36 | 0.0030 | ATP synthase F1 alpha subunit |
| SAUSA300_2061 | *atpH* | 1.30 | 0.0110 | ATP synthase F1 delta subunit |
| SAUSA300_2062 | *atpF* | 1.31 | 0.0157 | ATP synthase F0 B subunit |
| SAUSA300_2063 | *atpE* | 1.33 | 0.0155 | ATP synthase F0 C subunit |
| SAUSA300_2064 | *atpB* | 1.52 | 0.0001 | ATP synthase F0 A subunit |
| SAUSA300_2079 | *fba* | 1.27 | 0.0322 | fructose bisphosphate aldolase |
| SAUSA300_2081 | *pyrG* | 1.81 | 0.0000 | CTP synthase |
| SAUSA300_2086 |  | 1.49 | 0.0019 | conserved hypothetical protein |
| SAUSA300_2093 |  | 1.67 | 0.0004 | conserved hypothetical protein |
| SAUSA300_2097 |  | 2.06 | 0.0000 | conserved hypothetical protein |
| SAUSA300_2098 | *arsR* | 7.52 | 0.0000 | transcriptional repressor ArsR family |
| SAUSA300_2099 |  | 5.78 | 0.0000 | cation efflux family protein |
| SAUSA300_2103 |  | 1.39 | 0.0138 | ABC transporter ATP-binding protein |
| SAUSA300_2104 | *glmS* | 1.87 | 0.0000 | glucosamine--fructose-6-phosphate aminotransferase (isomerizing) |
| SAUSA300_2125 |  | 1.31 | 0.0065 | ATP-binding protein Mrp/Nbp35 family |
| SAUSA300_2142 | *asp23* | 1.39 | 0.0124 | alkaline shock protein 23 |
| SAUSA300_2143 |  | 1.60 | 0.0001 | conserved hypothetical protein |
| SAUSA300_2144 |  | 1.64 | 0.0000 | conserved hypothetical protein |
| SAUSA300_2145 |  | 1.90 | 0.0000 | glycine betaine transporter |
| SAUSA300_2150 | *lacE* | 1.95 | 0.0105 | PTS system lactose-specific IIBC component |
| SAUSA300_2152 | *lacD* | 2.82 | 0.0002 | tagatose 16-diphosphate aldolase |
| SAUSA300_2153 | *lacC* | 3.15 | 0.0002 | tagatose-6-phosphate kinase |
| SAUSA300_2154 | *lacB* | 2.84 | 0.0210 | galactose-6-phosphate isomerase |
| SAUSA300_2155 | *lacA* | 4.77 | 0.0000 | galactose-6-phosphate isomerase |
| SAUSA300_2161 | *hysA* | 1.43 | 0.0363 | Hyaluronate lyase precursor |
| SAUSA300_2163 |  | 1.41 | 0.0134 | conserved hypothetical protein |
| SAUSA300_2164 |  | 1.63 | 0.0022 | conserved hypothetical protein |
| SAUSA300_2182 | *infA* | 1.26 | 0.0458 | translation initiation factor IF-1 |
| SAUSA300_2197 | *rplP* | 1.27 | 0.0341 | 50S ribosomal protein L16 |
| SAUSA300_2199 | *rplV* | 1.24 | 0.0370 | 50S ribosomal protein L22 |
| SAUSA300_2203 | *rplD* | 1.24 | 0.0274 | 50S ribosomal protein L4 |
| SAUSA300_2227 | *moeB* | 1.41 | 0.0028 | molybdopterin biosynthesis protein B |
| SAUSA300_2230 | *modA* | 1.34 | 0.0039 | molybdenum ABC transporter molybdenum-binding protein ModA |
| SAUSA300_2257 |  | 1.43 | 0.0080 | conserved hypothetical protein |
| SAUSA300_2258 |  | 1.35 | 0.0408 | formate dehydrogenase alpha subunit |
| SAUSA300_2268 |  | 1.44 | 0.0098 | sodium/bile acid symporter family protein |
| SAUSA300_2278 | *hutU* | 1.54 | 0.0060 | urocanate hydratase |
| SAUSA300_2288 |  | 1.94 | 0.0030 | ABC transporter ATP-binding protein |
| SAUSA300_2309 |  | 1.34 | 0.0239 | sensor histidine kinase |
| SAUSA300_2315 |  | 1.39 | 0.0017 | putative lipoprotein |
| SAUSA300_2320 |  | 1.33 | 0.0207 | conserved hypothetical protein |
| SAUSA300_2328 |  | 1.43 | 0.0298 | conserved hypothetical protein |
| SAUSA300_2343 |  | 3.25 | 0.0354 | respiratory nitrate reductase alpha subunit |
| SAUSA300_2347 | *nirR* | 1.78 | 0.0132 | nitrite reductase transcriptional regulator NirR |
| SAUSA300_2349 |  | 1.28 | 0.0439 | formate/nitrite transporter family protein |
| SAUSA300_2350 |  | 1.44 | 0.0449 | conserved hypothetical protein |
| SAUSA300_2390 | *opuCd* | 2.06 | 0.0000 | glycine betaine/carnitine/choline transport system permease |
| SAUSA300_2391 | *opuCc* | 2.20 | 0.0000 | glycine betaine/carnitine/choline ABC transporter |
| SAUSA300_2392 | *opuCb* | 2.52 | 0.0000 | glycine betaine/carnitine/choline ABC transporter |
| SAUSA300_2393 | *opuCa* | 2.68 | 0.0000 | glycine betaine/carnitine/choline ABC transporter ATP-binding protein |
| SAUSA300_2395 |  | 1.90 | 0.0001 | amino acid permease |
| SAUSA300_2399 |  | 1.47 | 0.0077 | ABC transporter ATP-binding protein |
| SAUSA300_2416 |  | 1.52 | 0.0008 | glucose 1-dehydrogenase-like protein |
| SAUSA300_2422 |  | 1.33 | 0.0492 | oxidoreductase short-chain dehydrogenase/reductase family |
| SAUSA300_2462 | *frp* | 1.47 | 0.0038 | NAD(P)H-flavin oxidoreductase |
| SAUSA300_2463 | *ddh* | 1.51 | 0.0212 | D-lactate dehydrogenase |
| SAUSA300_2468 |  | 1.49 | 0.0405 | acetyltransferase GNAT family |
| SAUSA300_2476 | *ptsG* | 1.65 | 0.0033 | phosphotransferase system glucose-specific IIABC component |
| SAUSA300_2485 |  | 1.50 | 0.0003 | methylated DNA-protein cysteine methyltransferase |
| SAUSA300_2486 |  | 2.72 | 0.0000 | putative ATP-dependent Clp proteinase |
| SAUSA300_2487 | *feoB* | 1.77 | 0.0017 | ferrous iron transport protein B |
| SAUSA300_2494 |  | 1.54 | 0.0001 | copper-translocating P-type ATPase |
| SAUSA300_2495 |  | 1.85 | 0.0000 | copper chaperone copZ |
| SAUSA300_2498 | *crtN* | 1.89 | 0.0000 | squalene synthase |
| SAUSA300_2499 | *crtM* | 2.02 | 0.0000 | squalene desaturase |
| SAUSA300_2500 |  | 2.10 | 0.0000 | glycosyl transferase |
| SAUSA300_2501 |  | 1.86 | 0.0000 | phytoene dehydrogenase |
| SAUSA300_2502 |  | 1.83 | 0.0000 | conserved hypothetical protein |
| SAUSA300_2522 |  | 1.83 | 0.0229 | conserved hypothetical protein |
| SAUSA300_2525 |  | 1.68 | 0.0002 | conserved hypothetical protein |
| SAUSA300_2526 | *pyrD* | 1.49 | 0.0002 | dihydroorotate dehydrogenase |
| SAUSA300_2529 |  | 1.69 | 0.0000 | conserved hypothetical protein |
| SAUSA300_2537 |  | 1.26 | 0.0211 | L-lactate dehydrogenase |
| SAUSA300_2539 |  | 1.42 | 0.0109 | aminotransferase |
| SAUSA300_2545 | *betA* | 2.42 | 0.0000 | choline dehydrogenase |
| SAUSA300_2546 | *betB* | 2.06 | 0.0000 | glycine betaine aldehyde dehydrogenase |
| SAUSA300_2547 |  | 1.70 | 0.0000 | conserved hypothetical protein |
| SAUSA300_2549 | *bccT* | 1.43 | 0.0173 | choline/carnitine/betaine transporter BCCT family |
| SAUSA300_2554 |  | 1.35 | 0.0222 | sulfite reductase flavoprotein |
| SAUSA300_2558 |  | 1.39 | 0.0082 | sensor histidine kinase |
| SAUSA300_2573 | *isaB* | 2.32 | 0.0000 | immunodominant antigen B |
| SAUSA300_2581 |  | 2.31 | 0.0000 | putative surface anchored protein |
| SAUSA300_2592 |  | 1.51 | 0.0234 | conserved hypothetical protein |
| SAUSA300_2626 |  | 2.30 | 0.0000 | conserved hypothetical protein |
| SAUSA300_2632 |  | 1.55 | 0.0033 | putative membrane protein |
| SAUSA300_2641 |  | 1.47 | 0.0165 | conserved hypothetical protein |
| SAUSA300_2642 |  | 1.88 | 0.0008 | conserved hypothetical protein |
| SAUSA300_2644 | *gidB* | 1.26 | 0.0490 | glucose-inhibited division protein B |
