# Supplementary material for: Calprotectin Increases the Activity of the SaeRS Two Component System and Murine Mortality during Staphylococcus aureus Infections
Source: PLoS Pathog. 2015 Jul 6;11(7):e1005026. doi: 10.1371/journal.ppat.1005026 (PMC4492782; doi:10.1371/journal.ppat.1005026)
Supplement: S6 Table — (DOCX) [file ppat.1005026.s013.docx]

**S6 Table. Genes down-regulated by 20 µM Zn/1.1 µM CP treatment**

| **ID** | **Name** | **Fold Change** | **p value** | **Gene product** |
| --- | --- | --- | --- | --- |
| SAUSA300_0037 | *ccrB* | 0.57 | 0.0001 | cassette chromosome recombinase B |
| SAUSA300_0038 | *ccrA* | 0.64 | 0.0174 | cassette chromosome recombinase A |
| SAUSA300_0042 |  | 0.81 | 0.0132 | conserved hypothetical protein |
| SAUSA300_0053 | *speG* | 0.75 | 0.0088 | Spermidine N(1)-acetyltransferase |
| SAUSA300_0062 | *arcB* | 0.68 | 0.0043 | ornithine carbamoyltransferase |
| SAUSA300_0073 |  | 0.64 | 0.0002 | peptide ABC transporter peptide-binding protein |
| SAUSA300_0074 | *opp* | 0.58 | 0.0014 | oligopeptide permease channel-forming protein |
| SAUSA300_0075 | *opp* | 0.63 | 0.0136 | oligopeptide permease channel-forming protein |
| SAUSA300_0076 |  | 0.61 | 0.0047 | ABC transporter ATP-binding protein |
| SAUSA300_0077 |  | 0.57 | 0.0337 | ABC transporter ATP-binding protein |
| SAUSA300_0082 |  | 0.73 | 0.0170 | conserved hypothetical protein |
| SAUSA300_0088 |  | 0.55 | 0.0152 | hypothetical protein |
| SAUSA300_0089 |  | 0.70 | 0.0016 | Probable tRNA-dihydrouridine synthase |
| SAUSA300_0091 |  | 0.63 | 0.0294 | putative permease |
| SAUSA300_0093 |  | 0.73 | 0.0253 | transcriptional regulator LysR family domain protein |
| SAUSA300_0112 | *lctP* | 0.72 | 0.0032 | L-lactate permease |
| SAUSA300_0113 |  | 0.64 | 0.0063 | immunoglobulin G binding protein A precursor |
| SAUSA300_0114 |  | 0.72 | 0.0000 | staphylococcal accessory regulator |
| SAUSA300_0115 | *sirC* | 0.62 | 0.0019 | iron compound ABC transporter permease protein SirC |
| SAUSA300_0116 | *sirB* | 0.42 | 0.0000 | iron compound ABC transporter permease protein SirB |
| SAUSA300_0120 | *sbnC* | 0.59 | 0.0146 | siderophore biosynthesis protein IucC family |
| SAUSA300_0123 |  | 0.62 | 0.0368 | siderophore biosynthesis protein IucC family |
| SAUSA300_0124 |  | 0.58 | 0.0064 | HPCH/HPAI aldolase family protein |
| SAUSA300_0125 |  | 0.50 | 0.0000 | pyridoxal-dependent decarboxylase |
| SAUSA300_0126 |  | 0.47 | 0.0001 | conserved hypothetical protein |
| SAUSA300_0135 |  | 0.63 | 0.0001 | Superoxide dismutase (Mn/Fe family) |
| SAUSA300_0136 |  | 0.71 | 0.0022 | cell wall surface anchor family protein |
| SAUSA300_0168 |  | 0.48 | 0.0000 | conserved hypothetical protein |
| SAUSA300_0169 |  | 0.48 | 0.0000 | conserved hypothetical protein |
| SAUSA300_0178 |  | 0.39 | 0.0000 | conserved hypothetical protein |
| SAUSA300_0181 |  | 0.44 | 0.0039 | non-ribosomal peptide synthetase |
| SAUSA300_0182 |  | 0.40 | 0.0000 | 4'-phosphopantetheinyl transferase superfamily protein |
| SAUSA300_0189 | *entB* | 0.76 | 0.0370 | isochorismatase |
| SAUSA300_0190 | *ipdC* | 0.79 | 0.0360 | indole-3-pyruvate decarboxylase |
| SAUSA300_0192 |  | 0.56 | 0.0004 | conserved hypothetical protein |
| SAUSA300_0193 |  | 0.56 | 0.0004 | conserved hypothetical protein |
| SAUSA300_0194 |  | 0.54 | 0.0001 | sucrose-specific PTS tranporter protein |
| SAUSA300_0195 |  | 0.47 | 0.0000 | RpiR family transcriptional regulator |
| SAUSA300_0200 |  | 0.50 | 0.0000 | peptide ABC transporter ATP-binding protein |
| SAUSA300_0204 | *ggt* | 0.68 | 0.0059 | gamma-glutamyltranspeptidase |
| SAUSA300_0206 |  | 0.61 | 0.0000 | flavodoxin family protein |
| SAUSA300_0207 |  | 0.42 | 0.0000 | conserved hypothetical protein |
| SAUSA300_0215 |  | 0.35 | 0.0000 | conserved hypothetical protein |
| SAUSA300_0216 | *uhpT* | 0.63 | 0.0069 | hexose phosphate transport protein |
| SAUSA300_0223 |  | 0.29 | 0.0000 | conserved hypothetical protein |
| SAUSA300_0224 | *coa* | 0.69 | 0.0108 | staphylocoagulase precursor |
| SAUSA300_0230 |  | 0.73 | 0.0124 | putative membrane protein |
| SAUSA300_0237 |  | 0.71 | 0.0249 | inosine-uridine preferring nucleoside hydrolase |
| SAUSA300_0268 |  | 0.45 | 0.0000 | putative drug transporter |
| SAUSA300_0270 | *lytM* | 0.65 | 0.0002 | peptidoglycan hydrolase |
| SAUSA300_0306 | *brnQ* | 0.76 | 0.0092 | branched-chain amino acid transport system II carrier protein |
| SAUSA300_0314 |  | 0.63 | 0.0072 | sodium:solute symporter family protein |
| SAUSA300_0320 |  | 0.50 | 0.0000 | triacylglycerol lipase precursor |
| SAUSA300_0330 |  | 0.40 | 0.0027 | putative transport protein SgaT |
| SAUSA300_0331 |  | 0.41 | 0.0000 | conserved hypothetical protein |
| SAUSA300_0332 |  | 0.40 | 0.0000 | PTS system IIA component |
| SAUSA300_0333 |  | 0.48 | 0.0000 | transcriptional antiterminator BglG family |
| SAUSA300_0334 |  | 0.63 | 0.0028 | transcriptional regulator MarR family |
| SAUSA300_0337 | *glpT* | 0.47 | 0.0000 | glycerol-3-phosphate transporter |
| SAUSA300_0347 | *tatC* | 0.61 | 0.0222 | Sec-independent protein translocase TatC |
| SAUSA300_0348 |  | 0.63 | 0.0362 | twin-arginine translocation protein TatA/E family |
| SAUSA300_0353 |  | 0.65 | 0.0002 | conserved hypothetical protein |
| SAUSA300_0356 |  | 0.67 | 0.0069 | conserved hypothetical protein |
| SAUSA300_0357 | *metE* | 0.57 | 0.0003 | 5-methyltetrahydropteroyltriglutamate--homocysteine S-methyltransferase |
| SAUSA300_0358 |  | 0.65 | 0.0073 | putative 5-methyltetrahydrofolate--homocysteine methyltransferase |
| SAUSA300_0359 |  | 0.66 | 0.0310 | trans-sulfuration enzyme family protein |
| SAUSA300_0361 |  | 0.70 | 0.0048 | ParB-like partition protein |
| SAUSA300_0365 |  | 0.53 | 0.0002 | conserved hypothetical protein |
| SAUSA300_0366 | *rpsF* | 0.77 | 0.0051 | ribosomal protein S6 |
| SAUSA300_0367 | *ssb* | 0.75 | 0.0024 | single-strand binding protein |
| SAUSA300_0370 |  | 0.53 | 0.0000 | putative staphylococcal enterotoxin |
| SAUSA300_0373 |  | 0.66 | 0.0021 | conserved hypothetical protein |
| SAUSA300_0377 |  | 0.76 | 0.0275 | putative lipoprotein |
| SAUSA300_0378 |  | 0.58 | 0.0124 | conserved hypothetical protein |
| SAUSA300_0379 | *ahpF* | 0.58 | 0.0000 | alkyl hydroperoxide reductase subunit F |
| SAUSA300_0380 | *ahpC* | 0.57 | 0.0000 | Alkyl hydroperoxide reductase subunit C |
| SAUSA300_0382 |  | 0.52 | 0.0000 | sodium:dicarboxylate symporter family protein |
| SAUSA300_0390 |  | 0.56 | 0.0000 | conserved hypothetical protein |
| SAUSA300_0396 | *set7* | 0.33 | 0.0000 | exotoxin 7 |
| SAUSA300_0397 |  | 0.64 | 0.0032 | exotoxin |
| SAUSA300_0398 |  | 0.43 | 0.0000 | exotoxin |
| SAUSA300_0399 |  | 0.64 | 0.0451 | exotoxin |
| SAUSA300_0400 |  | 0.62 | 0.0041 | exotoxin |
| SAUSA300_0401 |  | 0.70 | 0.0423 | exotoxin |
| SAUSA300_0402 |  | 0.57 | 0.0004 | exotoxin |
| SAUSA300_0403 |  | 0.50 | 0.0007 | exotoxin |
| SAUSA300_0404 |  | 0.64 | 0.0017 | exotoxin |
| SAUSA300_0407 |  | 0.39 | 0.0000 | exotoxin |
| SAUSA300_0408 |  | 0.54 | 0.0000 | putative surface protein |
| SAUSA300_0424 |  | 0.57 | 0.0008 | putative cobalamin synthesis protein |
| SAUSA300_0429 |  | 0.76 | 0.0084 | PAP2 family protein |
| SAUSA300_0430 |  | 0.76 | 0.0061 | conserved hypothetical protein |
| SAUSA300_0432 |  | 0.17 | 0.0000 | sodium dependent transporter |
| SAUSA300_0435 |  | 0.43 | 0.0005 | ABC transporter ATP-binding protein |
| SAUSA300_0436 |  | 0.52 | 0.0229 | ABC transporter permease protein |
| SAUSA300_0437 |  | 0.50 | 0.0000 | NLPA lipoprotein |
| SAUSA300_0440 |  | 0.69 | 0.0093 | MutT/nudix family protein |
| SAUSA300_0442 |  | 0.70 | 0.0118 | YibE/F-like protein |
| SAUSA300_0447 |  | 0.65 | 0.0134 | tRNA-Ser |
| SAUSA300_0453 |  | 0.65 | 0.0000 | conserved hypothetical protein |
| SAUSA300_0454 | *recR* | 0.54 | 0.0000 | recombination protein RecR |
| SAUSA300_0471 |  | 0.76 | 0.0014 | veg protein |
| SAUSA300_0472 | *ispE* | 0.62 | 0.0000 | 4-diphosphocytidyl-2C-methyl-D-erythritol kinase |
| SAUSA300_0481 | *mfd* | 0.73 | 0.0053 | transcription-repair coupling factor |
| SAUSA300_0482 | *mfd* | 0.75 | 0.0109 | polysaccharide biosynthesis protein |
| SAUSA300_0483 |  | 0.76 | 0.0059 | tetrapyrrole methylase family protein |
| SAUSA300_0484 |  | 0.75 | 0.0054 | conserved hypothetical protein |
| SAUSA300_0485 |  | 0.76 | 0.0166 | cell-division initiation protein |
| SAUSA300_0486 |  | 0.61 | 0.0000 | polyribonucleotide nucleotidyltransferase |
| SAUSA300_0495 |  | 0.68 | 0.0059 | hypothetical protein |
| SAUSA300_0500 |  | 0.66 | 0.0036 | tRNA-Ile |
| SAUSA300_0504 |  | 0.44 | 0.0000 | pyridoxine biosynthesis protein |
| SAUSA300_0505 |  | 0.42 | 0.0000 | conserved hypothetical protein |
| SAUSA300_0511 | *radA* | 0.72 | 0.0002 | DNA repair protein RadA |
| SAUSA300_0512 |  | 0.75 | 0.0017 | PIN domain protein |
| SAUSA300_0522 | *rplK* | 0.81 | 0.0217 | ribosomal protein L11 |
| SAUSA300_0523 | *rplA* | 0.74 | 0.0010 | ribosomal protein L1 |
| SAUSA300_0524 | *rplJ* | 0.76 | 0.0089 | ribosomal protein L10 |
| SAUSA300_0525 | *rplL* | 0.74 | 0.0073 | ribosomal protein L7/L12 |
| SAUSA300_0527 | *rpoB* | 0.77 | 0.0017 | DNA-directed RNA polymerase beta subunit |
| SAUSA300_0528 | *rpoC* | 0.75 | 0.0018 | DNA-directed RNA polymerase beta' subunit |
| SAUSA300_0536 |  | 0.66 | 0.0108 | DJ-1/PfpI family protein |
| SAUSA300_0538 |  | 0.51 | 0.0000 | NAD dependent epimerase/dehydratase family |
| SAUSA300_0541 |  | 0.65 | 0.0040 | deoxynucleoside kinase family protein |
| SAUSA300_0551 |  | 0.58 | 0.0000 | conserved hypothetical protein |
| SAUSA300_0552 |  | 0.57 | 0.0000 | conserved hypothetical protein |
| SAUSA300_0553 |  | 0.64 | 0.0000 | conserved hypothetical protein |
| SAUSA300_0575 |  | 0.54 | 0.0000 | conserved hypothetical protein |
| SAUSA300_0602 |  | 0.63 | 0.0000 | conserved hypothetical protein |
| SAUSA300_0621 |  | 0.73 | 0.0002 | iron-dependent repressor |
| SAUSA300_0622 |  | 0.44 | 0.0000 | putative membrane protein |
| SAUSA300_0629 | *pbp4* | 0.69 | 0.0043 | penicillin-binding protein 4 |
| SAUSA300_0630 |  | 0.55 | 0.0000 | ABC transporter ATP-binding protein |
| SAUSA300_0633 | *fhuA* | 0.64 | 0.0000 | ferrichrome transport ATP-binding protein fhuA |
| SAUSA300_0634 | *fhuB* | 0.68 | 0.0000 | ferrichrome transport permease protein fhuB |
| SAUSA300_0635 | *fhuG* | 0.65 | 0.0000 | ferrichrome transport permease protein fhuG |
| SAUSA300_0640 |  | 0.78 | 0.0142 | putative membrane protein |
| SAUSA300_0641 |  | 0.52 | 0.0022 | putative lipase/esterase |
| SAUSA300_0642 |  | 0.65 | 0.0000 | conserved hypothetical protein |
| SAUSA300_0672 |  | 0.69 | 0.0022 | transcriptional regulator MarR family |
| SAUSA300_0678 |  | 0.71 | 0.0289 | putative membrane protein |
| SAUSA300_0690 | *saeS* | 0.65 | 0.0000 | sensor histidine kinase SaeS |
| SAUSA300_0691 | *saeR* | 0.64 | 0.0000 | DNA-binding response regulator SaeR |
| SAUSA300_0692 | *saeQ* | 0.65 | 0.0000 | SaeS regulatory protein SaeQ |
| SAUSA300_0693 | *saeP* | 0.58 | 0.0000 | SaeS regulatory protein SaeP |
| SAUSA300_0703 |  | 0.66 | 0.0000 | sulfatase family protein |
| SAUSA300_0712 |  | 0.66 | 0.0000 | amino acid/peptide transporter (Peptide:H+ symporter) |
| SAUSA300_0713 | *folE* | 0.71 | 0.0151 | GTP cyclohydrolase I |
| SAUSA300_0714 |  | 0.79 | 0.0267 | Integral membrane protein |
| SAUSA300_0715 | *nrdI* | 0.33 | 0.0000 | nrdI protein |
| SAUSA300_0716 | *nrdI* | 0.35 | 0.0000 | ribonucleoside-diphosphate reductase alpha subunit |
| SAUSA300_0717 |  | 0.39 | 0.0000 | ribonucleoside-diphosphate reductase beta subunit |
| SAUSA300_0724 |  | 0.81 | 0.0266 | putative lipoprotein |
| SAUSA300_0725 |  | 0.77 | 0.0304 | conserved hypothetical protein |
| SAUSA300_0732 |  | 0.72 | 0.0121 | conserved hypothetical protein |
| SAUSA300_0761 |  | 0.75 | 0.0141 | conserved hypothetical protein |
| SAUSA300_0773 |  | 0.58 | 0.0004 | putative staphylocoagulase |
| SAUSA300_0774 | *empbp* | 0.48 | 0.0000 | secretory extracellular matrix and plasma binding protein |
| SAUSA300_0780 |  | 0.53 | 0.0004 | conserved hypothetical protein |
| SAUSA300_0783 |  | 0.54 | 0.0003 | phosphoglycerate mutase family protein |
| SAUSA300_0784 |  | 0.70 | 0.0003 | LysE/YggA family protein |
| SAUSA300_0788 |  | 0.50 | 0.0000 | nitroreductase family protein |
| SAUSA300_0789 |  | 0.63 | 0.0003 | putative thioredoxin |
| SAUSA300_0796 |  | 0.36 | 0.0000 | ABC transporter ATP-binding protein |
| SAUSA300_0797 |  | 0.31 | 0.0000 | ABC transporter permease protein |
| SAUSA300_0798 |  | 0.29 | 0.0000 | ABC transporter substrate-binding protein |
| SAUSA300_0815 | *ear* | 0.54 | 0.0000 | Ear protein |
| SAUSA300_0829 | *lipA* | 0.74 | 0.0003 | lipoic acid synthetase |
| SAUSA300_0830 |  | 0.60 | 0.0001 | conserved hypothetical protein |
| SAUSA300_0831 |  | 0.54 | 0.0033 | conserved hypothetical protein |
| SAUSA300_0846 |  | 0.18 | 0.0000 | Na+/H+ antiporter family protein |
| SAUSA300_0847 |  | 0.43 | 0.0000 | conserved hypothetical protein |
| SAUSA300_0858 |  | 0.80 | 0.0178 | conserved hypothetical protein |
| SAUSA300_0866 |  | 0.71 | 0.0002 | conserved hypothetical protein |
| SAUSA300_0867 | *spsA* | 0.71 | 0.0013 | signal peptidase IA |
| SAUSA300_0884 |  | 0.75 | 0.0108 | conserved hypothetical protein |
| SAUSA300_0899 |  | 0.79 | 0.0338 | putative negative regulator of genetic competence |
| SAUSA300_0950 | *sspB* | 0.67 | 0.0229 | cysteine protease precursor |
| SAUSA300_0952 |  | 0.64 | 0.0028 | aminotransferase class I |
| SAUSA300_0953 |  | 0.64 | 0.0120 | putative membrane protein |
| SAUSA300_0964 |  | 0.38 | 0.0000 | chitinase-related protein |
| SAUSA300_0977 |  | 0.58 | 0.0014 | cobalt transport family protein |
| SAUSA300_0978 |  | 0.61 | 0.0002 | ABC transporter ATP-binding protein |
| SAUSA300_0989 |  | 0.71 | 0.0339 | conserved hypothetical protein |
| SAUSA300_0990 |  | 0.66 | 0.0149 | conserved hypothetical protein |
| SAUSA300_0991 | *def* | 0.53 | 0.0000 | peptide deformylase |
| SAUSA300_0992 |  | 0.72 | 0.0022 | putative lipoprotein |
| SAUSA300_1004 |  | 0.71 | 0.0020 | conserved hypothetical protein |
| SAUSA300_1006 |  | 0.54 | 0.0000 | conserved hypothetical protein |
| SAUSA300_1008 |  | 0.74 | 0.0170 | conserved hypothetical protein |
| SAUSA300_1012 |  | 0.73 | 0.0004 | conserved hypothetical protein |
| SAUSA300_1014 | *pyc* | 0.74 | 0.0031 | pyruvate carboxylase |
| SAUSA300_1028 |  | 0.66 | 0.0114 | iron transport associated domain protein |
| SAUSA300_1029 |  | 0.54 | 0.0000 | iron transport associated domain protein |
| SAUSA300_1030 |  | 0.47 | 0.0000 | iron transport associated domain protein |
| SAUSA300_1031 |  | 0.44 | 0.0000 | conserved hypothetical protein |
| SAUSA300_1032 |  | 0.61 | 0.0001 | putative iron compound ABC transporter iron compound-binding protein |
| SAUSA300_1033 |  | 0.53 | 0.0000 | iron/heme permease |
| SAUSA300_1034 | *srtB* | 0.60 | 0.0006 | sortase B |
| SAUSA300_1035 |  | 0.59 | 0.0007 | conserved hypothetical protein |
| SAUSA300_1039 | *rnhC* | 0.65 | 0.0317 | ribonuclease HIII |
| SAUSA300_1053 |  | 0.35 | 0.0000 | conserved hypothetical protein |
| SAUSA300_1058 |  | 0.48 | 0.0000 | alpha-hemolysin precursor |
| SAUSA300_1059 |  | 0.48 | 0.0010 | putative exotoxin 1 |
| SAUSA300_1082 |  | 0.69 | 0.0009 | conserved hypothetical protein |
| SAUSA300_1083 |  | 0.69 | 0.0021 | conserved hypothetical protein |
| SAUSA300_1084 |  | 0.73 | 0.0289 | conserved hypothetical protein |
| SAUSA300_1099 |  | 0.56 | 0.0000 | conserved hypothetical protein |
| SAUSA300_1117 | *rpmB* | 0.70 | 0.0009 | 50S ribosomal protein L28 |
| SAUSA300_1131 | *rpsP* | 0.72 | 0.0035 | 30S ribosomal protein S16 |
| SAUSA300_1141 |  | 0.69 | 0.0355 | endopeptidase resistance gene |
| SAUSA300_1149 | *rpsB* | 0.43 | 0.0000 | 30S ribosomal protein S2 |
| SAUSA300_1150 | *tsf* | 0.78 | 0.0048 | translation elongation factor Ts |
| SAUSA300_1166 | *rpsO* | 0.69 | 0.0002 | 30S ribosomal protein S15 |
| SAUSA300_1179 |  | 0.81 | 0.0218 | conserved hypothetical protein |
| SAUSA300_1221 |  | 0.72 | 0.0300 | conserved hypothetical protein |
| SAUSA300_1225 |  | 0.49 | 0.0000 | aspartate kinase |
| SAUSA300_1226 |  | 0.80 | 0.0208 | homoserine dehydrogenase |
| SAUSA300_1229 |  | 0.77 | 0.0015 | hydrolase haloacid dehalogenase-like family |
| SAUSA300_1234 | *rpmN* | 0.52 | 0.0000 | 30S ribosomal protein S14-2 |
| SAUSA300_1238 |  | 0.56 | 0.0017 | conserved hypothetical protein |
| SAUSA300_1240 |  | 0.74 | 0.0119 | conserved hypothetical protein |
| SAUSA300_1257 | *msrR* | 0.70 | 0.0001 | peptide methionine sulfoxide reductase regulator MsrR |
| SAUSA300_1259 |  | 0.53 | 0.0000 | ImpB/MucB/SamB family protein |
| SAUSA300_1260 |  | 0.61 | 0.0000 | prephenate dehydrogenase |
| SAUSA300_1272 |  | 0.69 | 0.0013 | conserved hypothetical protein |
| SAUSA300_1286 |  | 0.37 | 0.0000 | aspartate kinase |
| SAUSA300_1287 | *asd* | 0.62 | 0.0000 | aspartate semialdehyde dehydrogenase |
| SAUSA300_1288 | *dapA* | 0.67 | 0.0002 | dihydrodipicolinate synthase |
| SAUSA300_1289 | *dapB* | 0.74 | 0.0099 | dihydrodipicolinate reductase |
| SAUSA300_1290 | *dapD* | 0.76 | 0.0121 | tetrahydrodipicolinate acetyltransferase |
| SAUSA300_1307 | *arlS* | 0.67 | 0.0000 | sensor histidine kinase protein |
| SAUSA300_1308 | *arlR* | 0.74 | 0.0144 | DNA-binding response regulator |
| SAUSA300_1327 |  | 0.72 | 0.0012 | cell surface protein |
| SAUSA300_1340 | *recU* | 0.78 | 0.0135 | recombination protein U |
| SAUSA300_1341 | *pbp2* | 0.73 | 0.0001 | penicillin binding protein 2 |
| SAUSA300_1371 | *recQ* | 0.74 | 0.0017 | ATP-dependent DNA helicase RecQ |
| SAUSA300_1372 | *recQ* | 0.63 | 0.0000 | conserved hypothetical protein |
| SAUSA300_1434 |  | 0.72 | 0.0231 | phiSLT ORF104a-like protein repressor |
| SAUSA300_1458 |  | 0.66 | 0.0033 | glyoxalase family protein |
| SAUSA300_1461 |  | 0.61 | 0.0031 | conserved hypothetical protein |
| SAUSA300_1481 |  | 0.64 | 0.0008 | putative membrane protein |
| SAUSA300_1499 | *aroK* | 0.70 | 0.0018 | shikimate kinase |
| SAUSA300_1512 | *pbp3* | 0.72 | 0.0003 | penicillin-binding protein 3 |
| SAUSA300_1522 | *dnaG* | 0.75 | 0.0008 | DNA primase |
| SAUSA300_1525 | *glyS* | 0.52 | 0.0000 | glycyl-tRNA synthetase |
| SAUSA300_1535 | *rpsU* | 0.34 | 0.0000 | 30S ribosomal protein S21 |
| SAUSA300_1549 |  | 0.68 | 0.0204 | ComE operon protein 1 |
| SAUSA300_1550 |  | 0.71 | 0.0006 | conserved hypothetical protein |
| SAUSA300_1558 | *mtnN* | 0.73 | 0.0129 | 5'-methylthioadenosine/S-adenosylhomocysteine nucleosidase |
| SAUSA300_1560 |  | 0.72 | 0.0028 | conserved hypothetical protein |
| SAUSA300_1606 |  | 0.47 | 0.0000 | conserved hypothetical protein |
| SAUSA300_1613 |  | 0.52 | 0.0000 | putative abrB protein |
| SAUSA300_1626 | *rpmI* | 0.78 | 0.0390 | 50S ribosomal protein L35 |
| SAUSA300_1627 | *infC* | 0.72 | 0.0058 | translation initiation factor IF-3 |
| SAUSA300_1671 |  | 0.75 | 0.0007 | conserved hypothetical protein |
| SAUSA300_1683 |  | 0.72 | 0.0005 | chorismate mutase/phospho-2-dehydro-3-deoxyheptonate aldolase |
| SAUSA300_1692 |  | 0.77 | 0.0420 | conserved hypothetical protein |
| SAUSA300_1706 |  | 0.61 | 0.0022 | conserved hypothetical protein |
| SAUSA300_1707 |  | 0.61 | 0.0044 | conserved hypothetical protein |
| SAUSA300_1712 | *ribH* | 0.49 | 0.0000 | riboflavin synthase beta subunit |
| SAUSA300_1713 | *ribBA* | 0.47 | 0.0000 | riboflavin biosynthesis protein |
| SAUSA300_1714 | *ribE* | 0.50 | 0.0000 | riboflavin synthase alpha subunit |
| SAUSA300_1715 | *ribD* | 0.62 | 0.0003 | riboflavin biosynthesis protein |
| SAUSA300_1722 |  | 0.62 | 0.0021 | conserved hypothetical protein |
| SAUSA300_1723 |  | 0.38 | 0.0000 | conserved hypothetical protein |
| SAUSA300_1724 |  | 0.48 | 0.0000 | abortive infection protein family |
| SAUSA300_1730 | *metK* | 0.25 | 0.0000 | S-adenosylmethionine synthetase |
| SAUSA300_1732 |  | 0.58 | 0.0000 | putative transposase |
| SAUSA300_1758 | *splA* | 0.58 | 0.0148 | serine protease SplA |
| SAUSA300_1775 |  | 0.46 | 0.0029 | tRNA-Gly |
| SAUSA300_1776 |  | 0.66 | 0.0161 | tRNA-His |
| SAUSA300_1777 |  | 0.60 | 0.0079 | tRNA-Phe |
| SAUSA300_1778 |  | 0.73 | 0.0224 | tRNA-Asp |
| SAUSA300_1785 |  | 0.78 | 0.0387 | putative ABC transporter protein EcsB |
| SAUSA300_1790 | *prsA* | 0.59 | 0.0000 | foldase protein PrsA precursor |
| SAUSA300_1809 |  | 0.71 | 0.0089 | putative membrane protein |
| SAUSA300_1810 |  | 0.45 | 0.0000 | IS1181 transposase |
| SAUSA300_1811 |  | 0.56 | 0.0382 | tRNA-Leu |
| SAUSA300_1812 |  | 0.35 | 0.0000 | tRNA-Gly |
| SAUSA300_1813 |  | 0.48 | 0.0000 | tRNA-Gly |
| SAUSA300_1814 |  | 0.55 | 0.0000 | tRNA-Cys |
| SAUSA300_1817 |  | 0.67 | 0.0152 | tRNA-Trp |
| SAUSA300_1818 |  | 0.57 | 0.0002 | tRNA-Tyr |
| SAUSA300_1819 |  | 0.66 | 0.0005 | tRNA-Thr |
| SAUSA300_1820 |  | 0.64 | 0.0016 | tRNA-Phe |
| SAUSA300_1821 |  | 0.63 | 0.0001 | tRNA-Asp |
| SAUSA300_1823 |  | 0.53 | 0.0000 | tRNA-Ser |
| SAUSA300_1824 |  | 0.69 | 0.0030 | tRNA-Asp |
| SAUSA300_1827 |  | 0.67 | 0.0158 | tRNA-Met |
| SAUSA300_1828 |  | 0.69 | 0.0022 | tRNA-Ala |
| SAUSA300_1829 |  | 0.67 | 0.0053 | tRNA-Pro |
| SAUSA300_1830 |  | 0.47 | 0.0000 | tRNA-Arg |
| SAUSA300_1831 |  | 0.45 | 0.0000 | tRNA-Leu |
| SAUSA300_1832 |  | 0.68 | 0.0083 | tRNA-Gly |
| SAUSA300_1834 |  | 0.68 | 0.0118 | tRNA-Lys |
| SAUSA300_1835 |  | 0.55 | 0.0000 | tRNA-Thr |
| SAUSA300_1840 |  | 0.64 | 0.0075 | tRNA-Ile |
| SAUSA300_1842 |  | 0.68 | 0.0008 | transcriptional regulator Fur family |
| SAUSA300_1855 | *sgtB* | 0.61 | 0.0000 | monofunctional glycosyltransferase |
| SAUSA300_1865 | *vraR* | 0.67 | 0.0001 | DNA-binding response regulator |
| SAUSA300_1866 | *vraS* | 0.61 | 0.0000 | two-component sensor histidine kinase |
| SAUSA300_1867 |  | 0.60 | 0.0000 | conserved hypothetical protein |
| SAUSA300_1868 |  | 0.51 | 0.0000 | conserved hypothetical protein |
| SAUSA300_1875 |  | 0.70 | 0.0067 | exonuclease |
| SAUSA300_1877 |  | 0.74 | 0.0096 | conserved hypothetical protein |
| SAUSA300_1890 |  | 0.71 | 0.0320 | staphopain A |
| SAUSA300_1899 |  | 0.78 | 0.0382 | conserved hypothetical protein |
| SAUSA300_1900 | *ppaC* | 0.81 | 0.0280 | manganese-dependent inorganic pyrophosphatase |
| SAUSA300_1901 | *aldA2* | 0.76 | 0.0308 | aldehyde dehydrogenase |
| SAUSA300_1918 |  | 0.56 | 0.0000 | truncated beta-hemolysin |
| SAUSA300_1919 |  | 0.35 | 0.0000 | conserved hypothetical protein |
| SAUSA300_1920 | *chs* | 0.25 | 0.0000 | chemotaxis-inhibiting protein CHIPS |
| SAUSA300_1973 |  | 0.68 | 0.0039 | truncated beta-hemolysin |
| SAUSA300_1974 |  | 0.77 | 0.0445 | Leukocidin/Hemolysin toxin family protein |
| SAUSA300_1985 | *sdrH* | 0.80 | 0.0426 | serine-aspartate repeat family protein SdrH |
| SAUSA300_1988 |  | 0.60 | 0.0000 | delta-hemolysin precursor |
| SAUSA300_1992 | *agrA* | 0.77 | 0.0251 | accessory gene regulator protein A |
| SAUSA300_2019 |  | 0.51 | 0.0061 | tRNA-Leu |
| SAUSA300_2038 | *murF* | 0.80 | 0.0457 | UDP-N-acetylmuramoyl-tripeptide--D-alanyl-D- alanine ligase |
| SAUSA300_2039 | *ddl* | 0.76 | 0.0108 | D-alanine--D-alanine ligase |
| SAUSA300_2048 | *thiM* | 0.50 | 0.0001 | hydroxyethylthiazole kinase |
| SAUSA300_2049 | *thiD* | 0.51 | 0.0015 | phosphomethylpyrimidine kinase |
| SAUSA300_2050 | *thiD* | 0.44 | 0.0000 | TENA/THI-4 family protein |
| SAUSA300_2077 |  | 0.64 | 0.0000 | conserved hypothetical protein |
| SAUSA300_2078 | *murA* | 0.53 | 0.0000 | UDP-N-acetylglucosamine 1-carboxyvinyltransferase |
| SAUSA300_2085 |  | 0.64 | 0.0000 | conserved hypothetical protein |
| SAUSA300_2092 | *dps* | 0.63 | 0.0001 | general stress protein 20U |
| SAUSA300_2115 | *tnp* | 0.43 | 0.0000 | IS1181 transposase |
| SAUSA300_2117 |  | 0.64 | 0.0045 | tRNA-Gln |
| SAUSA300_2118 |  | 0.44 | 0.0000 | tRNA-Tyr |
| SAUSA300_2119 |  | 0.63 | 0.0112 | tRNA-Val |
| SAUSA300_2126 |  | 0.57 | 0.0000 | drug resistance transporter EmrB/QacA subfamily |
| SAUSA300_2129 |  | 0.80 | 0.0233 | putative hemolysin III |
| SAUSA300_2132 |  | 0.32 | 0.0001 | conserved hypothetical protein |
| SAUSA300_2134 |  | 0.78 | 0.0291 | iron compound ABC transporter permease protein |
| SAUSA300_2135 |  | 0.81 | 0.0336 | iron compound ABC transporter permease protein |
| SAUSA300_2136 |  | 0.74 | 0.0337 | iron compound ABC transporter iron compound-binding protein |
| SAUSA300_2140 |  | 0.74 | 0.0050 | conserved hypothetical protein |
| SAUSA300_2167 |  | 0.75 | 0.0025 | conserved hypothetical protein |
| SAUSA300_2176 |  | 0.72 | 0.0124 | ABC transporter ATP-binding protein |
| SAUSA300_2216 |  | 0.52 | 0.0019 | transcriptional regulator MarR family |
| SAUSA300_2217 |  | 0.65 | 0.0144 | putative drug transporter |
| SAUSA300_2225 | *moaC* | 0.59 | 0.0000 | molybdenum cofactor biosynthesis protein C |
| SAUSA300_2245 |  | 0.38 | 0.0000 | staphylococcal accessory regulator R |
| SAUSA300_2247 |  | 0.66 | 0.0108 | staphylococcal accessory regulator |
| SAUSA300_2248 |  | 0.72 | 0.0162 | transcriptional regulator AraC family |
| SAUSA300_2262 |  | 0.51 | 0.0000 | putative membrane protein |
| SAUSA300_2264 |  | 0.70 | 0.0001 | phosphosugar-binding transcriptional regulator RpiR family |
| SAUSA300_2269 |  | 0.78 | 0.0489 | conserved hypothetical protein |
| SAUSA300_2270 | *glvC* | 0.52 | 0.0000 | PTS system arbutin-like IIBC component |
| SAUSA300_2273 |  | 0.65 | 0.0012 | Na+/H+ antiporter family protein |
| SAUSA300_2291 | *gltS* | 0.43 | 0.0000 | sodium/glutamate symporter |
| SAUSA300_2294 |  | 0.75 | 0.0375 | conserved hypothetical protein |
| SAUSA300_2297 |  | 0.78 | 0.0189 | conserved hypothetical protein |
| SAUSA300_2300 |  | 0.64 | 0.0025 | transcriptional regulator TetR family |
| SAUSA300_2302 | *tcaA* | 0.75 | 0.0047 | teicoplanin resistance associated membrane protein TcaA protein |
| SAUSA300_2303 | *tcaR* | 0.49 | 0.0000 | transcriptional regulator TcaR |
| SAUSA300_2326 |  | 0.43 | 0.0000 | transcription regulatory protein |
| SAUSA300_2331 |  | 0.63 | 0.0004 | transcriptional regulator MarR family |
| SAUSA300_2351 |  | 0.34 | 0.0000 | Zn-binding lipoprotein adcA-like protein |
| SAUSA300_2352 |  | 0.67 | 0.0006 | addiction module toxin Txe/YoeB family |
| SAUSA300_2353 |  | 0.73 | 0.0092 | conserved hypothetical protein |
| SAUSA300_2354 |  | 0.78 | 0.0136 | putative lipoprotein |
| SAUSA300_2355 |  | 0.69 | 0.0028 | putative lipoprotein |
| SAUSA300_2356 | *fmhA* | 0.70 | 0.0102 | fmhA protein |
| SAUSA300_2363 |  | 0.63 | 0.0033 | cation efflux family protein |
| SAUSA300_2364 | *sbi* | 0.26 | 0.0000 | IgG-binding protein SBI |
| SAUSA300_2365 | *hlgA* | 0.52 | 0.0003 | gamma-hemolysin component A |
| SAUSA300_2366 | *hlgC* | 0.56 | 0.0454 | gamma-hemolysin component C |
| SAUSA300_2367 | *hlgB* | 0.44 | 0.0078 | gamma-hemolysin component B |
| SAUSA300_2377 |  | 0.69 | 0.0012 | glycerate kinase |
| SAUSA300_2378 |  | 0.64 | 0.0000 | conserved hypothetical protein |
| SAUSA300_2379 |  | 0.74 | 0.0024 | putative transporter protein |
| SAUSA300_2385 |  | 0.77 | 0.0034 | putative membrane protein |
| SAUSA300_2386 |  | 0.71 | 0.0056 | beta-lactamase |
| SAUSA300_2387 |  | 0.76 | 0.0355 | NAD dependent epimerase/dehydratase family protein |
| SAUSA300_2402 |  | 0.76 | 0.0187 | conserved hypothetical protein |
| SAUSA300_2406 |  | 0.19 | 0.0000 | putative transporter |
| SAUSA300_2407 |  | 0.18 | 0.0000 | oligopeptide ABC transporter ATP-binding protein |
| SAUSA300_2408 |  | 0.17 | 0.0000 | oligopeptide ABC transporter ATP-binding protein |
| SAUSA300_2409 |  | 0.17 | 0.0000 | oligopeptide ABC transporter permease protein |
| SAUSA300_2410 |  | 0.23 | 0.0000 | oligopeptide ABC transporter permease protein |
| SAUSA300_2411 | *opp* | 0.19 | 0.0000 | oligopeptide permease peptide-binding protein |
| SAUSA300_2412 |  | 0.08 | 0.0000 | conserved hypothetical protein |
| SAUSA300_2413 |  | 0.09 | 0.0000 | conserved hypothetical protein |
| SAUSA300_2414 |  | 0.08 | 0.0000 | conserved hypothetical protein |
| SAUSA300_2441 | *fnbA* | 0.61 | 0.0000 | fibronectin binding protein A |
| SAUSA300_2453 |  | 0.62 | 0.0015 | ABC transporter ATP-binding protein |
| SAUSA300_2454 |  | 0.56 | 0.0000 | membrane spanning protein |
| SAUSA300_2456 |  | 0.73 | 0.0246 | putative membrane protein |
| SAUSA300_2461 |  | 0.57 | 0.0000 | glyoxalase family protein |
| SAUSA300_2464 |  | 0.63 | 0.0003 | hydrolase haloacid dehalogenase-like family |
| SAUSA300_2469 | *sdaAA* | 0.73 | 0.0065 | L-serine dehydratase iron-sulfur-dependent alpha subunit |
| SAUSA300_2470 | *sdaAB* | 0.56 | 0.0000 | L-serine dehydratase iron-sulfur-dependent beta subunit |
| SAUSA300_2471 |  | 0.63 | 0.0002 | perfringolysin O regulator protein |
| SAUSA300_2484 |  | 0.67 | 0.0003 | hydroxymethylglutaryl-CoA synthase |
| SAUSA300_2490 |  | 0.73 | 0.0120 | regulatory protein TetR family |
| SAUSA300_2491 |  | 0.68 | 0.0370 | 1-pyrroline-5-carboxylate dehydrogenase |
| SAUSA300_2493 |  | 0.25 | 0.0000 | conserved hypothetical protein |
| SAUSA300_2509 |  | 0.63 | 0.0083 | transcriptional regulator TetR family |
| SAUSA300_2520 |  | 0.61 | 0.0166 | transporter gate domain protein |
| SAUSA300_2521 |  | 0.56 | 0.0112 | conserved hypothetical protein |
| SAUSA300_2562 |  | 0.67 | 0.0057 | conserved hypothetical protein |
| SAUSA300_2563 |  | 0.74 | 0.0033 | putative transcriptional regulator |
| SAUSA300_2565 | *clfB* | 0.69 | 0.0272 | clumping factor B |
| SAUSA300_2567 | *arcC* | 0.66 | 0.0016 | carbamate kinase |
| SAUSA300_2575 |  | 0.57 | 0.0000 | transcriptional antiterminator BglG family |
| SAUSA300_2576 |  | 0.41 | 0.0000 | phosphotransferase system fructose-specific IIABC component |
| SAUSA300_2577 | *manA* | 0.37 | 0.0000 | mannose-6-phosphate isomerase class I |
| SAUSA300_2579 |  | 0.49 | 0.0000 | N-acetylmuramoyl-L-alanine amidase domain protein |
| SAUSA300_2580 |  | 0.75 | 0.0323 | isochorismatase family protein |
| SAUSA300_2599 | *tetR* | 0.79 | 0.0382 | intercellular adhesion operon transcription regulator TetR family |
| SAUSA300_2604 |  | 0.25 | 0.0080 | conserved hypothetical protein |
| SAUSA300_2605 | *hisIE* | 0.23 | 0.0051 | histidine biosynthesis bifunctional protein hisIE |
| SAUSA300_2606 | *hisF* | 0.24 | 0.0039 | imidazole glycerol phosphate synthase subunit hisF |
| SAUSA300_2607 | *hisA* | 0.22 | 0.0021 | phosphoribosylformimino-5-aminoimidazole carboxamide ribotide isomerase hisA |
| SAUSA300_2608 | *hisH* | 0.24 | 0.0083 | imidazole glycerol phosphate synthase subunit hisH |
| SAUSA300_2609 | *hisB* | 0.20 | 0.0011 | imidazole glycerol phosphate dehydratase hisB |
| SAUSA300_2610 | *hisC* | 0.19 | 0.0014 | histidinol-phosphate aminotransferase hisC |
| SAUSA300_2611 | *hisD* | 0.18 | 0.0002 | histidinol dehydrogenase hisD |
| SAUSA300_2612 | *hisG* | 0.20 | 0.0006 | ATP phosphoribosyltransferase hisG |
| SAUSA300_2613 |  | 0.20 | 0.0002 | conserved hypothetical protein |
| SAUSA300_2614 |  | 0.70 | 0.0138 | putative lipoprotein |
| SAUSA300_2616 |  | 0.67 | 0.0220 | cobalt transport family protein |
| SAUSA300_2617 |  | 0.64 | 0.0009 | putative cobalt ABC transporter ATP-binding protein |
| SAUSA300_2618 |  | 0.58 | 0.0008 | conserved hypothetical protein |
| SAUSA300_2619 |  | 0.54 | 0.0000 | conserved hypothetical protein |
| SAUSA300_2621 |  | 0.42 | 0.0000 | conserved hypothetical protein |
| SAUSA300_2648 | *rpmH* | 0.51 | 0.0000 | 50S ribosomal protein L34 |
