# Supplementary material for: Calprotectin Increases the Activity of the SaeRS Two Component System and Murine Mortality during Staphylococcus aureus Infections
Source: PLoS Pathog. 2015 Jul 6;11(7):e1005026. doi: 10.1371/journal.ppat.1005026 (PMC4492782; doi:10.1371/journal.ppat.1005026)
Supplement: S7 Table — (DOCX) [file ppat.1005026.s014.docx]

**S7 Table. Genes up-regulated by CP in the presence of Zn**

| **ID** | **Name** | **Fold Change** | **p value** | **SaeR binding sequence*** | **Gene Product** |
| --- | --- | --- | --- | --- | --- |
| SAUSA300_0370 |  | 1.47 | 0.0103 | **GTTAA**tcaaga**GTTAA** | enterotoxin |
| SAUSA300_0690 | *saeS* | 1.42 | 0.0022 | **GTTAA**gaatta**GTTAA** | sensor histidine kinase SaeS |
| SAUSA300_0691 | *saeR* | 1.38 | 0.0071 | **GTTAA**gaatta**GTTAA** | response regulator SaeR |
| SAUSA300_0692 | *saeQ* | 1.45 | 0.0006 | **GTTAA**gaatta**GTTAA** | SaeS regulator |
| SAUSA300_0693 | *saeP* | 1.39 | 0.0425 | **GTTAA**gaatta**GTTAA** | SaeS regulator |
| SAUSA300_0964 |  | 1.39 | 0.0371 | - | chitinase-related protein |
| SAUSA300_1015 | *ctaA* | 1.45 | 0.0041 | - | cytochrome oxidase assembly protein |
| SAUSA300_1016 | *cyoE* | 1.30 | 0.0409 | - | protoheme IX farnesyltransferase |
| SAUSA300_1055 | *efb* | 1.61 | 0.0020 | a**TTAA**taatta**GTTAA** | fibrinogen-binding protein |
| SAUSA300_1056 |  | 1.62 | 0.0034 | a**TTAA**taatta**GTTAA** | complement inhibitor |
| SAUSA300_1058 | *hla* | 1.58 | 0.0103 | **GTTAA**tatata**GTTAA** | alpha-hemolysin precursor |
| SAUSA300_1920 | *chs* | 1.70 | 0.0010 | t**TTAA**ttttta**GTTAA**  a**TTAA**tttcaa**GTTAA** | chemotaxis-inhibiting protein CHIPS |
| SAUSA300_2078 | *murA* | 1.40 | 0.0186 | - | UDP-N-acetylglucosamine 1-carboxyvinyltransferase |
| SAUSA300_2621 |  | 1.55 | 0.0015 | **GTTA**taagcat**GTTAA** | conserved hypothetical protein |

* GTTAAN6GTTAA, N = any nucleotide
