# Supplementary material for: Calprotectin Increases the Activity of the SaeRS Two Component System and Murine Mortality during Staphylococcus aureus Infections
Source: PLoS Pathog. 2015 Jul 6;11(7):e1005026. doi: 10.1371/journal.ppat.1005026 (PMC4492782; doi:10.1371/journal.ppat.1005026)
Supplement: S8 Table — (DOCX) [file ppat.1005026.s015.docx]

**S8 Table. Genes down-regulated by CP in the presence of Zn**

| **ID** | **Name** | **Fold Change** | **p value** | **Gene product** |
| --- | --- | --- | --- | --- |
| SAUSA300_0206 |  | 0.38 | 0.0000 | flavodoxin family protein |
| SAUSA300_0338 |  | 0.66 | 0.0371 | glyoxalase family protein |
| SAUSA300_0339 |  | 0.67 | 0.0269 | conserved hypothetical protein |
| SAUSA300_0435 |  | 0.49 | 0.0339 | ABC transporter ATP-binding protein |
| SAUSA300_0437 |  | 0.57 | 0.0134 | NLPA lipoprotein |
| SAUSA300_0445 | *gltB* | 0.72 | 0.0111 | glutamate synthase large subunit |
| SAUSA300_0507 | *ctsR* | 0.70 | 0.0042 | transcriptional regulator CtsR |
| SAUSA300_0508 |  | 0.76 | 0.0188 | conserved hypothetical protein |
| SAUSA300_0509 |  | 0.78 | 0.0298 | ATP guanido phosphotransferase |
| SAUSA300_0536 |  | 0.51 | 0.0001 | Chaperone protein HchA |
| SAUSA300_0576 |  | 0.46 | 0.0000 | putative Pyridine nucleotide-disulphide oxidoreductase |
| SAUSA300_0577 |  | 0.31 | 0.0000 | putative transcriptional regulator |
| SAUSA300_0752 | *clpP* | 0.73 | 0.0174 | ATP-dependent Clp protease proteolytic subunit ClpP |
| SAUSA300_0842 |  | 0.75 | 0.0179 | conserved hypothetical protein |
| SAUSA300_0859 |  | 0.70 | 0.0298 | NADH-dependent flavin oxidoreductase |
| SAUSA300_0877 | *clpB* | 0.66 | 0.0311 | Chaperone clpB |
| SAUSA300_0934 |  | 0.63 | 0.0363 | membrane protein |
| SAUSA300_0936 |  | 0.54 | 0.0247 | ABC transporter ATP-binding protein |
| SAUSA300_0943 |  | 0.60 | 0.0199 | acetyltransferase GNAT family family |
| SAUSA300_1227 | *thrC* | 0.77 | 0.0311 | threonine synthase |
| SAUSA300_1259 |  | 0.73 | 0.0339 | ImpB/MucB/SamB family protein |
| SAUSA300_1296 |  | 0.64 | 0.0175 | conserved hypothetical protein |
| SAUSA300_1525 | *glyS* | 0.71 | 0.0298 | glycyl-tRNA synthetase |
| SAUSA300_1580 |  | 0.66 | 0.0236 | bacterial luciferase family protein |
| SAUSA300_1659 | *tpx* | 0.76 | 0.0450 | thiol peroxidase |
| SAUSA300_1669 |  | 0.67 | 0.0096 | aminotransferase class V |
| SAUSA300_1670 | *serA* | 0.70 | 0.0058 | D-3-phosphoglycerate dehydrogenase |
| SAUSA300_1725 |  | 0.69 | 0.0041 | transaldolase |
| SAUSA300_1819 |  | 0.71 | 0.0369 | tRNA-Thr |
| SAUSA300_1833 |  | 0.64 | 0.0081 | tRNA-Leu |
| SAUSA300_1835 |  | 0.69 | 0.0303 | tRNA-Thr |
| SAUSA300_2139 |  | 0.66 | 0.0311 | putative transporter |
| SAUSA300_2145 |  | 0.49 | 0.0000 | glycine betaine transporter |
| SAUSA300_2358 |  | 0.69 | 0.0311 | ABC transporter permease protein |
| SAUSA300_2451 |  | 0.63 | 0.0488 | drug transporter |
| SAUSA300_2457 |  | 0.26 | 0.0000 | phospholipase/carboxylesterase family protein |
| SAUSA300_2458 |  | 0.22 | 0.0000 | glyoxylase family protein |
| SAUSA300_2461 |  | 0.63 | 0.0097 | glyoxalase family protein |
| SAUSA300_2475 |  | 0.70 | 0.0394 | conserved hypothetical protein |
| SAUSA300_2573 | *isaB* | 0.64 | 0.0301 | immunodominant antigen B |
| SAUSA300_2617 |  | 0.67 | 0.0336 | putative cobalt ABC transporter ATP-binding protein |
| SAUSA300_2619 |  | 0.59 | 0.0042 | conserved hypothetical protein |
| SAUSA300_2620 |  | 0.59 | 0.0138 | conserved hypothetical protein |
