# Supplementary material for: Calprotectin Increases the Activity of the SaeRS Two Component System and Murine Mortality during Staphylococcus aureus Infections
Source: PLoS Pathog. 2015 Jul 6;11(7):e1005026. doi: 10.1371/journal.ppat.1005026 (PMC4492782; doi:10.1371/journal.ppat.1005026)
Supplement: S9 Table — (DOCX) [file ppat.1005026.s016.docx]

# S9 Table. Bacterial strains and plasmids.

| Strain or plasmid | | Description | Source or reference |
| --- | --- | --- | --- |
| Strains | |  |  |
|  | *E. coli* |  |  |
|  | DH5a | Restriction deficient, host for cloning | NEB |
|  | BL21star(DE3) | Protein expression strain | NEB |
|  | *S. aureus* |  |  |
|  | RN4220 | Restriction deficient *S. aureus* | [[1](#_ENREF_1)] |
|  | Newman | A *S. aureus* strain carrying SaeS L18P mutation | [[2](#_ENREF_2)] |
|  | USA300-P23 | A USA300 strain without plasmid 2 and 3 | [[3](#_ENREF_3)] |
|  | USA300Δ*sae* | USA300-P23 with *sae* deletion | This study |
|  | NE99 | A transposon mutant of the *fur* gene | NARSA |
|  | USA300*fur* | USA300-P23 that acquired the *fur* transposon mutation in NE99 via transduction | This study |
| Plasmids | |  |  |
|  | pCL55 | An integration plasmid for *S. aureus* | [[4](#_ENREF_4)] |
|  | pCL-P1-*lacZ* | pCL55 carrying P1-*lacZ* transcriptional fusion | [[5](#_ENREF_5)] |
|  | pCL-*gfp* | pCL55 carrying *gfp* in the multi-cloning site | This study |
|  | pCL-P1*gfp* | pCL55 carrying P1-*gfp* transcriptional fusion | This study |
|  | pCL-*saeRS* | pCL55 containing the *saeRS* region | [[3](#_ENREF_3)] |
|  | pYJ335 | A multi-copy plasmid with an anhydrotetracycline-inducible promoter | [[6](#_ENREF_6)] |
|  | pYJ-*saeRS* | pYJ335 containing the saeRS region under the control of the anhydrotetracycline-inducible promoter | This study |

1. Kreiswirth BN, Lofdahl S, Betley MJ, O'Reilly M, Schlievert PM, et al. (1983) The toxic shock syndrome exotoxin structural gene is not detectably transmitted by a prophage. Nature 305: 709-712.

2. Duthie ES, Lorenz LL (1952) Staphylococcal coagulase; mode of action and antigenicity. J Gen Microbiol 6: 95-107.

3. Jeong DW, Cho H, Lee H, Li C, Garza J, et al. (2011) Identification of P3 promoter and distinct roles of the two promoters of the SaeRS two-component system in *Staphylococcus aureus*. J Bacteriol 193: 4672-4684.

4. Lee CY, Buranen SL, Ye ZH (1991) Construction of single-copy integration vectors for *Staphylococcus aureus*. Gene 103: 101-105.

5. Sun F, Li C, Jeong D, Sohn C, He C, et al. (2010) In the *Staphylococcus aureus* two-component system sae, the response regulator SaeR binds to a direct repeat sequence and DNA binding requires phosphorylation by the sensor kinase SaeS. J Bacteriol 192: 2111-2127.

6. Ji Y, Marra A, Rosenberg M, Woodnutt G (1999) Regulated antisense RNA eliminates alpha-toxin virulence in *Staphylococcus aureus* infection. J Bacteriol 181: 6585-6590.
