# Supplementary material for: Calprotectin Increases the Activity of the SaeRS Two Component System and Murine Mortality during Staphylococcus aureus Infections
Source: PLoS Pathog. 2015 Jul 6;11(7):e1005026. doi: 10.1371/journal.ppat.1005026 (PMC4492782; doi:10.1371/journal.ppat.1005026)
Supplement: S10 Table — (DOCX) [file ppat.1005026.s017.docx]

**S10 Table. Primers used for quantitative RT-PCR**

| Name | Sequence (5’ -> 3’) |
| --- | --- |
| 16S-F | CCTTACCAAATCTTGACATCC |
| 16S-R | GTGTAGCCCAAATCATAAGG |
| saeP-F | CAACCATTGCGATTTCTTTACC |
| saeP-R | TTAGCTTTAGGTGCTTGTGG |
| coa-F | CTACGGCTGGTAAAGCTGAAG |
| coa-R | TGAGCTACCTTCAAGACCTTC |
| hla-F | GGCGGCCTTATTGGTGCAAATG |
| hla-R | CCATATACCGGGTTCCAAGA |
| fnbA-F | GGAGCAGCATCAGTATTCTT |
| fnbA-R | AGTTGCAGTTGTTTGTGTTT |
| sak-F | TGTAGTCCCAGGTTTAATAGG |
| sak-R | CGCGAGTTATTTTGAACC |
| lukS-PV-F | ACCCCCATTAGTACACAGTG |
| lukS-PV-R | CTTCTAGTAGCATGAGTAACATC |
| psmα-F | GGCCATTCACATGGAATTCGT |
| psmα-R | GCCATCGTTTTGTCCTCCTG |
| spa-F | TGGCTTGGATCATCTTTAAGG |
| spa-R | CATTACTTATATCTGGTGGCGT |
